# Functional hierarchy of the angular gyrus and its underlying genetic architecture

**DOI:** 10.1002/hbm.26247

**Published:** 2023-02-28

**Authors:** Yu Song, Chunli Wang, Huanhuan Cai, Jingyao Chen, Siyu Liu, Jiajia Zhu, Yongqiang Yu

**Affiliations:** ^1^ Department of Radiology The First Affiliated Hospital of Anhui Medical University Hefei China; ^2^ Research Center of Clinical Medical Imaging, Anhui Province Hefei China; ^3^ Anhui Provincial Institute of Translational Medicine Hefei China; ^4^ Department of Clinical Laboratory The First Affiliated Hospital of Anhui Medical University Hefei China

**Keywords:** angular gyrus, functional gradient, functional MRI, gene expression, hierarchical organization

## Abstract

The angular gyrus (AG), given its rich connectivity and its location where multisensory information converges, is a functionally and anatomically heterogeneous structure. Using the state‐of‐the‐art functional gradient approach and transcription‐neuroimaging association analysis, we sought to determine whether there is an overarching hierarchical organization of the AG and if so, how it is modulated by the underlying genetic architecture. Resting‐state functional MRI data of 793 healthy subjects were obtained from discovery and validation datasets. Functional gradients of the AG were calculated based on the voxel‐wise AG‐to‐cerebrum functional connectivity patterns. Combined with the Allen Human Brain Atlas, we examined the spatial correlations between the AG functional gradient and gene expression. The dominant gradient topography showed a dorsoanterior–ventroposterior hierarchical organization of the AG, which was related to its intrinsic geometry. Concurrently, AG functional subdivisions corresponding to canonical functional networks (behavioral domains) were distributed along the dominant gradient in a hierarchical manner, that is, from the default mode network (abstract cognition) at one extreme to the visual and sensorimotor networks (perception and action) at the other extreme. Remarkably, we established a link between the AG dominant gradient and gene expression, with two gene sets strongly contributing to this link but diverging on their functional annotation and specific expression. Our findings represent a significant conceptual advance in AG functional organization, and may introduce novel approaches and testable questions to the investigation of AG function and anatomy in health and disease.

## Graphical abstract

We comprehensively characterized the overarching hierarchical organization of the angular gyrus and its underlying genetic architecture by applying a combined approach of the functional gradient and transcription‐neuroimaging association to large‐scale discovery and validation rs‐fMRI datasets.

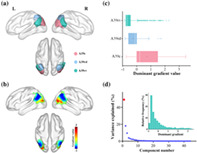

## 1 INTRODUCTION

The angular gyrus (AG), located in the posterior part of the inferior parietal lobule where multisensory (auditory, visual, and somatosensory) information converges (Seghier, 2013), is a functionally heterogeneous structure that has been implicated in a wide variety of cognitive domains, including memory (Bonnici et al., 2018; Humphreys et al., 2021; Yazar et al., 2017), theory of mind (Mar, 2011; Schurz et al., 2017), visuospatial attention (Cattaneo et al., 2009), arithmetic (Rosenberg‐Lee et al., 2011), and semantic processing (Davey et al., 2015; Lewis et al., 2019). The functional heterogeneity of the AG is assumed to rely on its complex cytoarchitectonic and connectivity characteristics, based on which the AG has been parcellated into anatomically different subdivisions (Caspers et al., 2006; 2008; Mars et al., 2011; Ruschel et al., 2014; Uddin et al., 2010; Wang et al., 2012). Moreover, numerous neuroimaging studies have documented structural and functional abnormalities of the AG in various neuropsychiatric diseases (Pico‐Perez et al., 2022; Wang et al., 2018; Yuksel et al., 2018; Zeng et al., 2021). Collectively, this previous literature raises the question of whether there is an overarching hierarchical organization of the AG that underlies these findings in basic and clinical neuroscience and if so, how it is modulated by the genetic architecture.

Advances in resting‐state functional magnetic resonance imaging (rs‐fMRI) have made it feasible to measure the temporal correlations of blood‐oxygen‐level‐dependent (BOLD) signals between spatially distinct brain regions, referred to as resting‐state functional connectivity (rsFC) (Barkhof et al., 2014; Fox & Raichle, 2007). By applying a functional gradient approach to high‐dimensional rsFC data, Margulies and colleagues have described a dominant gradient in human cortical organization that spans between sensorimotor and transmodal areas, pointing to a central principle for understanding the cerebral cortex that macroscale anatomy reflects a functional hierarchy from primary perception and action to more abstract cognitive functions (Margulies et al., 2016). The functional gradient analysis provides an updated framework for simplifying the complex rsFC matrix to a small set of gradients that captures more gradual changes and overarching spatial relationships of rsFC patterns (Huntenburg et al., 2018). Benefiting from these advantages, the functional gradient approach has recently been leveraged to investigate the topographic organization of multiple brain structures, including the cerebellum (Guell et al., 2018), thalamus (Yang et al., 2020), hippocampus (Vos de Wael et al., 2018), subcortex (Tian et al., 2020), and insula (Tian & Zalesky, 2018; R. Wang et al., 2022). Meanwhile, some functional gradients have been shown to associate with intrinsic geometry, correspond to canonical functional networks, and subserve specific behavioral domains (Margulies et al., 2016; Yang et al., 2020). Despite these promising findings, there is a dearth of research examining the basic hierarchical organization of the AG using a combination of the functional gradient method and rsFC data.

The advent of comprehensive, brain‐wide gene expression atlases (e.g., the Allen Human Brain Atlas (AHBA); Hawrylycz et al., 2012) has provided the unprecedented capacity to link macroscale brain organization to microscale molecular pathways through a combined analysis of neuroimaging phenotypes and gene expression profiles (Arnatkevic̆iūtė, Fulcher, & Fornito, 2019; Fornito et al., 2019). In this instance comes the most commonly used approach, transcription‐neuroimaging association analysis that can help to identify genes with spatial profiles of regional expression that track anatomical variations in a certain neuroimaging biomarker (Liu et al., 2022). By use of this powerful approach, prior studies have achieved great success in unraveling the genetic underpinnings of rsFC (Anderson et al., 2018; Chen et al., 2022; Richiardi et al., 2015; Shen et al., 2022; Zhang et al., 2021; Zhu et al., 2021). Of more importance, there have been several attempts to reveal the molecular substrates underlying functional connectivity gradient alterations in major depression (Xia et al., 2022a) and during development (Xia et al., 2022b), highlighting the potential usefulness of transcription‐neuroimaging association analysis in elucidating the genetic mechanisms underlying functional gradients.

**FIGURE 1:**
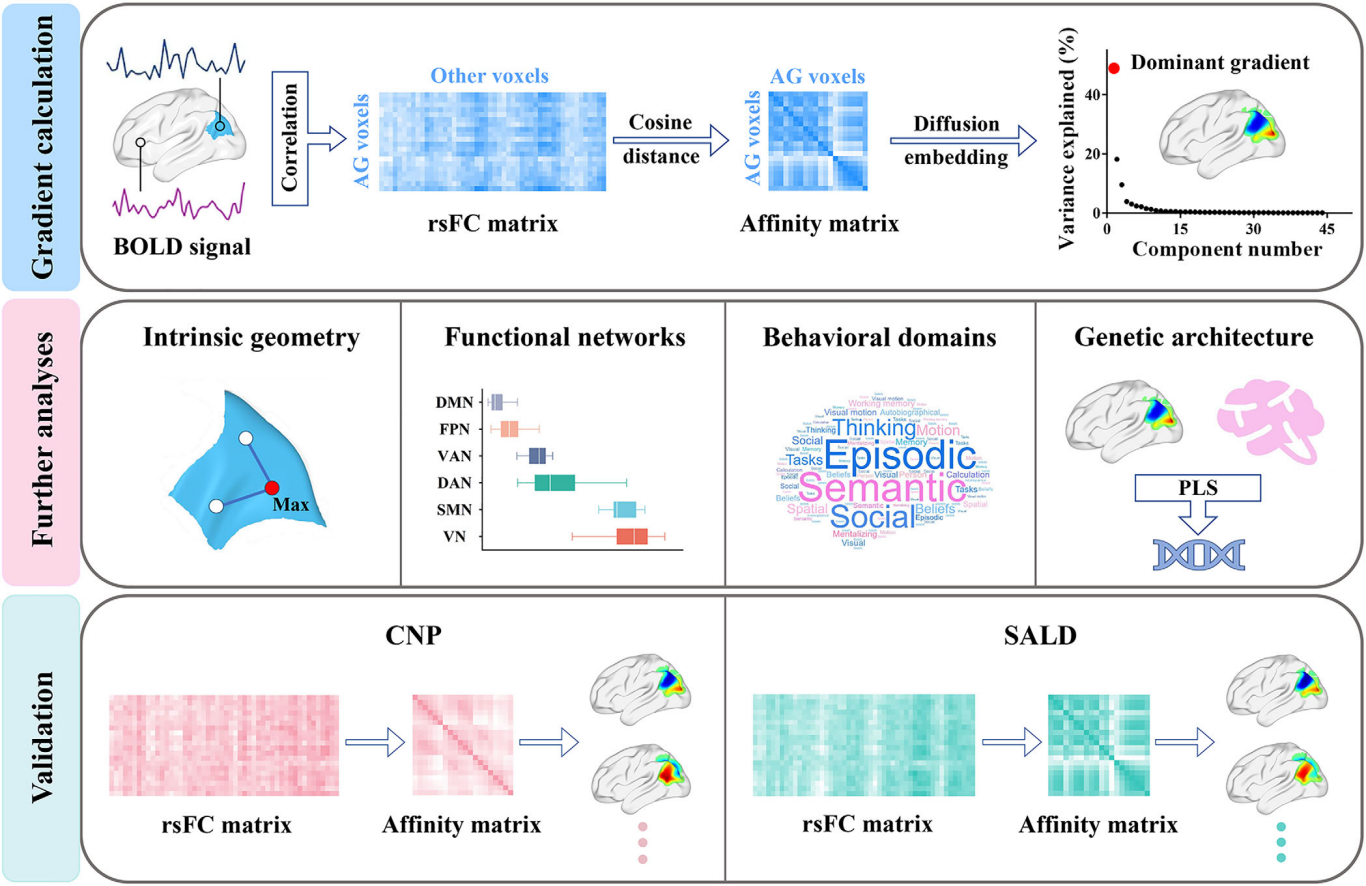
Flowchart of the study design and analysis pipeline. Top panel: gradient calculation. We collected rs‐fMRI data from a large discovery sample of 361 healthy subjects. Functional gradients of the AG were calculated based on the voxel‐wise AG‐to‐cerebrum rsFC patterns using diffusion map embedding. We focused on the first gradient (i.e., the dominant gradient) that accounted for the greatest variance in connectivity. Middle panel: further analyses. We examined the relations of the AG dominant functional gradient to intrinsic geometry, canonical functional networks, and behavioral domains. In conjunction with the AHBA, we conducted transcriptome‐neuroimaging spatial correlation analysis to investigate the genetic architecture underlying the dominant gradient. Bottom panel: validation. The robustness of our results was verified in two independent cross‐race, cross‐scanner validation datasets (CNP and SALD). AG, angular gyrus; BOLD, blood‐oxygen‐level‐dependent; CNP, Consortium for Neuropsychiatric Phenomics; DAN, dorsal attention network; DMN, default mode network; FPN, frontoparietal network; Max, maximum; PLS, partial least squares; SALD, Southwest University Adult Lifespan Dataset; SMN, sensorimotor network; rsFC, resting‐state functional connectivity; rs‐fMRI, resting‐state functional magnetic resonance imaging; VAN, ventral attention network; VN, visual network.

In this study, we sought to determine the hierarchical organization of the AG and its underlying genetic architecture by using the state‐of‐the‐art functional gradient approach and transcription‐neuroimaging association analysis. To realize this goal, we collected rs‐fMRI data from a large discovery sample of 361 healthy subjects. Functional gradients of the AG were then calculated based on the voxel‐wise AG‐to‐cerebrum rsFC patterns, followed by examination of their relations to intrinsic geometry, canonical functional networks, and behavioral domains. In conjunction with the AHBA, we further conducted transcriptome‐neuroimaging spatial correlation analysis to identify genes whose expression measures were associated with the AG functional gradient, with functional annotation and specific expression analyses pursued to characterize their biological significance. Finally, we verified the robustness of our results in two independent cross‐race, cross‐scanner validation datasets. A schematic overview of the study design and analysis pipeline is provided in Figure 1.

## 2 MATERIALS AND METHODS

### 2.1 Participants

The current study included a discovery dataset and two independent cross‐race, cross‐scanner validation datasets. For the discovery dataset, we enrolled healthy adults of Chinese Han and right‐handedness from the local universities and community through poster advertisements. Participants were excluded if they had neuropsychiatric or severe somatic disorder, a history of head injury with loss of consciousness, pregnancy, MRI contraindications, a family history of psychiatric illness among first‐degree relatives, and gross abnormalities on brain MRI images. All experimental procedures were approved by the ethics committee of The First Affiliated Hospital of Anhui Medical University and written informed consent was provided by each participant. The two validation datasets were obtained from two publically available neuroimaging datasets: the Consortium for Neuropsychiatric Phenomics (CNP, https://openneuro.org/datasets/ds000030/versions/1.0.0) (Poldrack et al., 2016) and the Southwest University Adult Lifespan Dataset (SALD, https://doi.org/10.15387/fcp_indi.sald) (Wei et al., 2018). It is notable that we exclusively chose healthy adults from the cross‐disorder CNP dataset. For the CNP dataset, the healthy participants were excluded if they had lifetime diagnoses of psychiatric disorders, left handedness, pregnancy, or other contraindications to scanning; for the SALD dataset, the exclusion criteria included MRI contraindications, current psychiatric or neurological disorders, use of psychiatric drugs within 3 months, pregnancy, or a history of head trauma. Full details on the two validation datasets (e.g., informed consent, ethics, inclusion and exclusion criteria, among others) have been described in the previous data descriptor publications (Poldrack et al., 2016; Wei et al., 2018). To exclude the potential influence of neurodevelopment and neurodegeneration, all the participants were restricted to an age range of 18–60 years. Furthermore, we excluded participants with poor image quality or excessive head motion during scanning. The initial sample sizes of the discovery, CNP and SALD datasets were 362, 107, and 435, respectively. Through the above‐described screening procedure, the final samples used in this work were 361 (female/male: 183/178; mean and range of age: 28.84 ± 10.83, 18–30 years) in the discovery dataset, 103 (47/56; 30.87 ± 8.56, 21–50 years) in the CNP dataset, and 329 (207/122; 37.81 ± 13.79, 19–59 years) in the SALD dataset. Demographic data of the three datasets can be found in Table S1.

### 2.2 Image acquisition

MRI data of the discovery sample were acquired using the 3.0‐Tesla General Electric Discovery MR750w scanner, and those of the validation samples were both collected using the 3.0‐Tesla Siemens Trio scanners. The rs‐fMRI protocols for the three datasets are detailed in Table S2.

### 2.3 fMRI data preprocessing

Resting‐state BOLD data were preprocessed using Statistical Parametric Mapping (SPM12, http://www.fil.ion.ucl.ac.uk/spm) and Data Processing & Analysis for Brain Imaging (DPABI, http://rfmri.org/dpabi) (Yan et al., 2016). The first several volumes (discovery: 10, CNP: 5, SALD: 10) for each participant were removed to allow the signal to reach equilibrium and the participants to adapt to the scanning noise. The remaining volumes were corrected for the acquisition time delay between slices. Then, realignment was performed to correct the motion between time points. Head motion parameters were computed by estimating the translation in each direction and the angular rotation on each axis for each volume. All BOLD data were within the defined motion thresholds (i.e., translational or rotational motion parameters <2.0 mm or 2.0°). We also calculated frame‐wise displacement (FD), which indexes the volume‐to‐volume changes in head position. Several nuisance covariates (the linear drift, the estimated motion parameters based on the Friston‐24 model, the spike volumes with FD >0.5, the white matter signal, and the cerebrospinal fluid signal) were regressed out from the data. The datasets were then band‐pass filtered using a frequency range of 0.01–0.1 Hz. In the normalization step, individual structural images were firstly co‐registered with the mean functional images; then the transformed structural images were segmented and normalized to the Montreal Neurological Institute (MNI) space using the diffeomorphic anatomical registration through the exponentiated Lie algebra (DARTEL) technique (Ashburner, 2007). Finally, each filtered functional volume was spatially normalized to the MNI space using the deformation parameters estimated during the above step and resampled into a 3‐mm cubic voxel.

### 2.4 Calculation of AG functional gradients

**FIGURE 2:**
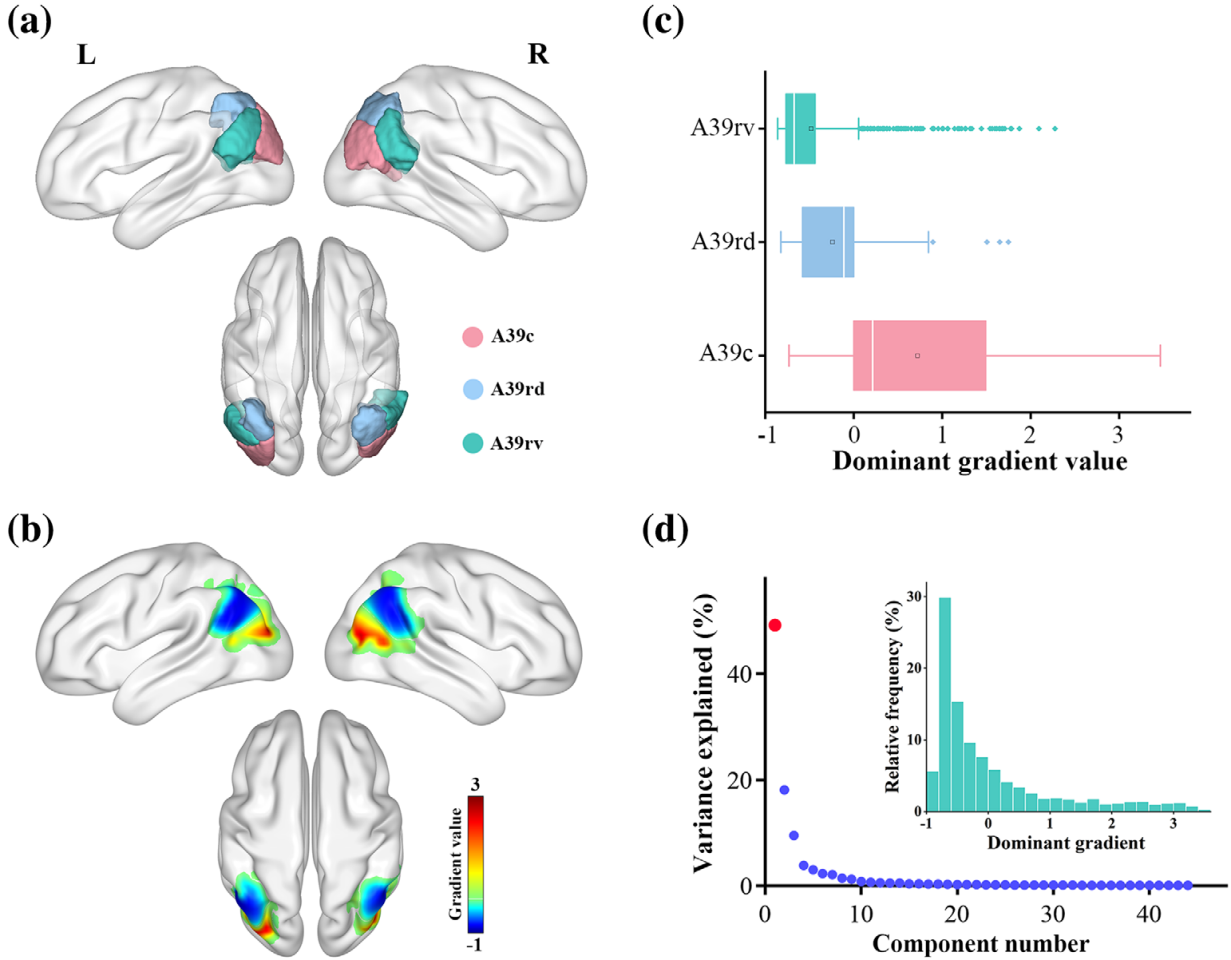
Functional gradients of the AG. (a) Illustration of AG subregions. (b) Topography of the dominant gradient. (c) Distribution of AG subregions along the dominant gradient. (d) Variance explained by the functional gradients. Inserted histogram demonstrated the distribution of the dominant gradient values of voxels within the AG. AG, angular gyrus; A39c, caudal area 39; A39rd, rostrodorsal area 39; A39rv, rostroventral area 39; L, left; R, right.

Functional gradients of the AG were calculated based on its rsFC to the entire cerebrum (Figure 1). First, the Human Brainnetome Atlas (Fan et al., 2016), a new brain atlas constructed using a connectivity‐based parcellation framework, was adopted to define the AG (2089 voxels) including caudal area 39 (A39c), rostrodorsal area 39 (A39rd), and rostroventral area 39 (A39rv) in each hemisphere (Figure 2a). Second, the preprocessed BOLD images were concatenated across all subjects after standardization using *z*‐scores, yielding group‐level BOLD time courses. Third, based on the group‐level BOLD time courses, a voxel‐wise AG‐to‐cerebrum rsFC matrix (2089 × 39,356) was generated by calculating Pearson's correlation coefficients between time courses of each voxel within the AG and each voxel within the cerebrum (excluding the AG), followed by Fisher's *Z*‐transformation to improve normality. Then, for each row in the rsFC matrix, the values of the top 10% of connections were retained, whereas all others were zeroed (Dong et al., 2021; Margulies et al., 2016; Yang et al., 2020). Of note, the negative connections were zeroed as well because their biological relevance remains controversial (Xia et al., 2022b). Fourth, similarity between all pairs of rows was calculated using cosine distance, resulting in a positive and symmetric affinity matrix representing similarity of connectivity profiles between each pair of voxels within the AG.

We calculated AG functional gradients using diffusion map embedding (Coifman et al., 2005; Guell et al., 2018; Margulies et al., 2016), a nonlinear dimensionality reduction technique that can recover a low‐dimensional embedding from high‐dimensional connectivity data. In the embedding space, voxels that are strongly connected by either many connections or few very strong connections are close, whereas voxels with little or no connections are far apart (Hong et al., 2019; Margulies et al., 2016). Relative to other non‐linear dimensionality reduction algorithms, diffusion map embedding is relatively robust to noise, computationally inexpensive, and provides a stable representation of connections (Lafon & Lee, 2006). By applying this algorithm to the affinity matrix, we identified multiple low‐dimensional gradients explaining connectivity variance in descending order. For each gradient, a gradient value was assigned to each voxel within the AG, resulting in an AG gradient map to visualize macroscale transitions in overall connectivity patterns, that is, the gradient topography. We focused on the first gradient (i.e., the dominant gradient) that accounted for the greatest variance in connectivity. Of note, the diffusion map embedding is controlled by a single parameter α, which controls the influence of the density of sampling points on the underlying manifold (α = 0, maximal influence; α = 1, no influence). In accordance with previous studies (Hong et al., 2019; Margulies et al., 2016; Yang et al., 2020), we set α = 0.5 that is considered well‐suited for the analysis of brain connectivity data.

### 2.5 Relation to intrinsic geometry

Having characterized the topography of the dominant gradient, we next investigated whether it was related to intrinsic geometry of the AG. To this end, we calculated the Euclidean distance between the peak voxel of the dominant gradient map and the remaining voxels within the AG. Then, cross‐voxel Pearson's correlation coefficient between the dominant gradient and the Euclidean distance was calculated to index how the dominant gradient changed with spatial distance from the peak voxel. A nonparametric permutation test was utilized to determine the statistical significance of the correlation. Briefly, we randomly shuffled the voxels within the AG 5000 times (i.e., 5000 permutations) and repeated the above‐described gradient‐distance correlation using the shuffled data. We recorded the gradient‐distance correlation coefficient in each permutation to build a null distribution. Based on the null distribution, the *p* value was calculated as the number of permutations that generated correlation coefficients greater than the true correlation coefficient/5000. Notably, the relationship between the dominant gradient and spatial distance was assessed in each hemisphere, separately.

### 2.6 Relation to functional networks

The association between the AG dominant functional gradient and canonical functional networks from the seven‐network parcellation (Yeo et al., 2011) was evaluated. An AG functional atlas was initially created using a custom winner‐take‐all parcellation approach (Greene et al., 2014; Seitzman et al., 2020; Zhang et al., 2008). That is, we calculated Pearson's correlation coefficient between BOLD time course of a given voxel within the AG and the average BOLD time course of each functional network. This AG voxel was then assigned to the functional network with the highest Pearson's correlation coefficient. This procedure was repeated for all voxels within the AG, which generated an AG functional atlas including seven functional subdivisions corresponding to seven canonical functional networks. For each subdivision, we extracted the dominant gradient values and sorted them by the mean.

### 2.7 Relation to behavioral domains

To capture the behavioral relevance of the AG dominant functional gradient, we investigated its associations with behavioral domains from the NeuroSynth (http://www.neurosynth.org), a well‐validated and publicly available platform for large‐scale automated synthesis of human neuroimaging data (Yarkoni et al., 2011). The NeuroSynth database provides activation (*z*‐statistics) maps of 1335 behavioral terms that describe nearly all aspects of human behavior. To establish a link between gradient and behavior, the AG dominant gradient map was binned into 10‐percentile increments and then binarized, yielding 10 binary masks ranging from 0–10% to 90%–100%. For each behavioral term, the average *z*‐statistics within the 10 masks were extracted. The terms with *z*‐statistic >2.3 were ordered for visualization.

### 2.8 Genetic architecture underlying the dominant gradient

Brain gene expression data were obtained from the AHBA dataset (http://www.brainmap.org) (Hawrylycz et al., 2012), which was derived from six human post‐mortem donors (Table S3). The original expression data of more than 20,000 genes at 3702 spatially distinct brain tissue samples were processed using a newly proposed pipeline (Arnatkevic̆iūtė et al., 2019). The detailed processing steps are described in the Supplementary Materials. After brain gene expression data processing, we obtained normalized expression data of 5013 genes for 1280 tissue samples across the left cerebral cortex. We further restricted our analyses to the samples within the AG, yielding a final sample × gene matrix of 25 × 5013.

To derive the dominant gradient value of a given brain tissue sample, we defined a 3 mm radius sphere centered at the MNI coordinate of this sample and extracted the average gradient value of voxels within the sphere from the dominant gradient map. This procedure was repeated for all samples within the AG, resulting in a dominant gradient vector of 25 samples. Then, the spatial associations between gene expression and the dominant gradient were examined using partial least squares (PLS) regression, a multivariate statistical technique that can predict a set of dependent variables from a set of independent variables (Abdi, 2010). PLS regression is technically well suited to the high collinearity of gene expression data and has been extensively applied to transcriptome‐neuroimaging association research (Hansen et al., 2021; Romero‐Garcia et al., 2020; Thomas et al., 2021). In our analysis, the gene expression matrix was defined as the independent variables, and the dominant gradient vector was designated as the dependent variables. PLS regression identified the first PLS component where the weighted sum of gene expression (gene scores) were most strongly correlated with the dominant gradient. Pearson's correlation coefficient between the gene scores and the dominant gradient was calculated, and its statistical significance was assessed using permutation testing by randomly shuffling the samples 5000 times.

Gene contribution was estimated with gene loading, that is, Pearson's correlation coefficient between an individual gene's expression pattern and PLS‐derived gene scores across the tissue samples. Then, genes were segregated based on the sign of their loadings, where positive genes refer to genes with positive loadings and negative genes refer to those with negative loadings. Strongly contributing genes were defined as those among the top 25% of the positive and negative genes, which were termed PLS+ and PLS− genes.

To better understand the biological significance of PLS+ and PLS− genes, we conducted several gene enrichment analyses including functional annotation and specific expression. First, functional annotation was performed with the ToppGene portal (https://toppgene.cchmc.org/) (Chen et al., 2009). Gene ontology (GO) was utilized to determine the biological functions including molecular functions (MFs), biological processes (BPs), and cellular components (CCs). The pathway and disease databases were used to determine the biological pathways and diseases. Second, we used online tissue‐specific expression analysis (TSEA) (http://genetics.wustl.edu/jdlab/tsea/) and cell type‐specific expression analysis (CSEA) (http://genetics.wustl.edu/jdlab/csea-tool-2/) (Dougherty et al., 2010) tools to determine the tissues, cortical cell types, and developmental stages in which PLS+ and PLS− genes were specifically expressed. A specificity index probability (*p*SI = 0.05) was used to index how genes are more enriched in specific terms relative to others (Xu et al., 2014). For the above‐mentioned enrichment analyses, Fisher's exact tests were used to evaluate their statistical significance. Multiple testing was corrected using the Benjamini and Hochberg method for false discovery rate (FDR‐BH correction) with a corrected *p* value of .05.

### 2.9 Validation analyses

Several validation analyses were performed to verify the robustness of our results. First, to test the effect of network sparsity, we repeated the functional gradient analyses by applying two other thresholds (top 20% and 30%) to the rsFC matrix. Second, since global signal regression (GSR) has long been a controversial topic in rs‐fMRI analyses (Murphy & Fox, 2017), we re‐computed the functional gradients using BOLD data with GSR. Third, to test lateralization effects in the AG (Seghier, 2013), we examined the associations of the AG dominant functional gradient with canonical functional networks and behavior domains for the left and right AG, separately. Finally, our main analyses were conducted in the discovery dataset. To exclude the influence of samples, we also carried out the above‐mentioned analyses in two independent cross‐race, cross‐scanner validation datasets (CNP and SALD).

## 3 RESULTS

### 3.1 The dominant functional gradient of the AG

The variance in AG rsFC patterns explained by the functional gradients is shown in descending order (Figure 2d). The dominant (first) gradient accounted for the greatest variance (49%) and global histogram demonstrated the distribution of the dominant gradient values of voxels within the AG (Figure 2d). The topography of the dominant gradient showed a dorsoanterior‐ventroposterior organization (Figure 2b), characterized by a gradual increase from the dorsoanterior (A39rv and A39rd) to the ventroposterior (A39c) portion of the AG (Figure 2c).

### 3.2 Relation to intrinsic geometry

**FIGURE 3:**
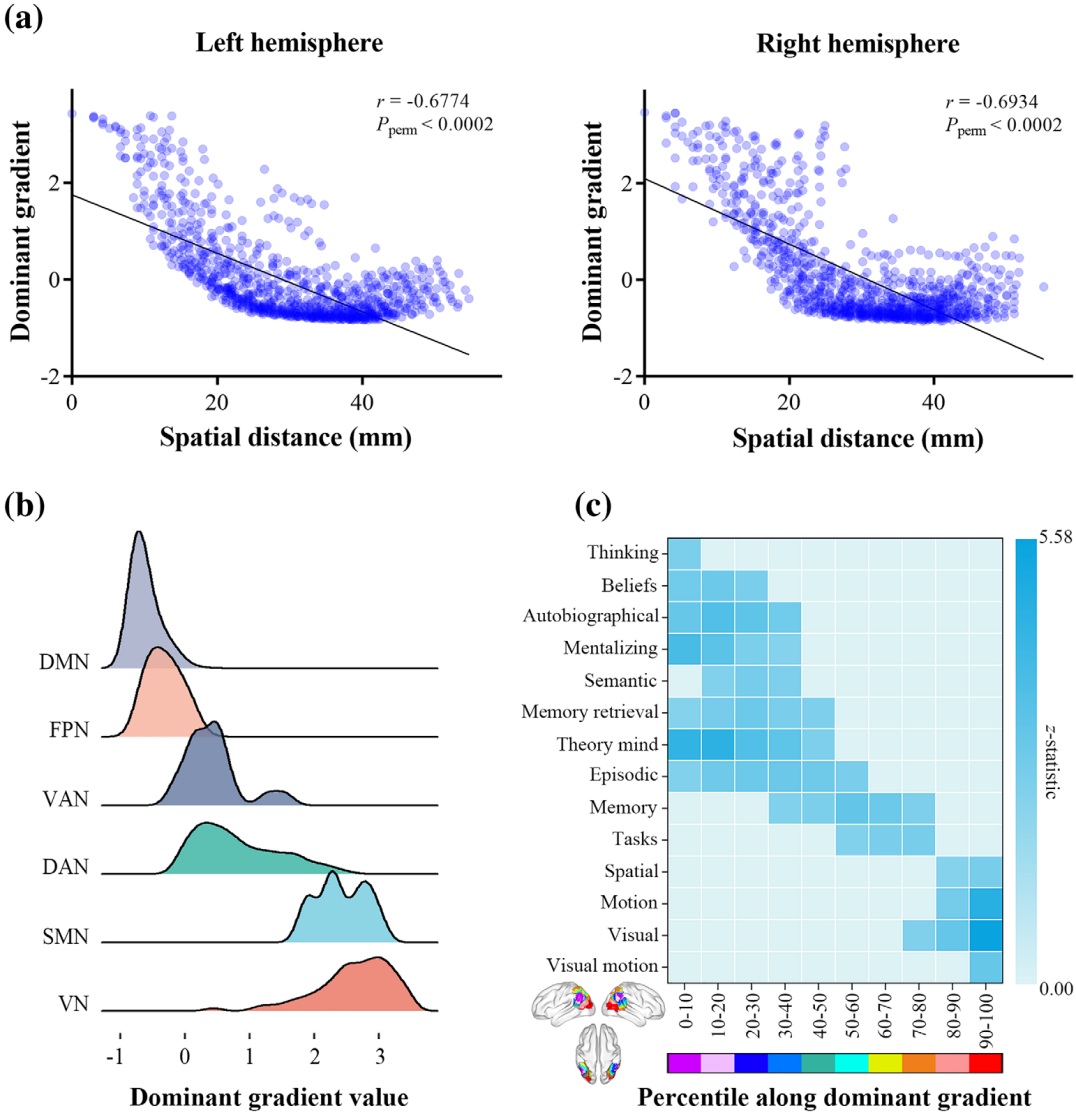
Relations of the AG dominant functional gradient to intrinsic geometry, functional networks, and behavioral domains. (a) Associations between the dominant gradient and spatial distance from the peak voxel in both hemispheres. (b) Distribution of the AG functional subdivisions corresponding to the canonical functional networks along the dominant gradient. The functional subdivision corresponding to the limbic network was not found. (c) Associations between the dominant gradient and behavioral terms from the NeuroSynth. AG, angular gyrus; DAN, dorsal attention network; DMN, default mode network; FPN, frontoparietal network; SMN, sensorimotor network; VN, visual network; VAN, ventral attention network.

Cross‐voxel Pearson's correlation analysis revealed a negative association between the dominant gradient and spatial distance from the peak voxel in both hemispheres (left: *r* = −0.6774, *p*_perm_ <.0002; right: *r* = −0.6934, *p*_perm_ <.0002) (Figure 3a), indicating that the dominant gradient was related to intrinsic geometry of the AG.

### 3.3 Relation to functional networks

The AG functional subdivisions corresponding to the canonical functional networks were not randomly distributed along the dominant gradient, but rather tended to cluster at similar positions (Figure 3b). Importantly, the functional subdivision corresponding to the default mode network occupied one extreme position along the dominant gradient and was maximally separated from those corresponding to the visual and sensorimotor networks, which were at the other extreme.

### 3.4 Relation to behavioral domains

Behavioral relevance of the AG dominant functional gradient was characterized by use of the NeuroSynth. We found 14 behavioral terms exhibiting a systematic shift in function along the dominant gradient, echoing the above‐described results of functional network analysis. The end involving the default mode network was related to terms describing abstract cognition such as “thinking”, “beliefs”, “autobiographical” and “mentalizing”, whereas the other end involving the visual and sensorimotor networks was linked to terms depicting perception and action such as “visual” and “motion” (Figure 3c).

### 3.5 Genetic architecture underlying the dominant gradient

**FIGURE 4:**
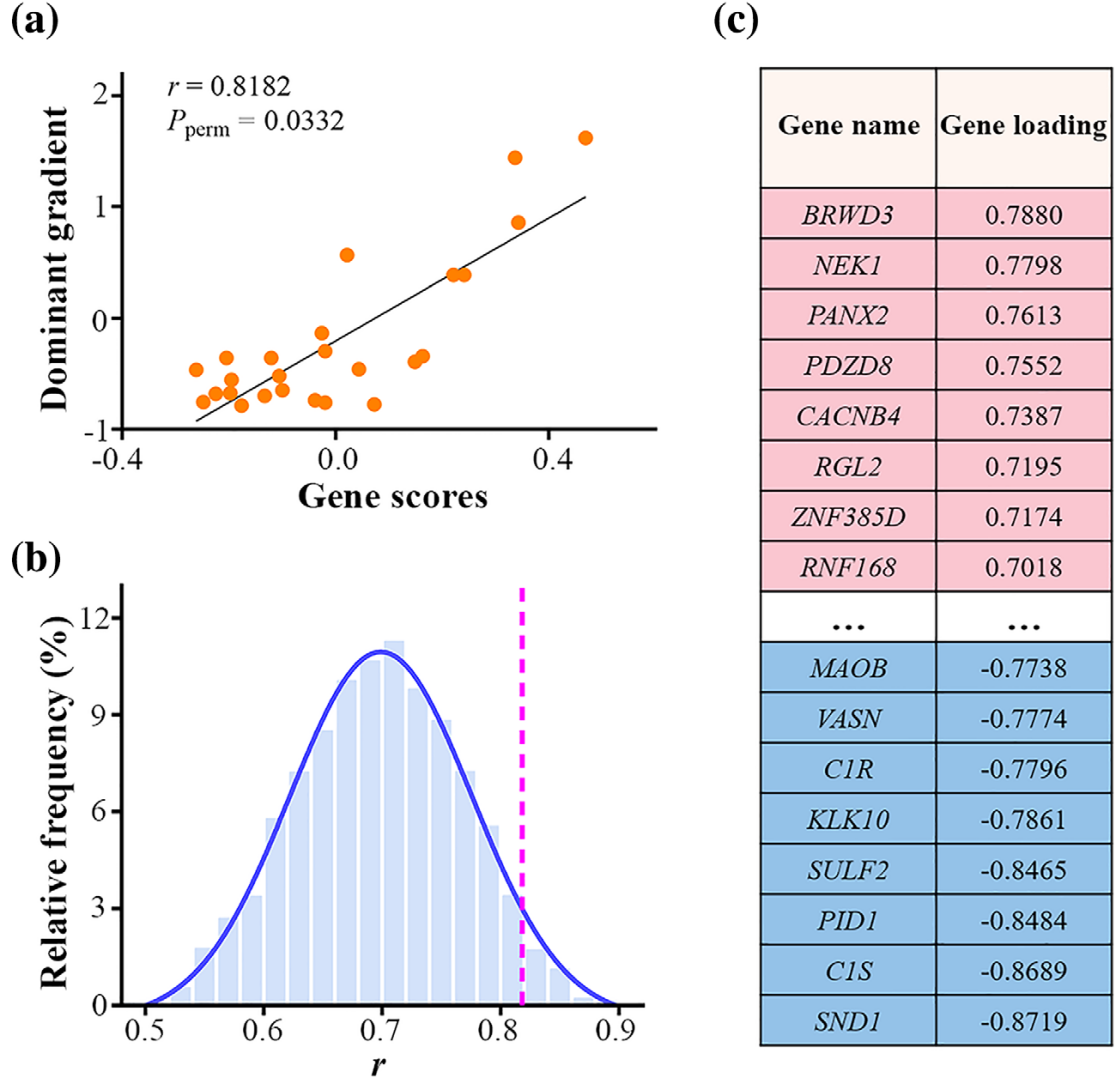
Gene expression and the AG dominant functional gradient. (a) Scatterplot of gene scores (identified by PLS regression) versus the dominant gradient with each point representing a tissue sample within the AG. (b) Histogram of permutation distribution showing that the correlation (dotted line) between gene scores and the dominant gradient was significantly greater than expected by chance. (c) Illustrative example of the loadings (gene contributions) assigned to representative genes. Genes with the highest positive loadings are colored in pink and those with the lowest negative loadings are colored in blue. AG, angular gyrus; PLS, partial least squares.

PLS regression identified the first PLS component where the weighted sum of gene expression (gene scores) were most strongly correlated with the dominant gradient (*r* = 0.8182) (Figure 4a), which was significantly greater than expected by chance (*p*_perm_ = .0332) (Figure 4b). All the 5013 genes were ranked by their contributions to the correlation (i.e., gene loadings) (Figure 4c and File S1). 497 PLS+ genes (the top 25% of genes with positive loadings) and 756 PLS− genes (the top 25% of genes with negative loadings) were defined as strongly contributing genes.

**FIGURE 5:**
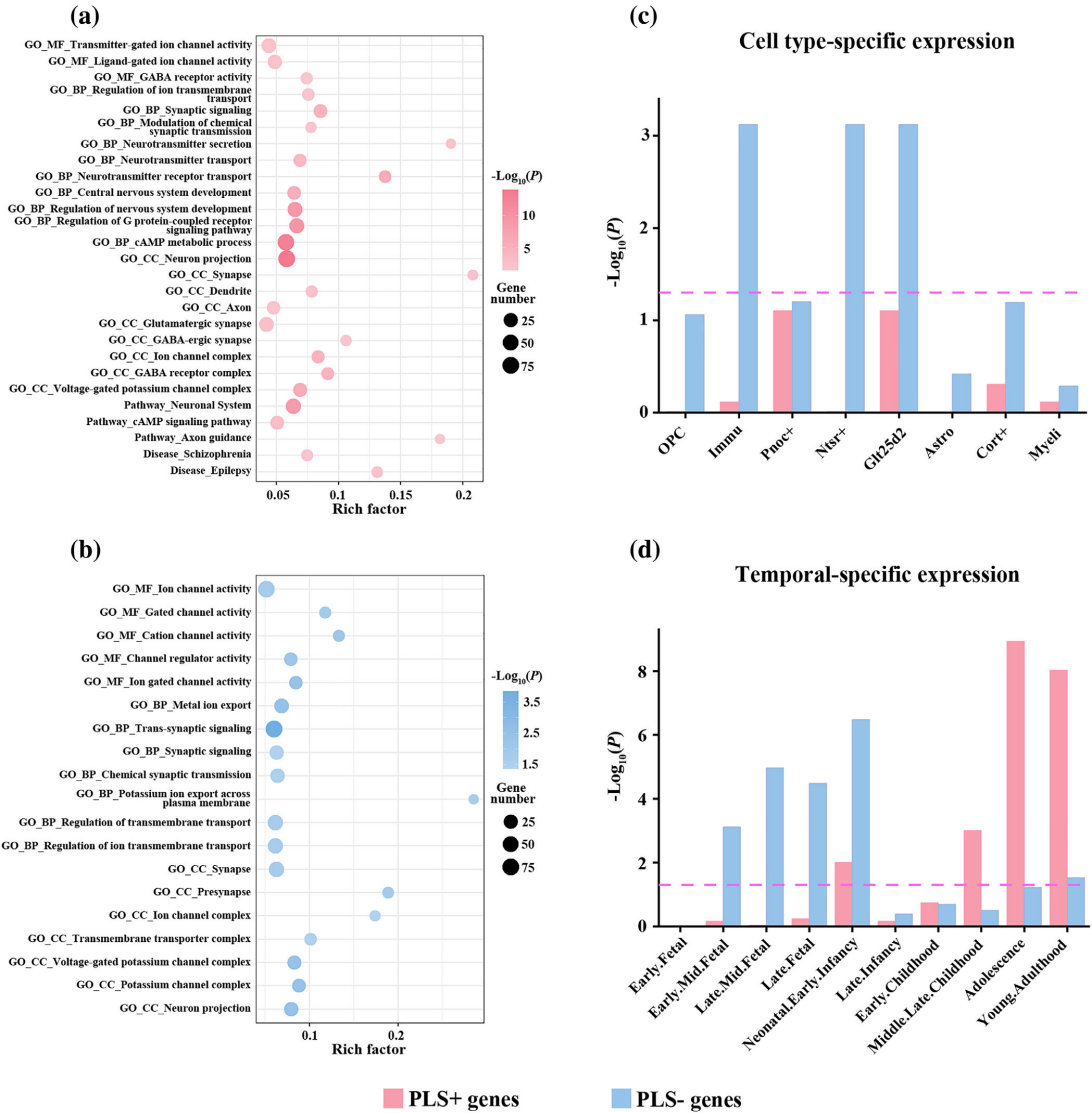
Enrichment analyses of the strongly contributing genes. Functional annotation of 497 PLS+ genes (the top 25% of genes with positive loadings) (a) and 756 PLS− genes (the top 25% of genes with negative loadings) (b). For each bubble chart, the x‐axis denotes the rich factor and the y‐axis denotes items from the GO, pathway, and disease databases. The rich factor refers to the ratio of the number of PLS+ or PLS− genes annotated to the item to the number of all genes annotated to the item. The bubble size represents the number of genes overlapping with those belonging to each item, and the bubble color represents the ‐Log_10_(*p*) with the *p* value corrected by the FDR‐BH method. (c) Cell type‐specific expression. (d) Temporal‐specific expression. The y‐axis is ‐Log_10_(*p*) with the *p* value corrected by the FDR‐BH. The purple dotted line represents the statistical significance threshold of *p* = .05. Astro, astrocytes; BP, biological process; CC, cellular component; Cort+, cortistatin‐expressing interneurons; FDR‐BH, the Benjamini and Hochberg method for false discovery rate; GO, gene ontology; Glt25d2, corticopontine neurons; Immu, immune cells; MF, molecular function; Myeli, myelinating oligodendrocytes; Nstr+, corticothalamic neurons; OPC, oligodendrocyte progenitor cells; PLS, partial least squares; Pnoc+, prepronociceptin‐expressing neurons.

To better understand the biological significance of PLS+ and PLS− genes, we conducted gene enrichment analyses including functional annotation and specific expression. The results of functional annotation for the two gene sets are listed in File S2 and are depicted in Figure 5a, b. Specifically, PLS+ genes were mainly enriched for MFs including ion channel activity and GABA receptor activity, BPs including synaptic signaling, synaptic transmission, neurotransmitter secretion, neurotransmitter transport, central nervous system development, G protein‐coupled receptor signaling pathway, and CCs including neuron projection, synapse, dendrite, axon, ion channel complex, and GABA receptor complex; for pathways including cAMP signaling pathway and axon guidance; and for diseases including schizophrenia and epilepsy (Figure 5a). PLS− genes were primarily enriched for MFs including ion channel activity, BPs including synaptic signaling, synaptic transmission and ion transmembrane transport, and CCs including neuron projection, synapse and ion channel complex (Figure 5b). The results of specific expression for PLS+ and PLS− genes are listed in File S3 and are illustrated in Figure 5c, d. Tissue‐specific expression analysis revealed that both the two gene sets showed specific expression in the brain tissue. Cell type‐specific expression analysis demonstrated that PLS+ genes were not specifically expressed in any cortical cell types, while PLS− genes were specifically expressed in Ntsr+ and Glt25d2 neurons as well as immune cells (Figure 5c). Temporal‐specific expression analysis showed that PLS+ genes were preferentially expressed during neonatal and early infancy, middle and late childhood, adolescence, and young adulthood, while PLS− genes were preferentially expressed during early mid‐fetal, late mid‐fetal, late fetal, neonatal and early infancy, and young adulthood (Figure 5d).

### 3.6 Validation analyses

First, by applying two other thresholds (top 20% and 30%) to the rsFC matrix, we observed that topography of the dominant gradient was nearly identical to that using the threshold of top 10% (Figure S3), indicating no effect of network sparsity. Second, analyzing BOLD data with GSR produced the dominant gradient similar to that in our main analysis of BOLD data without GSR, suggesting no influence of GSR (Figure S4). Third, the observed associations of the AG dominant functional gradient with canonical functional networks and behavior domains for the bilateral AG were largely preserved for the left and right AG, suggesting very little impact of lateralization on our results (Figures S5 and S6). Finally, the results based on two independent cross‐race, cross‐scanner validation datasets (CNP and SALD) were highly consistent with those from the discovery dataset. Specifically, the dominant gradient, explaining the greatest variance (CNP, 58%; SALD, 49%) (Figure S7B), showed the same topographic pattern (Figure S7A). Further cross‐voxel spatial correlation analyses demonstrated strikingly similar gradient distributions between the discovery and validation datasets (discovery vs. CNP: *r* = 0.9441, *p*_perm_ <.0002; discovery vs. SALD: *r* = 0.9642, *p*_perm_ <.0002) (Figure S7C). Moreover, consistent associations of the dominant gradient with intrinsic geometry, functional networks, and behavioral domains were found in CNP and SALD datasets (Figures S8 and S9). In addition, PLS regression identified significant correlations between gene expression and the dominant gradient in the validation datasets (CNP: *r* = 0.8303, *p*_perm_ = .0264; SALD: *r* = 0.8249, *p*_perm_ = .0284) (Figure S10). There were substantial overlaps between the strongly contributing genes identified in the discovery dataset and those identified in CNP (PLS+ genes: 92.35%; PLS− genes: 85.05%) and SALD (PLS+ genes: 93.76%; PLS− genes: 90.21%) datasets (Table S4).

## 4 DISCUSSION

Using a combined method of the rsFC‐based functional gradient and transcription‐neuroimaging association, we reported the first study to examine the hierarchical principle of AG macroscale organization and its underlying genetic architecture. The dominant gradient topography showed a dorsoanterior‐ventroposterior hierarchical organization of the AG, which was related to its intrinsic geometry. Concurrently, AG functional subdivisions corresponding to canonical functional networks (behavioral domains) were distributed along the dominant gradient in a hierarchical manner, that is, from the default mode network (abstract cognition) at one extreme to the visual and sensorimotor networks (perception and action) at the other extreme. Remarkably, further transcription‐neuroimaging spatial correlation analysis established a link between the AG dominant gradient and gene expression, with two gene sets (PLS+ and PLS− genes) strongly contributing to this link but diverging on their functional annotation and specific expression. These findings may provide new and fundamental insights into the hierarchical profile, biological and behavioral correlates, and genetic mechanisms of AG functional organization.

Using the functional gradient approach, prior studies have demonstrated the presence of an overarching hierarchical organization in multiple brain structures including the cerebral cortex (Margulies et al., 2016), cerebellum (Guell et al., 2018), thalamus (Yang et al., 2020), hippocampus (Vos de Wael et al., 2018), subcortex (Tian et al., 2020), and insula (Tian & Zalesky, 2018; Wang et al., 2022). The proposed functional gradient constitutes a core organizing axis of these brain structures, and describes an intrinsic coordinate system to integrate observations across time points, measurement modalities, subjects, and even across species (Huntenburg et al., 2018). Our finding of a dorsoanterior–ventroposterior organization captured by the AG dominant functional gradient topography adds to this existing literature. Furthermore, the dorsoanterior–ventroposterior hierarchy of the AG is largely consistent with the parcellation patterns based on cytoarchitecture, anatomical connectivity, or functional connectivity (Caspers et al., 2006, 2008; Mars et al., 2011; Ruschel et al., 2014; Uddin et al., 2010; Wang et al., 2012). This finding may complement and extend the current foundational knowledge about the heterogeneity of the AG from the perspective of functional hierarchy. The observed basic hierarchical organization of the AG may constitute a novel research agenda with potential applications in research and clinical settings.

The demonstration of the AG dominant gradient as a function of the spatial distance from the maximum suggests that the dominant gradient is closely related to intrinsic geometry of the AG, in support of the view that brain function is shaped and constrained by underlying structure (Honey et al., 2007; Honey et al., 2009). Earlier work has shown that the dominant functional gradient yields an organizing spatial framework for multiple large‐scale networks and characterizes a functional spectrum from perception and action to more abstract cognitive functions in the cerebral cortex (Margulies et al., 2016) and thalamus (Yang et al., 2020). Our data provide empirical evidence that this is also the case in the AG. While the AG has long been considered a key node of the default mode network (Buckner et al., 2008; Vatansever et al., 2017), our analysis revealed that AG functional subdivisions corresponding to six canonical functional networks were distributed along the dominant gradient in a hierarchical manner, that is, from the default mode network at one extreme to the visual and sensorimotor networks at the other extreme. In parallel, behavioral relevance analysis using the NeuroSynth database demonstrated a similar hierarchical distribution of the behavioral domains along the AG dominant gradient, that is, from abstract cognition at one end to perception and action at the other end. Indeed, with its location at the junction between the temporal, parietal, and occipital lobes, the AG emerges as a cross‐modal hub where converging multisensory information is combined and integrated to comprehend and give sense to events, manipulate mental representations, solve familiar problems, and reorient attention to relevant information (Seghier, 2013). Via its rich connectivity with other distributed systems, the AG also serves as a processing center that is critically involved in a broad range of cognitive functions including semantic processing, word reading and comprehension, number processing, default mode network, memory retrieval, attention and spatial cognition, reasoning, and social cognition (Seghier, 2013).

Further transcription‐neuroimaging spatial correlation analysis established a link between the dominant functional gradient and gene expression, indicating a genetic influence on the development of the hierarchical organization of the AG. Importantly, we identified two gene sets (PLS+ and PLS− genes) that strongly contributed to this link but diverged on their functional annotation results. Contrasting with PLS− genes enriched for relatively specialized biological functions including ion channels and synaptic transmission, PLS+ genes were enriched for more diverse biological functions and pathways as well as neuropsychiatric diseases. Regarding biological functions, PLS+ genes were mainly enriched for ion channels, synaptic transmission, and neurotransmitter systems. Ion channels are the important constituents of neurons, which are responsible for triggering nerve impulses and neurotransmitter's release (Kumar et al., 2016). Many neuropsychiatric disorders have been associated with the dysfunction of ion channels (D'Adamo et al., 2020; Kim, 2014; Kumar et al., 2016). Synaptic transmission is thought to play a crucial role in the exchange of information between neurons, which depends on neurotransmitter systems (Biederer et al., 2017; Hyman, 2005). With respect to pathways, PLS+ genes were primarily enriched for G protein‐coupled receptor signaling pathway, cAMP signaling pathway, and axon guidance. G protein‐coupled receptors are physiologically important membrane proteins that sense signaling molecules such as hormones and neurotransmitters (Venkatakrishnan et al., 2013). G protein‐coupled receptors and G protein signaling pathways regulate neuronal excitability, modulating fast acting neurotransmission mediated by ligand‐gated ion channels including glutamate and GABA receptors (Gerber et al., 2016; Rojas & Dingledine, 2013; Sakairi et al., 2020). Besides, it is well established that cAMP signaling pathway is mediated by G protein‐coupled receptors (Calebiro et al., 2010; Vilardaga et al., 2014). As the intracellular second messenger, cAMP relays into cells the information carried by hormones and neurotransmitters, thereby mediating the intracellular response to hormones and neurotransmitters (Zaccolo et al., 2021). Axon guidance allows the formation of intricate neural circuits by the precise navigation of growing axons to appropriate target regions, and abnormal expression of or mutation in axon guidance‐related genes can result in neuropsychiatric disorders (Kim & Kim, 2020; Robichaux & Cowan, 2014). As for diseases, PLS+ genes were found to be enriched in neuropsychiatric disorders including schizophrenia and epilepsy, emphasizing their clinical relevance, whereas this enrichment was absent for PLS− genes.

Tissue‐specific expression analysis revealed that both PLS+ and PLS− genes showed specific expression in the brain tissue, which confirms the credibility of our results. Nonetheless, the two gene sets differed significantly in cell types and developmental stages for which they were enriched. Cell type‐specific expression analysis demonstrated that PLS+ genes were not specifically expressed in any cortical cell types, yet PLS− genes were specifically expressed in Ntsr+ and Glt25d2 neurons as well as immune cells. The specific expression in neurons suggests the role of neurons in mediating the genetic influence on the hierarchical organization of the AG. It is generally accepted that neuro‐immune interactions are established and maintained in different tissues, particularly in the brain (Chu et al., 2020). In this context, immune cells (e.g., microglial cells) have been reported to hold the potential to regulate the development, structure, and function of neuronal networks in the brain (Garaschuk & Verkhratsky, 2019; Wolf et al., 2017). Temporal‐specific expression analysis showed that PLS− genes were preferentially expressed during the early stage of cortical development, a time when the most rapid growth of the brain occurs (Bethlehem et al., 2022) and neuroblasts migrate, grow processes, and form synaptic connections over time (Thomason, 2020). Conversely, PLS+ genes were preferentially expressed during the late stage of cortical development, a period when the brain undergoes dramatic reorganization and the risk for neuropsychiatric disorders increases sharply (Bethlehem et al., 2022; Paus et al., 2008).

Several limitations need to be considered when interpreting our results. First, our current work focused on the AG dominant functional gradient that explained 49% of connectivity variance. This may limit the capacity to achieve a more thorough characterization of the AG functional organization. Second, the functional gradients were calculated at the group level rather than at the individual level, with the aim of obtaining more stable functional gradients. In this instance, however, meaningful individual variation might be overlooked. Third, we were approximating real cortical distance with Euclidean distance. In the case of nodes in adjacent gyri, the Euclidean distance tends to underestimate the true cortical distance, since the white matter tracts often bend around the intervening sulcus (Sepulcre et al., 2010). Fourth, the AHBA contains data derived from only six postmortem brains. More comprehensive, brain‐wide microarray gene expression datasets are necessary. Fifth, there are limited gene expression data in the right hemisphere of the AHBA, such that only tissue samples in the left AG were analyzed, which may have introduced potential biases. Finally, the observed spatial correlations between functional gradient and gene expression do not inform mechanistic models, which warrant further investigation in future experimental animal studies.

To summarize, we comprehensively characterized the overarching hierarchical organization of the AG and its underlying genetic architecture by applying a combined approach of the functional gradient and transcription‐neuroimaging association to large‐scale discovery and validation rs‐fMRI datasets. Our findings represent a significant conceptual advance in AG functional organization, and may introduce novel approaches and testable questions to the investigation of AG function and anatomy in health and disease.

## CONFLICT OF INTEREST

The authors declare no conflict of interest.

## Supporting information


**DATA S1.** Supporting Information

**FILE S1.** Genes and gene loadings

**FILE S2.** Enrichment results of the PLS+ genes

**FILE S3.** Tissue‐specific expression for the PLS+ and PLS− genes

## Data Availability

In this study, we used brain imaging data from the Consortium for Neuropsychiatric Phenomics (CNP) (https://openneuro.org/datasets/ds000030/versions/1.0.0), and the Southwest University Adult Lifespan Dataset (SALD) (http://dx.doi.org/10.15387/fcp_indi.sald), and genetics data from the Allen Human Brain Atlas (AHBA) (http://www.brainmap.org). AHBA processing code is publicly accessible at https://github.com/BMHLab/AHBAprocessing/.
