# Supplementary material for: Functional hierarchy of the angular gyrus and its underlying genetic architecture
Source: Hum Brain Mapp. 2023 Feb 28;44(7):2815–28. doi: 10.1002/hbm.26247 (PMC10089092; doi:10.1002/hbm.26247)
Supplement: Supplementary file 1 — DATA S1. Supporting Information [file HBM-44-2815-s003.doc]

**Supplementary Materials**

**Materials and methods**

**Brain gene expression data processing**

Brain gene expression information was obtained from the publicly available Allen Human Brain Atlas (AHBA) dataset ([http://www.brain-map.org](http://www.brain-map.org/)) (M. Hawrylycz et al., 2015; M. J. Hawrylycz et al., 2012). The dataset was derived from six human post-mortem donors (Table S3 in the Supplementary Materials). Custom 64 K Agilent microarrays were used to measure the expression of more than 20,000 genes at 3,702 spatially distinct brain tissue samples. A newly proposed pipeline was employed to process gene expression data for transcriptome-neuroimaging associations in the current study (Arnatkevic̆iūtė, Fulcher, & Fornito, 2019). Schematic of a workflow for processing brain gene expression data is shown in Fig. S1. Specifically, we first updated the probe-to-gene annotations based on the latest available information from the National Center for Biotechnology Information (NCBI) using the Re-Annotator package (Arloth, Bader, Roh, & Altmann, 2015). With intensity-based filtering, we excluded probes that did not exceed the background noise in at least 50% of samples across all donors. As multiple probes were used to measure expression level of a single gene, we further used the RNA-seq data as a reference to select probes. After excluding genes that do not overlap between RNA-seq and microarray datasets, we calculated the correlations between microarray and RNA-seq expression measures for the remaining genes. After excluding probes with low correlations (*r* < 0.2), a representative probe for a gene was selected based on the highest correlation to the RNA-seq data. In this study, we only included the tissue samples in the left cerebral cortex. For one, all six donors had expression data in the left hemisphere, but only two donors had samples in the right hemisphere. For another, the inclusion of subcortical samples might introduce potential biases because of the great differences in gene expression between cortical and subcortical regions (M. J. Hawrylycz et al., 2012). To account for potential between-sample differences and donor-specific effects in gene expression, we performed both within-sample cross-gene (evaluating the relative expression level of all genes within a sample) and within-gene cross-sample (evaluating the relative expression level of a particular gene across different samples) normalization by using the scaled robust sigmoid normalization method. Differential stability (DS) is a measure of consistent regional variation across donor brains. Previous research has reported that genes with high DS scores demonstrate more consistent spatial expression patterns between donors (M. Hawrylycz et al., 2015). As gene expression conservation across subjects is a prerequisite for the transcriptome-neuroimaging spatial correlations, we only selected genes with relatively more conserved expression patterns for analysis. To realize this goal, we ranked the genes by their DS values and chose the 50% of the highest DS genes for the main analysis. After these processing procedures, we obtained normalized expression data of 5,013 genes for 1,280 tissue samples across the left cerebral cortex, resulting in a sample × gene matrix of 1,280 × 5,013. The numbers of remaining probes and genes at each processing step are shown in Fig. S2.

**References**

Arloth, J., Bader, D. M., Roh, S., & Altmann, A. (2015). Re-Annotator: Annotation Pipeline for Microarray Probe Sequences. *PLoS One, 10*(10), e0139516. doi:10.1371/journal.pone.0139516

Arnatkevic̆iūtė, A., Fulcher, B. D., & Fornito, A. (2019). A practical guide to linking brain-wide gene expression and neuroimaging data. *Neuroimage, 189*, 353-367. doi:10.1016/j.neuroimage.2019.01.011

Hawrylycz, M., Miller, J. A., Menon, V., Feng, D., Dolbeare, T., Guillozet-Bongaarts, A. L., . . . Lein, E. (2015). Canonical genetic signatures of the adult human brain. *Nat Neurosci, 18*(12), 1832-1844. doi:10.1038/nn.4171

Hawrylycz, M. J., Lein, E. S., Guillozet-Bongaarts, A. L., Shen, E. H., Ng, L., Miller, J. A., . . . Jones, A. R. (2012). An anatomically comprehensive atlas of the adult human brain transcriptome. *Nature, 489*(7416), 391-399. doi:10.1038/nature11405

**Supplementary tables**

**Table S1. Demographic information of the discovery and validation datasets**

| **Dataset** | **Sample size** | **Age (years)** | **Gender (F/M)** | **FD (mm)** |
| --- | --- | --- | --- | --- |
| Discovery | 361 | 28.84 ± 10.83 (18-30) | 183/178 | 0.13 ± 0.07 |
| CNP | 103 | 30.87 ± 8.56 (21-50) | 47/56 | 0.17 ± 0.08 |
| SALD | 329 | 37.81 ± 13.79 (19-59) | 207/122 | 0.15 ± 0.08 |

Age is expressed as mean ± standard deviation (range). FD is expressed as mean ± standard deviation. Abbreviations: CNP, Consortium for Neuropsychiatric Phenomics; SALD, Southwest University Adult Lifespan Dataset; F, female; M, male; FD, frame-wise displacement.

**Table S2.** Resting-state fMRI parameters for three datasets

| **Parameters** | **Discovery** | **CNP** | **SALD** |
| --- | --- | --- | --- |
| Scanner | 3.0T General Electric Discovery MR750w | 3.0T Siemens Trio | 3.0T Siemens Trio |
| Sequence | GRE-SS-EPI | T2^*^-weighted EPI | GRE-EPI |
| TR (ms) | 2,000 | 2,000 | 2,000 |
| TE (ms) | 30 | 30 | 30 |
| FA (°) | 90 | 90 | 90 |
| FOV (mm^2^) | 220 × 220 | 192 × 192 | 220 × 220 |
| Matrix size | 64 × 64 | 64 × 64 | 64 × 64 |
| Slice thickness (mm) | 3 | 4 | 3 |
| Slice gap (mm) | 1 | - | 1 |
| Slices | 35 | 34 | 32 |
| Time points | 185 | 152 | 242 |

Abbreviations: fMRI, functional magnetic resonance imaging; CNP, Consortium for Neuropsychiatric Phenomics; SALD, Southwest University Adult Lifespan Dataset; GRE, gradient echo; SS, single shot; EPI, echo planar imaging; TR, repetition time; TE, echo time; FA, flip angle; FOV, field of view.

**Table S3. Demographic information of the six adult donors in the AHBA**

| **Donor** | **Age (years)** | **Gender** | **Ethnicity** | **Hemisphere** | **Post-mortem interval (h)** |
| --- | --- | --- | --- | --- | --- |
| H0351.2001 | 24 | Male | African American | Both | 23 |
| H0351.2002 | 39 | Male | African American | Both | 10 |
| H0351.1009 | 57 | Male | Caucasian | Left | 25.5 |
| H0351.1012 | 31 | Male | Caucasian | Left | 17.5 |
| H0351.1015 | 49 | Female | Hispanic | Left | 30 |
| H0351.1016 | 55 | Male | Caucasian | Left | 18 |

Abbreviations: AHBA, Allen Human Brain Atlas.

**Table S4.** Numbers of the strongly contributing genes identified in the two validation datasets and overlaps with those identified in the discovery dataset

| **Dataset** | **Strongly contributing gene set** | **Gene number** | **Overlap**  **genes** | **Overlap ratio** |
| --- | --- | --- | --- | --- |
| CNP | PLS+ genes | 562 | 459 | 92.35% |
|  | PLS- genes | 692 | 643 | 85.05% |
| SALD | PLS+ genes | 536 | 466 | 93.76% |
|  | PLS- genes | 718 | 682 | 90.21% |

Abbreviations: CNP, Consortium for Neuropsychiatric Phenomics; SALD, Southwest University Adult Lifespan Dataset; PLS, partial least squares.

**Supplementary figures**

**
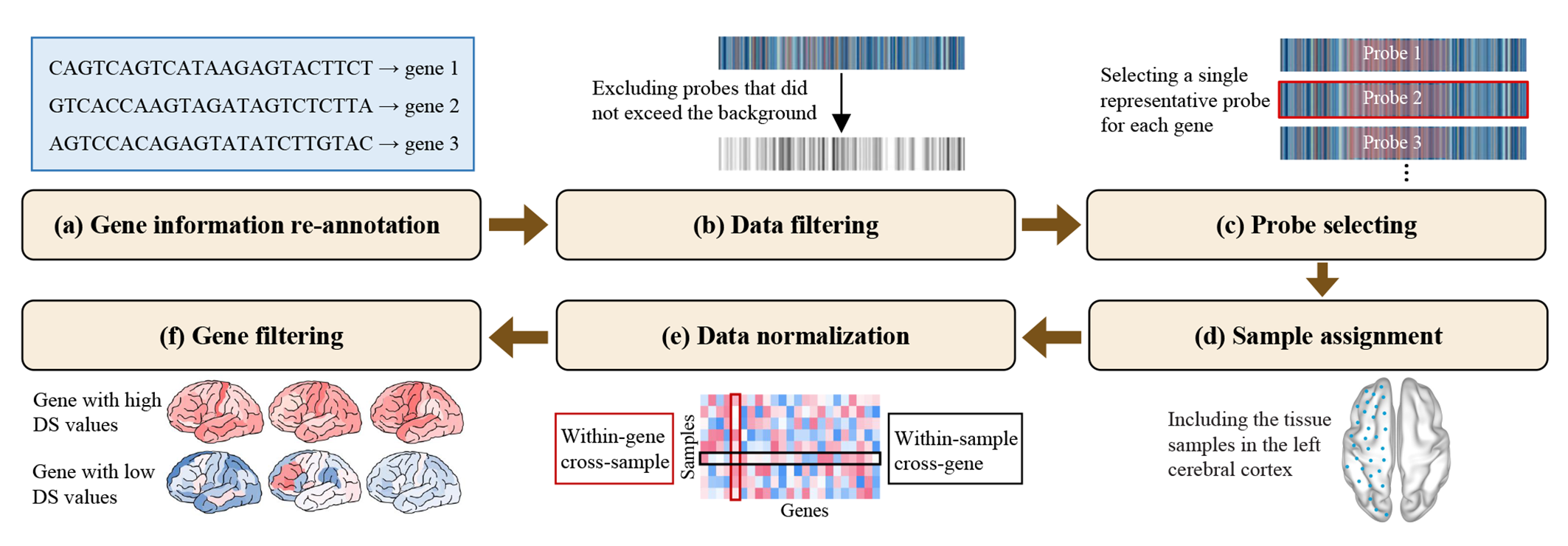
**

**Figure S1.** Schematic of a workflow for processing brain gene expression data. (a) Probe-to-gene annotations were updated based on the latest available information. (b) Probes that did not exceed the background noise in at least 50% of samples across all donors were excluded. (c) For genes indexed by multiple probes, a representative probe for a gene was selected based on the highest correlation to the RNA-seq data. (d) Sample assignment was performed and only tissue samples in the left cerebral cortex were included. (e) Both within-sample cross-gene and within-gene cross-sample normalizations were performed. (f) The 50% of the highest DS genes were chosen. Abbreviations: DS, differential stability.

**
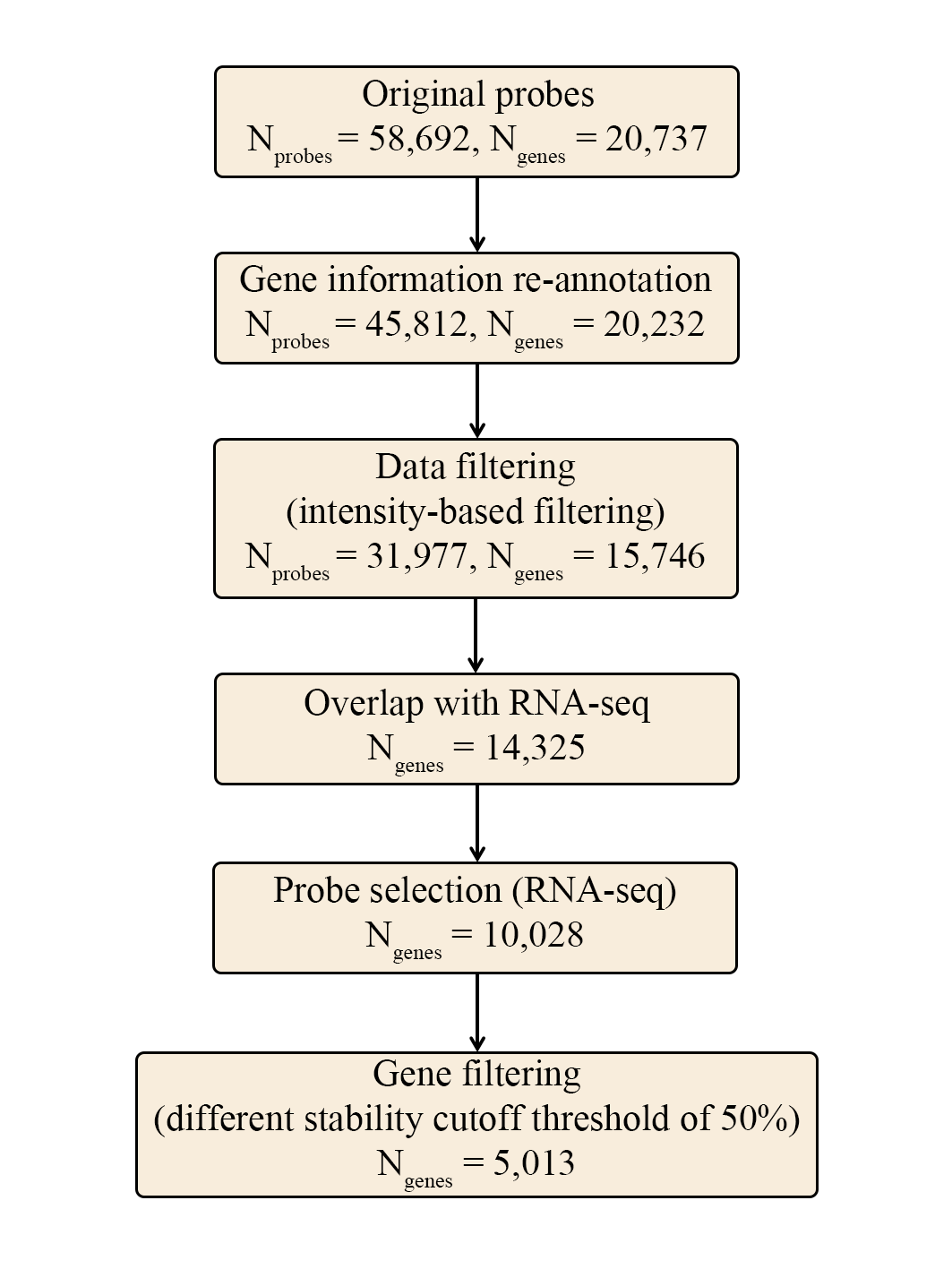
**

**Figure S2.** The remaining probes and genes at each processing step. Abbreviations: RNA, ribonucleic acid.

**
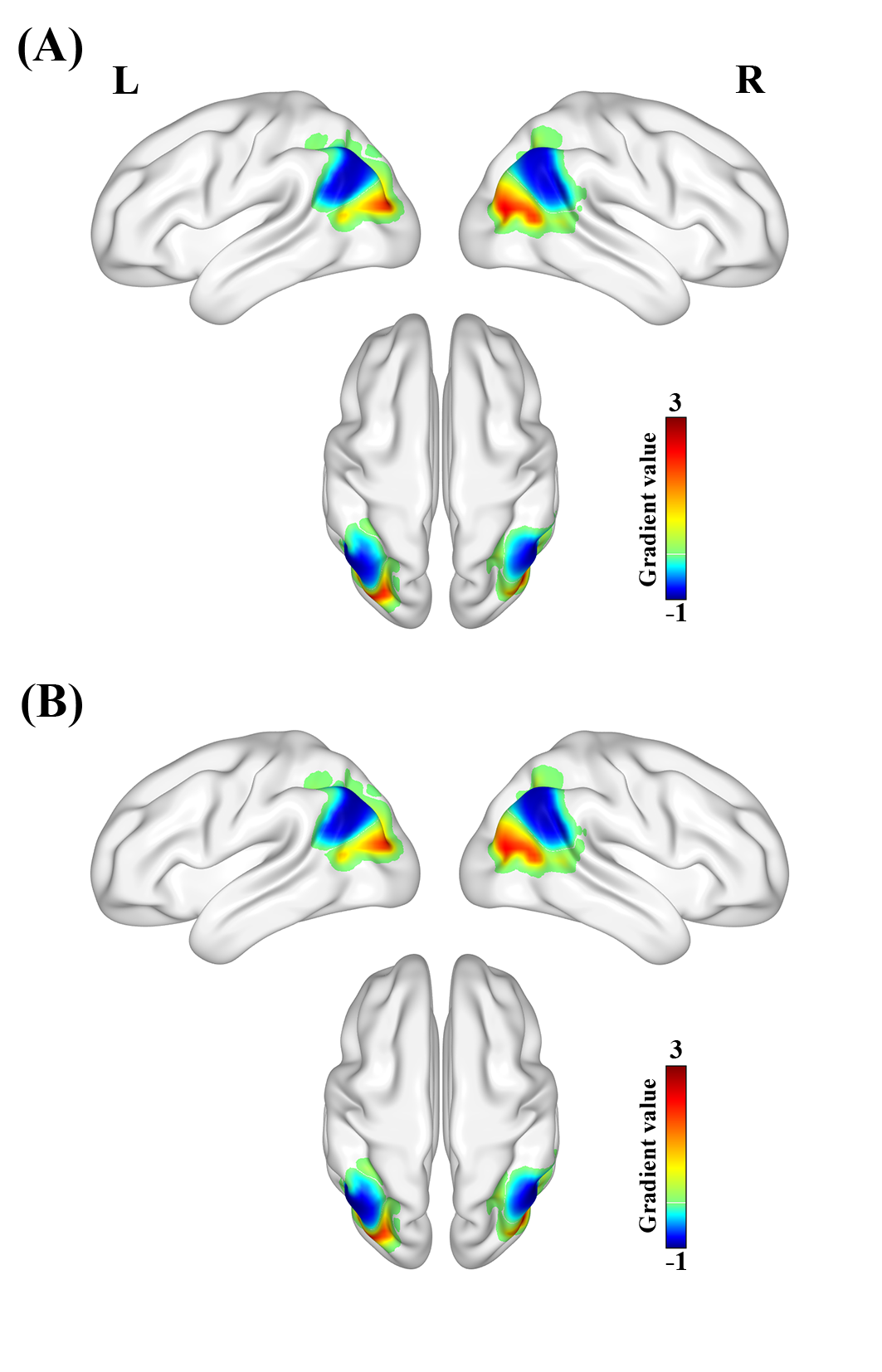
**

**Figure S3.** Topography of the dominant gradient derived from two other rsFC matrix thresholds of top 20% (A) and 30% (B). Abbreviations: L, left; R, right; rsFC, resting-state functional connectivity.

**
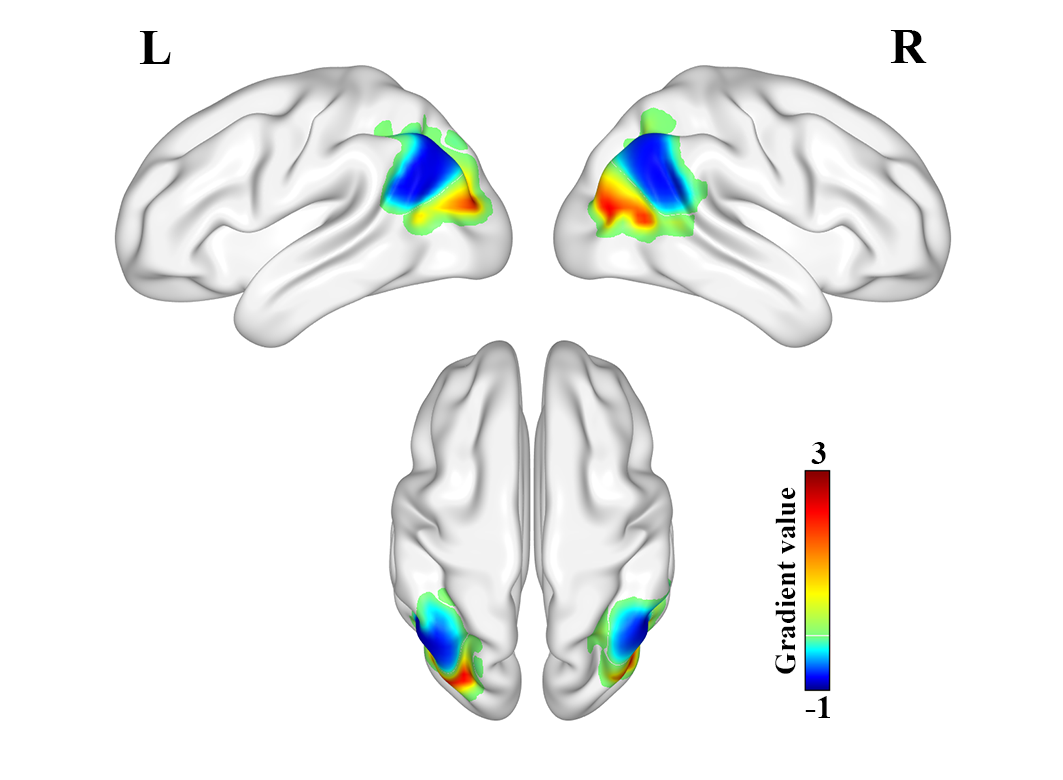
**

**Figure S4.** Topography of the dominant gradient based on BOLD data with GSR. Abbreviations: L, left; R, right; BOLD, blood-oxygen-level-dependent; GSR, global signal regression.


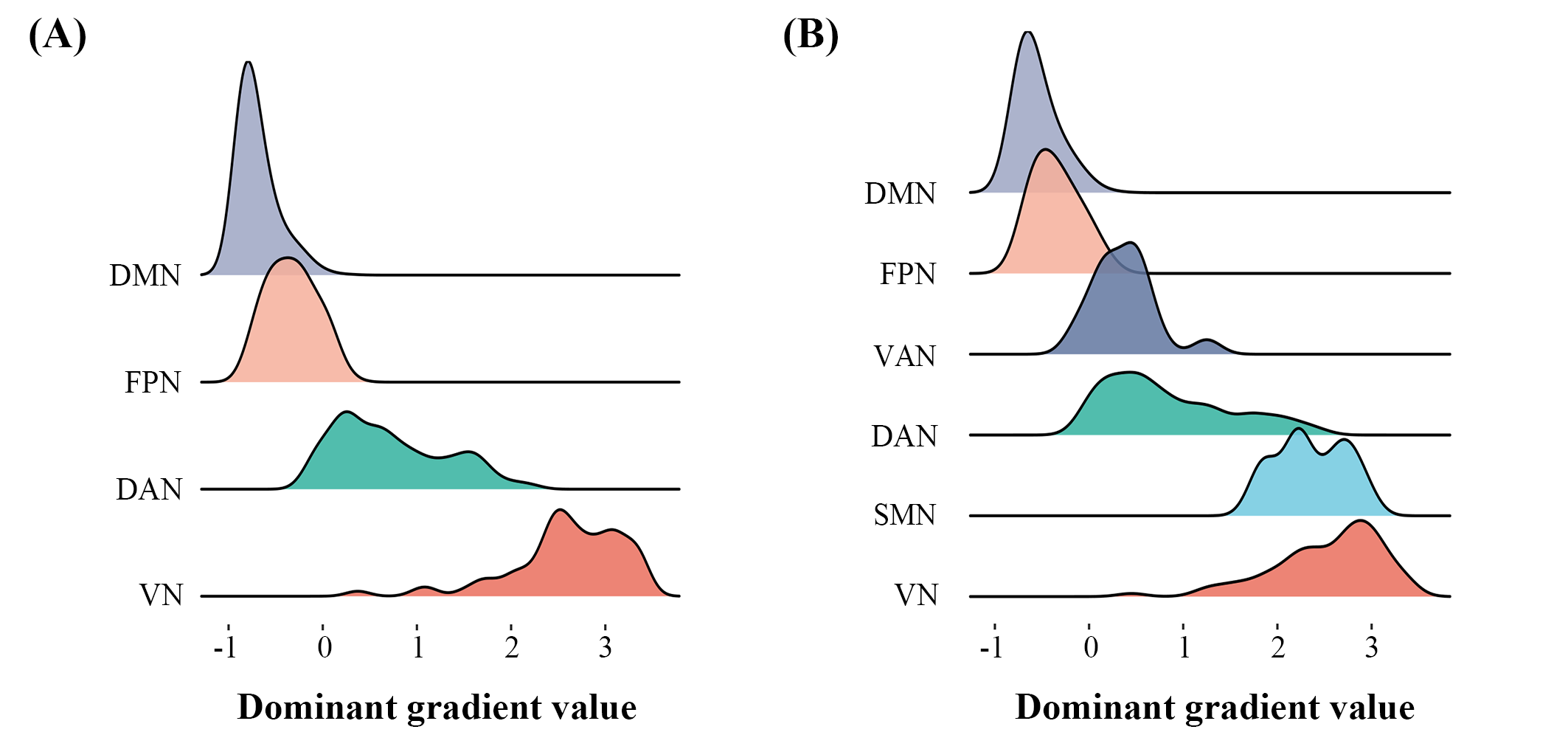


**Figure S5.** Distributions of the AG functional subdivisions corresponding to the canonical functional networks along the dominant gradient for the left (A) and right (B) AG. Some functional subdivisions (i.e., SMN and VAN for the left AG, and LN for the bilateral AG) were not found. Abbreviations: DMN, default mode network; FPN, frontoparietal network; VAN, ventral attention network; DAN, dorsal attention network; SMN, sensorimotor network; VN, visual network; LN, limbic network; AG, angular gyrus.


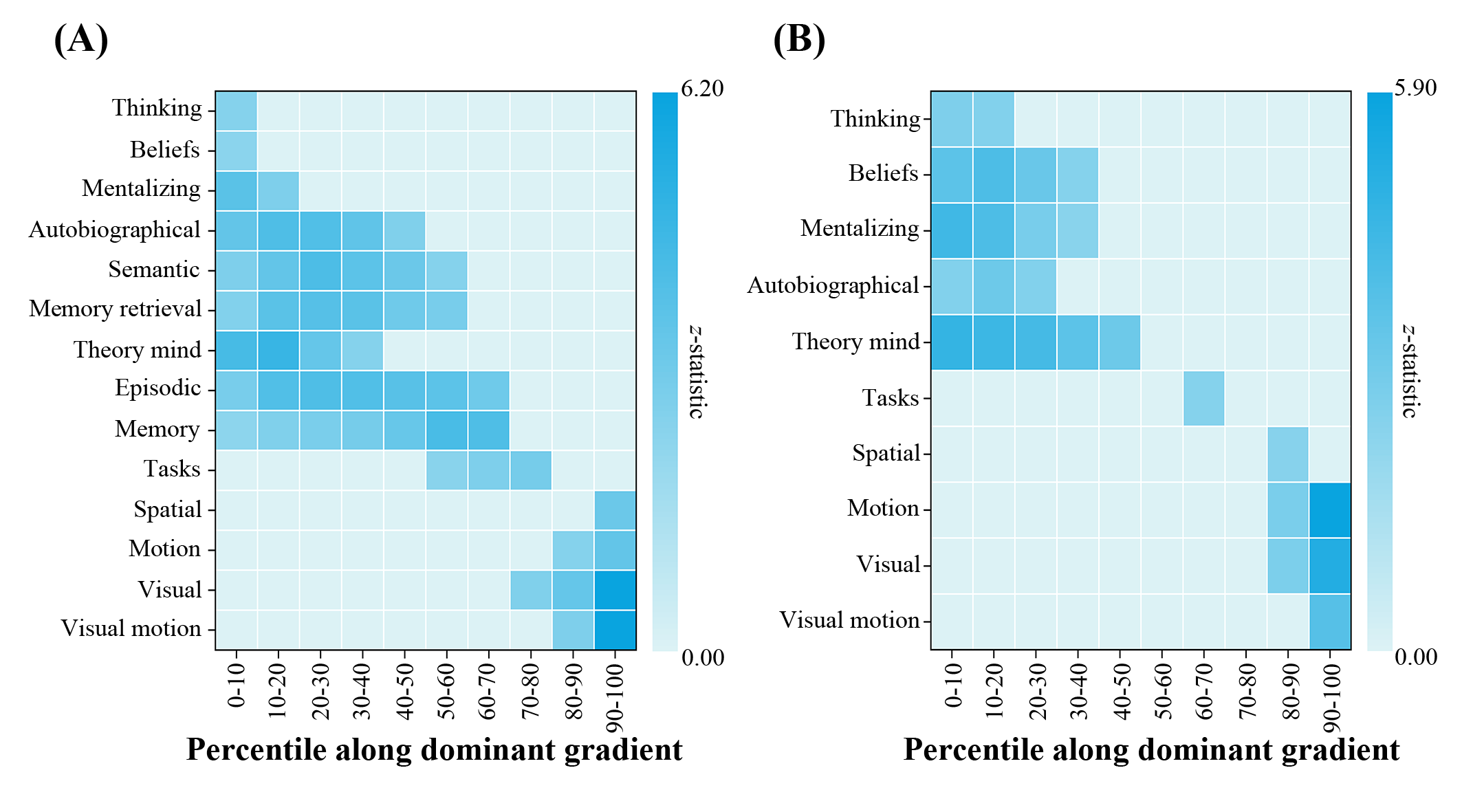


**Figure S6.** Associations between the dominant gradient and behavioral terms from the NeuroSynth for the left (A) and right (B) AG. Abbreviations: AG, angular gyrus.


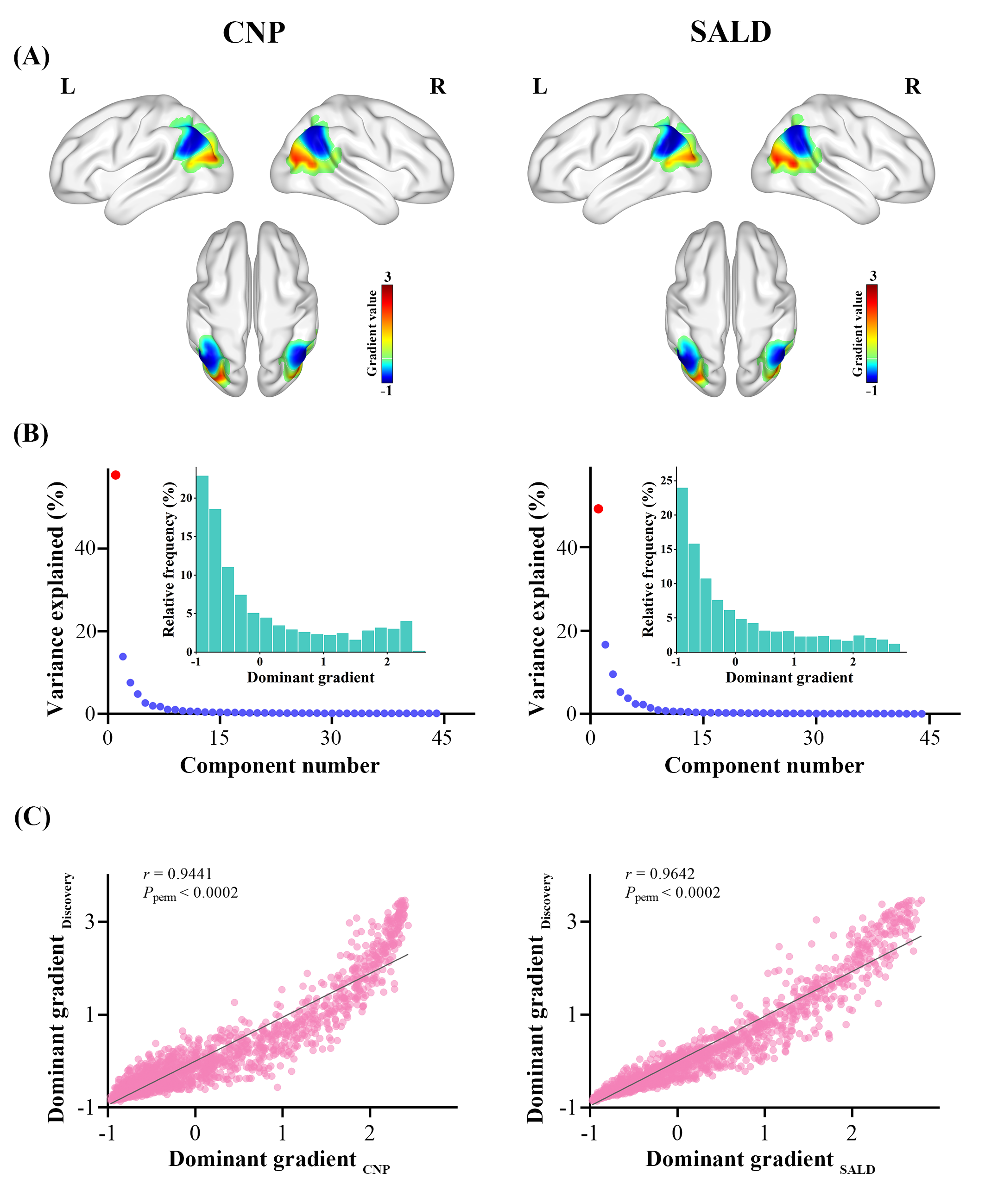


**Figure S7.** Functional gradients of the AG in CNP and SALD datasets. (A) Topography of the dominant gradient. (B) Variance explained by the functional gradients. Inserted histograms demonstrated the distributions of the dominant gradient values of voxels within the AG. (C) Cross-voxel spatial correlations between the dominant gradient values of the discovery and validation datasets. Abbreviations: CNP, Consortium for Neuropsychiatric Phenomics; SALD, Southwest University Adult Lifespan Dataset; L, left; R, right; AG, angular gyrus.


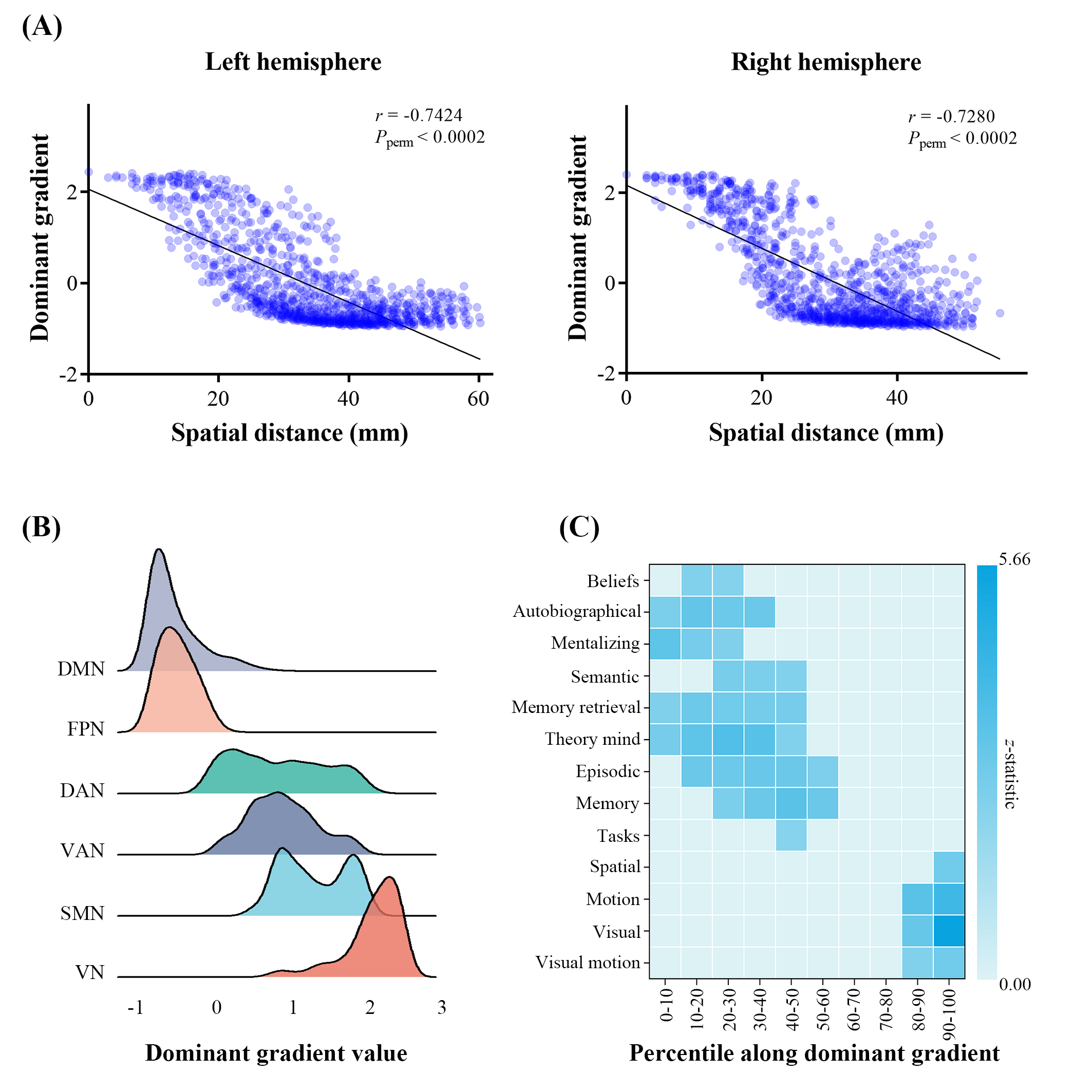


**Figure S8.** Relations of the AG dominant functional gradient to intrinsic geometry, functional networks, and behavioral domains in CNP dataset. (A) Associations between the dominant gradient and spatial distance from the peak voxel in both hemispheres. (B) Distribution of the AG functional subdivisions corresponding to the canonical functional networks along the dominant gradient. The functional subdivision corresponding to the limbic network was not found. (C) Associations between the dominant gradient and behavioral terms from the NeuroSynth. Abbreviations: DMN, default mode network; FPN, frontoparietal network; DAN, dorsal attention network; VAN, ventral attention network; SMN, sensorimotor network; VN, visual network; AG, angular gyrus; CNP, Consortium for Neuropsychiatric Phenomics**.**

**
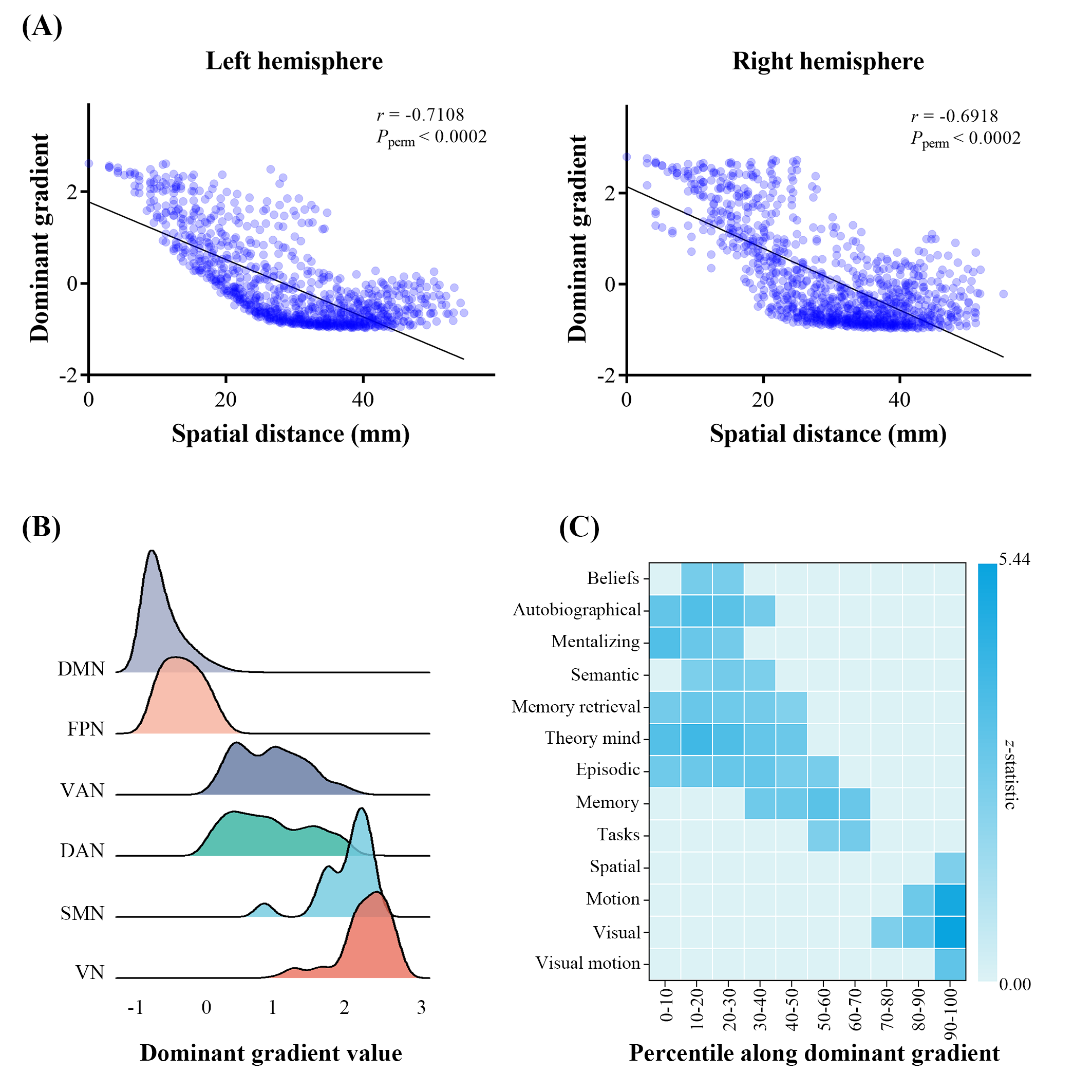
**

**Figure S9.** Relations of the AG dominant functional gradient to intrinsic geometry, functional networks, and behavioral domains in SALD dataset. (A) Associations between the dominant gradient and spatial distance from the peak voxel in both hemispheres. (B) Distribution of the AG functional subdivisions corresponding to the canonical functional networks along the dominant gradient. The functional subdivision corresponding to the limbic network was not found. (C) Associations between the dominant gradient and behavioral terms from the NeuroSynth. Abbreviations: DMN, default mode network; FPN, frontoparietal network; VAN, ventral attention network; DAN, dorsal attention network; SMN, sensorimotor network; VN, visual network; AG, angular gyrus; SALD, Southwest University Adult Lifespan Dataset.


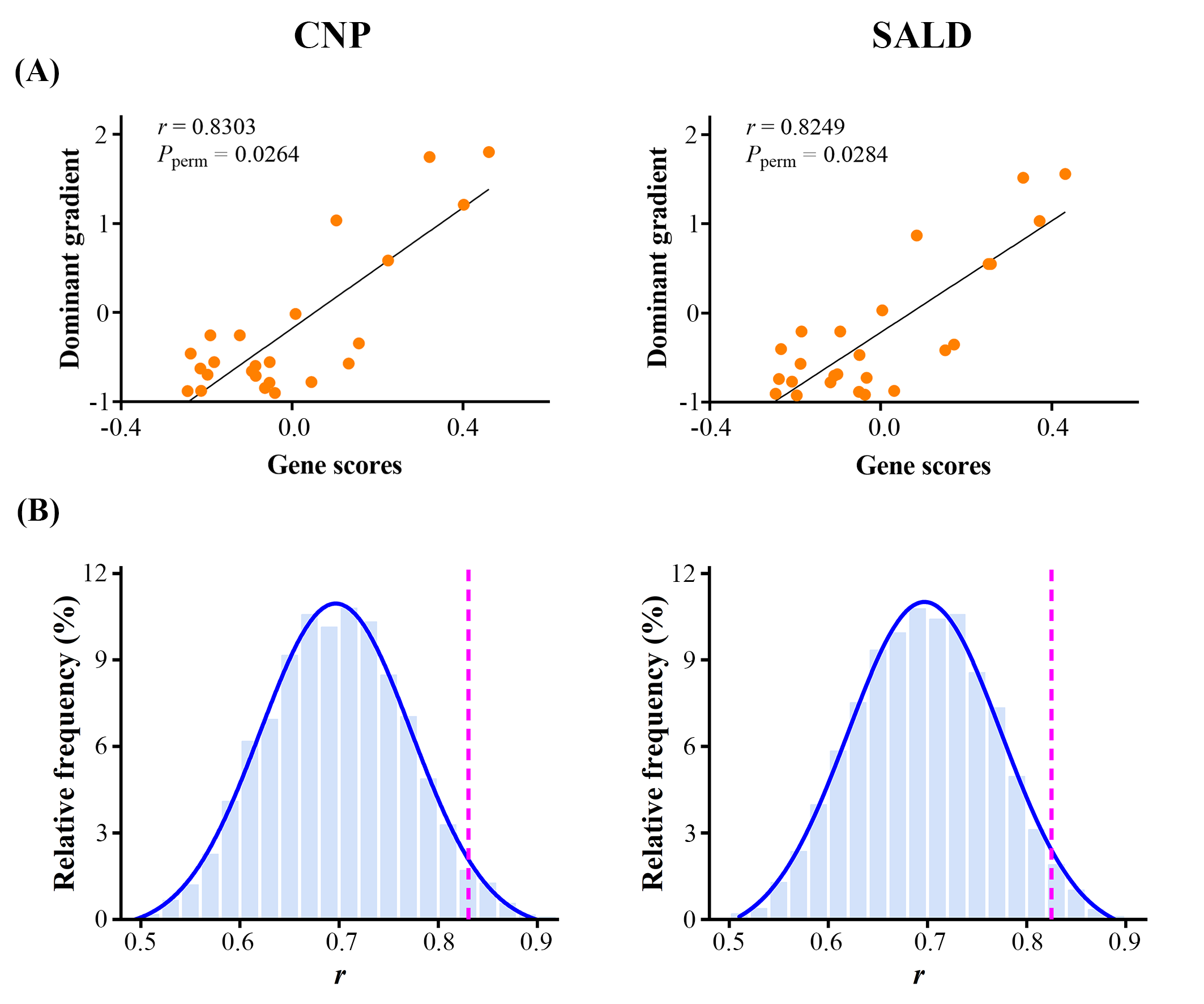


**Figure S10.** Gene expression and the AG dominant functional gradient in CNP and SALD datasets. (A) Scatterplots of gene scores (identified by PLS regression) versus the dominant gradient with each point representing a tissue sample within the AG. (B) Histograms of permutation distributions showing that the correlations (dotted lines) between gene scores and the dominant gradient were significantly greater than expected by chance. Abbreviations: CNP, Consortium for Neuropsychiatric Phenomics; SALD, Southwest University Adult Lifespan Dataset; AG, angular gyrus; PLS, partial least squares.
