# Supplementary material for: Functional hierarchy of the angular gyrus and its underlying genetic architecture
Source: Hum Brain Mapp. 2023 Feb 28;44(7):2815–28. doi: 10.1002/hbm.26247 (PMC10089092; doi:10.1002/hbm.26247)
Supplement: Supplementary file 2 — FILE S1. Genes and gene loadings [file HBM-44-2815-s004.pdf]

Genes and gene loadings		
**Entrez ID**	**Gene name**	**PLS1 loading**
254065	BRWD3	0.7880
4750	NEK1	0.7798
56666	PANX2	0.7613
118987	PDZD8	0.7552
785	CACNB4	0.7387
5863	RGL2	0.7195
79750	ZNF385D	0.7174
165918	RNF168	0.7018
80010	RMI1	0.6996
6324	SCN1B	0.6963
387119	CEP85L	0.6900
22987	SV2C	0.6895
55692	LUC7L	0.6869
468	ATF4	0.6771
51660	MPC1	0.6748
54949	SDHAF2	0.6737
98	ACYP2	0.6722
149951	COMMD7	0.6661
10884	MRPS30	0.6612
4644	MYO5A	0.6574
29997	NOP53	0.6549
10809	STARD10	0.6516
478	ATP1A3	0.6508
7581	ZNF33A	0.6494
347731	LRRTM3	0.6487
51341	ZBTB7A	0.6486
2035	EPB41	0.6400
647121	EMBP1	0.6388
6103	RPGR	0.6370
5899	RALB	0.6338
100287171	WASHC1	0.6337
85009	MGC16025	0.6324
8848	TSC22D1	0.6254
25830	SULT4A1	0.6242
610	HCN2	0.6210
27328	PCDH11X	0.6201
84124	ZNF394	0.6181
22895	RPH3A	0.6177
11228	RASSF8	0.6117
9583	ENTPD4	0.6086
8932	MBD2	0.6081
5578	PRKCA	0.6066
23207	PLEKHM2	0.6062
4741	NEFM	0.6060
25861	WHRN	0.6055
3902	LAG3	0.6037
64781	CERK	0.6020
6185	RPN2	0.6011
9811	CTIF	0.6006

7351	UCP2	0.6005
84890	ADO	0.5992
22890	ZBTB1	0.5990
440823	MIAT	0.5982
10014	HDAC5	0.5944
7110	TMF1	0.5915
4213	MEIS3P1	0.5866
55745	AP5M1	0.5827
7411	VBP1	0.5825
400954	EML6	0.5763
85015	USP45	0.5749
10815	CPLX1	0.5744
3338	DNAJC4	0.5719
5562	PRKAA1	0.5703
29992	PILRA	0.5690
3737	KCNA2	0.5680
7695	ZNF136	0.5674
56927	GPR108	0.5619
221336	BEND6	0.5611
16	AARS	0.5603
23032	USP33	0.5599
6002	RGS12	0.5585
57639	CCDC146	0.5577
51028	VPS36	0.5568
3798	KIF5A	0.5543
6812	STXBP1	0.5478
79956	ERMP1	0.5473
10778	ZNF271P	0.5448
142678	MIB2	0.5434
83737	ITCH	0.5423
375567	VWC2	0.5420
203	AK1	0.5403
4430	MYO1B	0.5393
11342	RNF13	0.5391
112724	RDH13	0.5380
5134	PDCD2	0.5369
100132341	CLUHP3	0.5363
343990	KIAA1211L	0.5333
10645	CAMKK2	0.5323
23345	SYNE1	0.5317
284252	KCTD1	0.5313
79675	FASTKD1	0.5305
2273	FHL1	0.5298
116151	FAM210B	0.5298
6093	ROCK1	0.5286
2051	EPHB6	0.5270
57528	KCTD16	0.5269
5090	PBX3	0.5252
56907	SPIRE1	0.5242
2023	ENO1	0.5233
2778	GNAS	0.5222
26578	OSTF1	0.5195
79913	ACTR5	0.5184
158135	TTLL11	0.5175
51171	HSD17B14	0.5173

7074	TIAM1	0.5171
114791	TUBGCP5	0.5166
26353	HSPB8	0.5143
55806	HR	0.5136
5455	POU3F3	0.5136
26953	RANBP6	0.5128
51360	MBTPS2	0.5122
3106	HLA-B	0.5116
59284	CACNG7	0.5111
5315	PKM	0.5098
286205	SCAI	0.5085
4170	MCL1	0.5054
9479	MAPK8IP1	0.5049
1909	EDNRA	0.5048
3572	IL6ST	0.5047
57799	RAB40C	0.5038
4061	LY6E	0.5036
22848	AAK1	0.5031
9969	MED13	0.5026
100130950	LOC10013095(	0.5024
84818	IL17RC	0.4985
26051	PPP1R16B	0.4985
7625	ZNF74	0.4981
4839	NOP2	0.4980
3717	JAK2	0.4973
114804	RNF157	0.4954
29088	MRPL15	0.4946
83482	SCRT1	0.4927
26268	FBXO9	0.4916
9314	KLF4	0.4916
54919	DNAAF5	0.4915
51678	MPP6	0.4909
349152	DPY19L2P2	0.4893
83988	NCALD	0.4892
440279	UNC13C	0.4891
9886	RHOBTB1	0.4886
8672	EIF4G3	0.4872
491	ATP2B2	0.4871
10769	PLK2	0.4854
91404	SESTD1	0.4853
5274	SERPINI1	0.4845
9253	NUMBL	0.4836
91624	NEXN	0.4820
6124	RPL4	0.4815
2288	FKBP4	0.4811
253639	ZNF620	0.4798
57820	CCNB1IP1	0.4787
134510	UBLCP1	0.4776
51555	PEX5L	0.4768
9482	STX8	0.4764
55695	NSUN5	0.4763
2743	GLRB	0.4761
53615	MBD3	0.4758
51719	CAB39	0.4742
1124	CHN2	0.4742

79970	ZNF767P	0.4738
9265	CYTH3	0.4738
79658	ARHGAP10	0.4737
80124	VCPIP1	0.4737
2554	GABRA1	0.4723
57198	ATP8B2	0.4722
140597	TCEAL2	0.4717
167691	LCA5	0.4709
2555	GABRA2	0.4708
57380	MRS2	0.4694
127396	ZNF684	0.4694
4205	MEF2A	0.4693
162494	RHBDL3	0.4680
3123	HLA-DRB1	0.4673
84256	FLYWCH1	0.4669
64398	MPP5	0.4660
490	ATP2B1	0.4652
9205	ZMYM5	0.4642
3708	ITPR1	0.4629
6389	SDHA	0.4620
147727	ILF3-AS1	0.4618
9732	DOCK4	0.4607
9885	OSBPL2	0.4601
9263	STK17A	0.4582
5557	PRIM1	0.4582
1628	DBP	0.4580
10385	BTN2A2	0.4578
23305	ACSL6	0.4577
79365	BHLHE41	0.4576
889	KRIT1	0.4564
6305	SBF1	0.4551
5999	RGS4	0.4551
6666	SOX12	0.4548
5179	PENK	0.4540
10314	LANCL1	0.4538
55529	PIP4P2	0.4536
348801	LNP1	0.4535
1959	EGR2	0.4534
57489	ODF2L	0.4530
340204	CLPSL1	0.4521
7673	ZNF222	0.4514
51522	TMEM14C	0.4501
8028	MLLT10	0.4498
6451	SH3BGRL	0.4482
8775	NAPA	0.4463
4782	NFIC	0.4462
112755	STX1B	0.4461
4675	NAP1L3	0.4458
127833	SYT2	0.4453
55582	KIF27	0.4447
28966	SNX24	0.4443
57559	STAMBPL1	0.4442
57821	CCDC181	0.4423
6843	VAMP1	0.4422
22995	CEP152	0.4418

3423	IDS	0.4406
22891	ZNF365	0.4403
64118	DUS1L	0.4392
9478	CABP1	0.4391
5187	PER1	0.4390
23078	VWA8	0.4389
55203	LGI2	0.4369
4092	SMAD7	0.4367
88	ACTN2	0.4354
412	STS	0.4350
1495	CTNNA1	0.4348
9177	HTR3B	0.4342
51710	ZNF44	0.4342
9815	GIT2	0.4335
654817	NCF1C	0.4316
54522	ANKRD16	0.4310
23136	EPB41L3	0.4305
400569	MED11	0.4298
10983	CCNI	0.4275
23623	RUSC1	0.4266
83719	YPEL3	0.4263
80820	EEPD1	0.4261
3624	INHBA	0.4259
54438	GFOD1	0.4257
9749	PHACTR2	0.4249
3911	LAMA5	0.4243
283417	DPY19L2	0.4237
51201	ZDHHC2	0.4228
55125	CEP192	0.4228
401145	CCSER1	0.4221
2135	EXTL2	0.4215
130399	ACVR1C	0.4212
120892	LRRK2	0.4209
29951	PDZRN4	0.4206
56660	KCNK12	0.4206
9196	KCNAB3	0.4205
2353	FOS	0.4197
60484	HAPLN2	0.4185
149420	PDIK1L	0.4183
6404	SELPLG	0.4168
57646	USP28	0.4150
79572	ATP13A3	0.4149
83853	ROPN1L	0.4144
2971	GTF3A	0.4140
34	ACADM	0.4136
25787	DGCR9	0.4130
1012	CDH13	0.4103
3696	ITGB8	0.4097
23764	MAFF	0.4096
80854	SETD7	0.4093
4715	NDUFB9	0.4090
29899	GPSM2	0.4088
10422	UBAC1	0.4084
8379	MAD1L1	0.4080
131076	CCDC58	0.4070

9699	RIMS2	0.4066
22876	INPP5F	0.4058
60487	TRMT11	0.4050
3752	KCND3	0.4041
27109	ATP5S	0.4036
9651	PLCH2	0.4032
9382	COG1	0.4028
80817	CEP44	0.4025
123606	NIPA1	0.4022
7791	ZYX	0.4017
149297	FAM78B	0.4009
55632	G2E3	0.4004
51100	SH3GLB1	0.4003
9246	UBE2L6	0.3996
26225	ARL5A	0.3996
219	ALDH1B1	0.3975
60485	SAV1	0.3975
286	ANK1	0.3971
176	ACAN	0.3970
83875	BCO2	0.3970
60313	GPBP1L1	0.3968
114815	SORCS1	0.3965
3748	KCNC3	0.3963
2585	GALK2	0.3959
51389	RWDD1	0.3957
10052	GJC1	0.3954
22889	KHDC4	0.3948
1958	EGR1	0.3944
54331	GNG2	0.3942
84107	ZIC4	0.3939
85459	CEP295	0.3937
94032	CAMK2N2	0.3934
1278	COL1A2	0.3931
808	CALM3	0.3922
9337	CNOT8	0.3916
57469	PNMA8B	0.3912
9258	MFHAS1	0.3911
23154	NCDN	0.3910
51574	LARP7	0.3909
23237	ARC	0.3903
91179	SCARF2	0.3886
292	SLC25A5	0.3885
4299	AFF1	0.3881
5456	POU3F4	0.3881
6000	RGS7	0.3879
57181	SLC39A10	0.3878
54739	XAF1	0.3868
63893	UBE2O	0.3865
3756	KCNH1	0.3862
442213	PTCHD4	0.3839
2857	GPR34	0.3837
6845	VAMP7	0.3833
79912	PYROXD1	0.3827
57451	TENM2	0.3827
260425	MAGI3	0.3821

9372	ZFYVE9	0.3819
51517	NCKIPSD	0.3818
64981	MRPL34	0.3797
644242	LINC00622	0.3797
5594	MAPK1	0.3790
10512	SEMA3C	0.3784
58486	ZBED5	0.3781
347730	LRRTM1	0.3779
8237	USP11	0.3778
5476	CTSA	0.3772
2621	GAS6	0.3772
10010	TANK	0.3771
84144	SYDE2	0.3770
9783	RIMS3	0.3763
816	CAMK2B	0.3761
2563	GABRD	0.3759
25870	SUMF2	0.3754
30850	CDR2L	0.3751
4739	NEDD9	0.3750
2819	GPD1	0.3746
2901	GRIK5	0.3746
9131	AIFM1	0.3745
2675	GFRA2	0.3744
10846	PDE10A	0.3729
2042	EPHA3	0.3729
2903	GRIN2A	0.3725
64225	ATL2	0.3723
51174	TUBD1	0.3719
54469	ZFAND6	0.3716
28987	NOB1	0.3711
27115	PDE7B	0.3706
5295	PIK3R1	0.3701
9581	PREPL	0.3691
51377	UCHL5	0.3689
1122	CHML	0.3688
23316	CUX2	0.3686
1040	CDS1	0.3673
84971	ATG4D	0.3669
2104	ESRRG	0.3663
8821	INPP4B	0.3659
419	ART3	0.3655
89941	RHOT2	0.3651
10781	ZNF266	0.3647
2296	FOXC1	0.3646
3208	HPCA	0.3641
22884	WDR37	0.3638
55644	OSGEP	0.3630
9648	GCC2	0.3623
9261	MAPKAPK2	0.3610
55904	KMT2E	0.3609
57142	RTN4	0.3604
57162	PELI1	0.3601
9957	HS3ST1	0.3601
51635	DHRS7	0.3598
171177	RHOV	0.3586

83723	FAM57B	0.3580
55161	TMEM33	0.3579
84572	GNPTG	0.3577
5718	PSMD12	0.3577
54529	ASNSD1	0.3576
55219	MACO1	0.3567
79158	GNPTAB	0.3560
2561	GABRB2	0.3557
6667	SP1	0.3552
57221	ARFGEF3	0.3551
5142	PDE4B	0.3549
4641	MYO1C	0.3541
27319	BHLHE22	0.3541
2826	CCR10	0.3539
8536	CAMK1	0.3534
79001	VKORC1	0.3534
23762	OSBP2	0.3528
3241	HPCAL1	0.3524
718	C3	0.3517
622	BDH1	0.3514
84148	KAT8	0.3511
4703	NEB	0.3508
145957	NRG4	0.3502
567	B2M	0.3491
6092	ROBO2	0.3490
55088	CCDC186	0.3489
27165	GLS2	0.3489
122618	PLD4	0.3483
5264	PHYH	0.3481
5889	RAD51C	0.3470
883	KYAT1	0.3468
4824	NKX3-1	0.3466
81539	SLC38A1	0.3466
51667	NUB1	0.3463
5150	PDE7A	0.3462
342667	STAC2	0.3462
91526	ANKRD44	0.3459
653238	GTF2H2B	0.3457
7942	TFEB	0.3453
7832	BTG2	0.3453
1438	CSF2RA	0.3450
6482	ST3GAL1	0.3448
203190	LGI3	0.3446
5332	PLCB4	0.3433
7431	VIM	0.3430
29	ABR	0.3422
55578	SUPT20H	0.3421
3611	ILK	0.3416
55450	CAMK2N1	0.3414
84188	FAR1	0.3413
4085	MAD2L1	0.3412
63941	NECAB3	0.3411
5325	PLAGL1	0.3409
79176	FBXL15	0.3405
10494	STK25	0.3402

64332	NFKBIZ	0.3402
51506	UFC1	0.3395
7392	USF2	0.3388
1392	CRH	0.3388
57414	RHBDD2	0.3388
9975	NR1D2	0.3382
3716	JAK1	0.3380
374291	NDUFS7	0.3376
80031	SEMA6D	0.3370
93556	EGFEM1P	0.3370
23522	KAT6B	0.3362
30811	HUNK	0.3361
5564	PRKAB1	0.3354
10319	LAMC3	0.3352
8537	BCAS1	0.3349
55206	SBNO1	0.3349
27289	RND1	0.3347
126393	HSPB6	0.3332
4360	MRC1	0.3332
284348	LYPD5	0.3325
3055	HCK	0.3324
284069	FAM171A2	0.3323
10140	TOB1	0.3321
50865	HEBP1	0.3321
10900	RUNDC3A	0.3315
10332	CLEC4M	0.3314
58500	ZNF250	0.3310
387856	CCDC184	0.3304
5412	UBL3	0.3302
56204	FAM214A	0.3300
158696	LINC00889	0.3296
10150	MBNL2	0.3293
4744	NEFH	0.3293
56143	PCDHA5	0.3291
27230	SERP1	0.3284
214	ALCAM	0.3277
7552	ZNF711	0.3272
200058	FLJ23867	0.3272
9619	ABCG1	0.3263
8482	SEMA7A	0.3262
84444	DOT1L	0.3258
147463	ANKRD29	0.3257
4130	MAP1A	0.3256
6876	TAGLN	0.3255
10964	IFI44L	0.3255
221662	RBM24	0.3249
7305	TYROBP	0.3249
54813	KLHL28	0.3246
158219	TTC39B	0.3245
29894	CPSF1	0.3238
55729	ATF7IP	0.3238
9900	SV2A	0.3236
7390	UROS	0.3231
768096	HAR1A	0.3228
636	BICD1	0.3226

54435	HCG4	0.3224
51167	CYB5R4	0.3222
90990	KIFC2	0.3218
84460	ZMAT1	0.3210
51306	FAM13B	0.3201
9412	MED21	0.3193
10617	STAMBP	0.3188
8243	SMC1A	0.3185
56978	PRDM8	0.3184
22796	COG2	0.3181
7805	LAPTM5	0.3178
54414	SIAE	0.3177
23015	GOLGA8A	0.3175
29952	DPP7	0.3171
6603	SMARCD2	0.3163
2119	ETV5	0.3162
85378	TUBGCP6	0.3158
130733	TMEM178A	0.3158
1393	CRHBP	0.3148
83259	PCDH11Y	0.3145
23415	KCNH4	0.3143
57644	MYH7B	0.3142
9223	MAGI1	0.3136
2257	FGF12	0.3135
1663	DDX11	0.3134
90952	ESAM	0.3132
50488	MINK1	0.3130
7059	THBS3	0.3130
51053	GMNN	0.3128
901	CCNG2	0.3125
79740	ZBBX	0.3118
3736	KCNA1	0.3115
3483	IGFALS	0.3113
92304	SCGB3A1	0.3113
100126793	GHRLOS	0.3112
64805	P2RY12	0.3111
359948	IRF2BP2	0.3109
23416	KCNH3	0.3107
51750	RTEL1	0.3104
114088	TRIM9	0.3101
5791	PTPRE	0.3101
23590	PDSS1	0.3099
9948	WDR1	0.3096
9572	NR1D1	0.3095
3115	HLA-DPB1	0.3093
9522	SCAMP1	0.3092
5256	PHKA2	0.3089
10814	CPLX2	0.3089
1821	DRP2	0.3086
8745	ADAM23	0.3085
11118	BTN3A2	0.3081
22853	LMTK2	0.3068
6182	MRPL12	0.3056
11337	GABARAP	0.3055
51315	KRCC1	0.3048

81786	TRIM7	0.3048
3274	HRH2	0.3038
326	AIRE	0.3038
84501	SPIRE2	0.3024
64943	NT5DC2	0.3017
10580	SORBS1	0.3012
59353	TMEM35A	0.3007
5095	PCCA	0.3006
64837	KLC2	0.3006
10243	GPHN	0.3001
10347	ABCA7	0.2999
8814	CDKL1	0.2999
114876	OSBPL1A	0.2998
23286	WWC1	0.2996
144165	PRICKLE1	0.2993
10622	POLR3G	0.2987
4826	NNAT	0.2985
83698	CALN1	0.2980
283455	KSR2	0.2979
125488	TTC39C	0.2971
23334	SZT2	0.2969
26064	RAI14	0.2967
114803	MYSM1	0.2964
55296	TBC1D19	0.2943
9463	PICK1	0.2937
1113	CHGA	0.2932
1159	CKMT1B	0.2931
65010	SLC26A6	0.2924
8727	CTNNAL1	0.2906
11113	CIT	0.2898
642819	ZNF487	0.2890
282974	STK32C	0.2879
200958	MUC20	0.2879
22847	ZNF507	0.2877
57582	KCNT1	0.2877
5552	SRGN	0.2877
10858	CYP46A1	0.2876
7077	TIMP2	0.2875
4724	NDUFS4	0.2874
54885	TBC1D8B	0.2870
9208	LRRFIP1	0.2868
51319	RSRC1	0.2868
64838	FNDC4	0.2868
6525	SMTN	0.2867
11095	ADAMTS8	0.2866
81550	TDRD3	0.2865
84708	LNX1	0.2865
55279	ZNF654	0.2864
55656	INTS8	0.2863
10497	UNC13B	0.2860
151835	CPNE9	0.2860
43	ACHE	0.2855
54468	MIOS	0.2853
10451	VAV3	0.2852
92949	ADAMTSL1	0.2851

339829	CCDC39	0.2846
2048	EPHB2	0.2845
27012	KCNV1	0.2839
2181	ACSL3	0.2834
112483	SAT2	0.2831
196074	METTL15	0.2823
694	BTG1	0.2821
6518	SLC2A5	0.2819
3134	HLA-F	0.2817
22925	PLA2R1	0.2816
27252	KLHL20	0.2812
7434	VIPR2	0.2807
5454	POU3F2	0.2806
10636	RGS14	0.2803
5530	PPP3CA	0.2801
55577	NAGK	0.2801
29902	FAM216A	0.2799
1436	CSF1R	0.2790
7145	TNS1	0.2789
51754	TMEM8B	0.2776
23285	KIAA1107	0.2773
8604	SLC25A12	0.2765
7473	WNT3	0.2764
59084	ENPP5	0.2758
55023	PHIP	0.2756
23031	MAST3	0.2749
4900	NRGN	0.2748
81671	VMP1	0.2747
23178	PASK	0.2747
113829	SLC35A4	0.2746
55074	OXR1	0.2742
7068	THRB	0.2741
89765	RSPH1	0.2737
10954	PDIA5	0.2735
64421	DCLRE1C	0.2735
8611	PLPP1	0.2734
2783	GNB2	0.2730
6135	RPL11	0.2727
9922	IQSEC1	0.2721
7903	ST8SIA4	0.2721
815	CAMK2A	0.2721
89796	NAV1	0.2720
3895	KTN1	0.2720
3416	IDE	0.2718
5250	SLC25A3	0.2717
2869	GRK5	0.2715
8895	CPNE3	0.2715
3739	KCNA4	0.2711
5501	PPP1CC	0.2709
4306	NR3C2	0.2708
79762	C1orf115	0.2707
65983	GRAMD2B	0.2704
4790	NFKB1	0.2700
1005	CDH7	0.2698
254552	NUDT8	0.2692

55893	ZNF395	0.2690
2844	GPR21	0.2689
10128	LRPPRC	0.2689
7007	TECTA	0.2687
1463	NCAN	0.2682
29780	PARVB	0.2677
6496	SIX3	0.2671
64753	CCDC136	0.2671
23181	DIP2A	0.2668
9813	EFCAB14	0.2667
3133	HLA-E	0.2665
219988	PATL1	0.2661
344901	OSTN	0.2660
1964	EIF1AX	0.2660
1400	CRMP1	0.2652
55799	CACNA2D3	0.2643
9909	DENND4B	0.2642
26523	AGO1	0.2640
54873	PALMD	0.2640
344838	PAQR9	0.2639
2339	FNTA	0.2627
6304	SATB1	0.2626
58496	LY6G5B	0.2624
54470	ARMCX6	0.2623
25946	ZNF385A	0.2622
57464	STRIP2	0.2621
8099	CDK2AP1	0.2618
84253	GARNL3	0.2614
79836	LONRF3	0.2613
6854	SYN2	0.2611
285605	DTWD2	0.2610
79031	PDCL3	0.2607
26053	AUTS2	0.2604
57118	CAMK1D	0.2603
284454	LOC284454	0.2598
81831	NETO2	0.2598
283659	PRTG	0.2596
254559	MIR9-3HG	0.2594
5018	OXA1L	0.2592
55256	ADI1	0.2587
26750	RPS6KC1	0.2577
116984	ARAP2	0.2573
339479	BRINP3	0.2573
79894	ZNF672	0.2572
9672	SDC3	0.2570
23551	RASD2	0.2568
1535	CYBA	0.2563
84876	ORAI1	0.2560
23457	ABCB9	0.2559
51412	ACTL6B	0.2555
51280	GOLM1	0.2548
83999	KREMEN1	0.2547
79083	MLPH	0.2547
79016	DDA1	0.2544
92745	SLC38A5	0.2542

25953	PNKD	0.2541
57501	KIAA1257	0.2533
23101	MCF2L2	0.2532
55657	ZNF692	0.2531
204962	SLC44A5	0.2530
284370	ZNF615	0.2527
29091	STXBP6	0.2527
137970	UNC5D	0.2526
10857	PGRMC1	0.2525
1310	COL19A1	0.2523
84264	HAGHL	0.2521
65110	UPF3A	0.2521
79663	HSPBAP1	0.2521
3800	KIF5C	0.2516
4289	MKLN1	0.2508
4324	MMP15	0.2504
9765	ZFYVE16	0.2504
10867	TSPAN9	0.2497
8404	SPARCL1	0.2496
56892	TCIM	0.2493
57381	RHOJ	0.2491
3991	LIPE	0.2491
116535	MRGPRF	0.2489
145581	LRFN5	0.2482
10675	CSPG5	0.2480
122622	ADSSL1	0.2471
3767	KCNJ11	0.2468
55636	CHD7	0.2465
63934	ZNF667	0.2464
56967	C14orf132	0.2458
84449	ZNF333	0.2456
8825	LIN7A	0.2454
143686	SESN3	0.2452
11216	AKAP10	0.2452
570	BAAT	0.2449
2070	EYA4	0.2443
83937	RASSF4	0.2443
116	ADCYAP1	0.2436
151258	SLC38A11	0.2436
6856	SYPL1	0.2434
84809	CROCCP2	0.2417
132321	C4orf33	0.2411
2944	GSTM1	0.2410
7422	VEGFA	0.2408
10953	TOMM34	0.2405
2634	GBP2	0.2403
7222	TRPC3	0.2401
143684	FAM76B	0.2393
84515	MCM8	0.2392
57035	RSRP1	0.2391
23545	ATP6V0A2	0.2388
1634	DCN	0.2387
55915	LANCL2	0.2386
388272	C16orf87	0.2385
79041	TMEM38A	0.2385

361	AQP4	0.2379
8884	SLC5A6	0.2376
51162	EGFL7	0.2371
3841	KPNA5	0.2366
79944	L2HGDH	0.2366
283131	NEAT1	0.2366
23281	MTUS2	0.2362
1677	DFFB	0.2361
2920	CXCL2	0.2361
29763	PACSIN3	0.2360
481	ATP1B1	0.2354
11248	NXPH3	0.2353
148281	SYT6	0.2352
7150	TOP1	0.2352
27133	KCNH5	0.2349
51332	SPTBN5	0.2343
84561	SLC12A8	0.2343
1601	DAB2	0.2343
1499	CTNNB1	0.2342
56776	FMN2	0.2341
55764	IFT122	0.2339
203228	C9orf72	0.2336
10383	TUBB4B	0.2336
79629	OCEL1	0.2335
6926	TBX3	0.2334
4973	OLR1	0.2333
1431	CS	0.2333
10915	TCERG1	0.2330
55328	RNLS	0.2320
202333	CMYA5	0.2319
5993	RFX5	0.2318
7424	VEGFC	0.2316
545	ATR	0.2307
90864	SPSB3	0.2306
2355	FOSL2	0.2305
55753	OGDHL	0.2301
50636	ANO7	0.2301
57562	CEP126	0.2301
8887	TAX1BP1	0.2296
23294	ANKS1A	0.2293
1326	MAP3K8	0.2292
5063	PAK3	0.2288
64216	TFB2M	0.2287
64168	NECAB1	0.2286
3799	KIF5B	0.2281
1876	E2F6	0.2278
7360	UGP2	0.2264
64149	C17orf75	0.2262
51022	GLRX2	0.2261
284161	GDPD1	0.2260
146395	GSG1L	0.2252
5463	POU6F1	0.2251
55755	CDK5RAP2	0.2251
5334	PLCL1	0.2248
4267	CD99	0.2247

55502	HES6	0.2246
7046	TGFBR1	0.2246
4542	MYO1F	0.2246
9935	MAFB	0.2245
353322	ANKRD37	0.2245
6263	RYR3	0.2239
55294	FBXW7	0.2236
80727	TTYH3	0.2236
7741	ZSCAN26	0.2227
26505	CNNM3	0.2225
57561	ARRDC3	0.2223
2120	ETV6	0.2217
51020	HDDC2	0.2217
219749	ZNF25	0.2216
92017	SNX29	0.2215
10103	TSPAN1	0.2211
286148	DPY19L4	0.2207
55316	RSAD1	0.2206
4093	SMAD9	0.2200
22801	ITGA11	0.2196
137209	ZNF572	0.2191
8546	AP3B1	0.2191
7175	TPR	0.2190
84520	GON7	0.2188
27152	INTU	0.2185
1678	TIMM8A	0.2185
7545	ZIC1	0.2183
9941	EXOG	0.2177
586	BCAT1	0.2174
1645	AKR1C1	0.2174
8209	C21orf33	0.2170
2562	GABRB3	0.2167
26047	CNTNAP2	0.2167
554236	DPY19L2P1	0.2165
51337	THEM6	0.2165
2760	GM2A	0.2162
637	BID	0.2159
9719	ADAMTSL2	0.2156
64418	TMEM168	0.2154
5937	RBMS1	0.2154
79884	MAP9	0.2153
5924	RASGRF2	0.2151
54943	DNAJC28	0.2148
22854	NTNG1	0.2147
3270	HRC	0.2144
9429	ABCG2	0.2140
23616	SH3BP1	0.2139
1346	COX7A1	0.2139
200894	ARL13B	0.2136
51175	TUBE1	0.2135
790	CAD	0.2129
5046	PCSK6	0.2128
26298	EHF	0.2127
4155	MBP	0.2126
85366	MYLK2	0.2123

55215	FANCI	0.2121
3355	HTR1F	0.2119
57710	KIAA1614	0.2118
22913	RALY	0.2113
1414	CRYBB1	0.2113
10369	CACNG2	0.2108
9501	RPH3AL	0.2106
121256	TMEM132D	0.2104
64478	CSMD1	0.2099
4879	NPPB	0.2098
773	CACNA1A	0.2098
80119	PIF1	0.2097
257194	NEGR1	0.2093
10174	SORBS3	0.2090
199731	CADM4	0.2090
26227	PHGDH	0.2090
10718	NRG3	0.2090
6468	FBXW4	0.2079
25804	LSM4	0.2075
113510	HELQ	0.2075
10447	FAM3C	0.2075
8499	PPFIA2	0.2073
81532	MOB2	0.2072
10268	RAMP3	0.2071
7259	TSPYL1	0.2070
11069	RAPGEF4	0.2069
54806	AHI1	0.2066
145773	FAM81A	0.2060
145748	LYSMD4	0.2054
23266	ADGRL2	0.2046
54997	TESC	0.2046
2324	FLT4	0.2044
9627	SNCAIP	0.2041
3827	KNG1	0.2037
2049	EPHB3	0.2033
9984	THOC1	0.2029
92840	REEP6	0.2027
9129	PRPF3	0.2025
56929	FEM1C	0.2024
117177	RAB3IP	0.2022
92	ACVR2A	0.2021
27255	CNTN6	0.2020
3295	HSD17B4	0.2018
10972	TMED10	0.2018
706	TSPO	0.2017
56675	NRIP3	0.2016
152330	CNTN4	0.2008
27087	B3GAT1	0.2004
929	CD14	0.2001
1653	DDX1	0.1997
170591	S100Z	0.1991
25945	NECTIN3	0.1991
116985	ARAP1	0.1987
23612	PHLDA3	0.1985
51196	PLCE1	0.1985

9905	SGSM2	0.1985
9840	TESPA1	0.1983
221184	CPNE2	0.1982
6194	RPS6	0.1982
221545	C6orf136	0.1981
9590	AKAP12	0.1978
2822	GPLD1	0.1978
766	CA7	0.1978
26152	ZNF337	0.1975
6487	ST3GAL3	0.1973
3783	KCNN4	0.1972
492311	IGIP	0.1971
84864	RIOX2	0.1971
5817	PVR	0.1969
3177	SLC29A2	0.1966
9771	RAPGEF5	0.1965
6616	SNAP25	0.1965
1010	CDH12	0.1963
339983	NAT8L	0.1960
246330	PELI3	0.1959
8655	DYNLL1	0.1958
29803	REPIN1	0.1956
85021	REPS1	0.1955
91252	SLC39A13	0.1954
10614	HEXIM1	0.1953
5549	PRELP	0.1949
494513	PJVK	0.1949
2078	ERG	0.1949
3488	IGFBP5	0.1944
4337	MOCS1	0.1940
10402	ST3GAL6	0.1940
3709	ITPR2	0.1938
1525	CXADR	0.1937
8906	AP1G2	0.1929
84851	TRIM52	0.1928
5685	PSMA4	0.1927
9325	TRIP4	0.1926
56953	NT5M	0.1925
80258	EFHC2	0.1924
154661	RUNDC3B	0.1924
8832	CD84	0.1924
6576	SLC25A1	0.1920
51093	RRNAD1	0.1920
126731	CCSAP	0.1920
254263	CNIH2	0.1920
83656	FAM167A-AS1	0.1919
9515	STXBP5L	0.1915
120114	FAT3	0.1913
55809	TRERF1	0.1913
100294145	LOC100294145	0.1912
8635	RNASET2	0.1911
79745	CLIP4	0.1905
51479	ANKFY1	0.1900
22936	ELL2	0.1895
7260	EIPR1	0.1893

8754	ADAM9	0.1890
5190	PEX6	0.1885
8653	DDX3Y	0.1884
7089	TLE2	0.1884
131034	CPNE4	0.1882
5814	PURB	0.1879
23329	TBC1D30	0.1878
5923	RASGRF1	0.1877
1512	CTSH	0.1876
339487	ZBTB8OS	0.1869
27085	MTBP	0.1865
154790	CLEC2L	0.1864
114907	FBXO32	0.1864
9911	TMCC2	0.1863
23109	DDN	0.1863
2230	FDX1	0.1861
90	ACVR1	0.1857
79033	ERI3	0.1850
56648	EIF5A2	0.1850
57182	ANKRD50	0.1849
58493	INIP	0.1841
79585	CORO7	0.1839
646405	TPTE2P1	0.1836
23613	ZMYND8	0.1834
23014	FBXO21	0.1833
10592	SMC2	0.1832
10204	NUTF2	0.1830
84649	DGAT2	0.1829
8455	ATRN	0.1828
7538	ZFP36	0.1827
7111	TMOD1	0.1825
6508	SLC4A3	0.1823
57446	NDRG3	0.1819
7707	ZNF148	0.1816
357	SHROOM2	0.1814
22	ABCB7	0.1812
123036	TC2N	0.1808
10171	RCL1	0.1805
132789	GNPDA2	0.1797
79754	ASB13	0.1796
5064	PALM	0.1796
79937	CNTNAP3	0.1794
10261	IGSF6	0.1791
23270	TSPYL4	0.1790
831	CAST	0.1788
23261	CAMTA1	0.1785
4087	SMAD2	0.1783
4507	MTAP	0.1779
23284	ADGRL3	0.1779
84159	ARID5B	0.1779
4674	NAP1L2	0.1776
55118	CRTAC1	0.1773
90665	TBL1Y	0.1772
9901	SRGAP3	0.1769
23092	ARHGAP26	0.1768

83707	TRPT1	0.1765
84803	GPAT3	0.1759
27241	BBS9	0.1758
5816	PVALB	0.1751
285598	ARL10	0.1751
1129	CHRM2	0.1744
23052	ENDOD1	0.1743
387763	C11orf96	0.1736
10018	BCL2L11	0.1732
23318	ZCCHC11	0.1731
84898	PLXDC2	0.1731
128178	EDARADD	0.1730
24139	EML2	0.1729
6833	ABCC8	0.1729
9454	HOMER3	0.1728
84128	WDR75	0.1728
11064	CNTRL	0.1728
126917	IFFO2	0.1727
6096	RORB	0.1725
225	ABCD2	0.1724
57468	SLC12A5	0.1722
30819	KCNIP2	0.1720
10460	TACC3	0.1716
63874	ABHD4	0.1716
158747	MOSPD2	0.1702
794	CALB2	0.1699
858	CAV2	0.1693
93474	ZNF670	0.1693
64218	SEMA4A	0.1691
6196	RPS6KA2	0.1683
1047	CLGN	0.1678
518	ATP5MC3	0.1670
5519	PPP2R1B	0.1666
51312	SLC25A37	0.1666
132946	ARL9	0.1665
10231	RCAN2	0.1656
220441	RNF152	0.1655
285613	RELL2	0.1654
6565	SLC15A2	0.1653
6323	SCN1A	0.1648
89782	LMLN	0.1646
79659	DYNC2H1	0.1646
149992	ANKRD30BP2	0.1643
5699	PSMB10	0.1642
129684	CNTNAP5	0.1641
3787	KCNS1	0.1640
55384	MEG3	0.1638
6230	RPS25	0.1638
1774	DNASE1L1	0.1634
55166	CENPQ	0.1634
4547	MTTP	0.1633
79073	TMEM109	0.1630
7704	ZBTB16	0.1629
7991	TUSC3	0.1629
64745	METTL17	0.1626

285203	EOGT	0.1624
2357	FPR1	0.1621
10564	ARFGEF2	0.1620
55175	KLHL11	0.1614
2849	GPR26	0.1612
3945	LDHB	0.1611
92293	TMEM132C	0.1611
56925	LXN	0.1606
7436	VLDLR	0.1605
3688	ITGB1	0.1604
133121	ENPP6	0.1602
3135	HLA-G	0.1599
117	ADCYAP1R1	0.1593
1027	CDKN1B	0.1591
10318	TNIP1	0.1591
713	C1QB	0.1586
80036	TRPM3	0.1585
5453	POU3F1	0.1583
89953	KLC4	0.1578
94	ACVRL1	0.1578
55802	DCP1A	0.1576
10194	TSHZ1	0.1574
84263	HSDL2	0.1572
51108	METTL9	0.1570
283554	GPR137C	0.1568
22955	SCMH1	0.1568
1843	DUSP1	0.1566
5494	PPM1A	0.1565
4212	MEIS2	0.1564
10652	YKT6	0.1560
29945	ANAPC4	0.1560
65992	DDRGK1	0.1559
3908	LAMA2	0.1557
6049	RNF6	0.1557
6653	SORL1	0.1555
977	CD151	0.1555
5634	PRPS2	0.1554
83604	TMEM47	0.1553
7103	TSPAN8	0.1550
57282	SLC4A10	0.1548
337876	CHSY3	0.1547
9727	RAB11FIP3	0.1547
255101	CFAP65	0.1545
23642	SNHG1	0.1544
84690	SPATA22	0.1539
10083	USH1C	0.1539
3137	HLA-J	0.1539
22824	HSPA4L	0.1530
55825	PECR	0.1526
2109	ETFB	0.1523
8992	ATP6V0E1	0.1522
23302	WSCD1	0.1522
246175	CNOT6L	0.1520
670	BPHL	0.1514
23199	GSE1	0.1511

84957	RELT	0.1511
84260	TCHP	0.1508
23386	NUDCD3	0.1508
7078	TIMP3	0.1508
7941	PLA2G7	0.1503
23158	TBC1D9	0.1501
51177	PLEKHO1	0.1501
10875	FGL2	0.1498
5533	PPP3CC	0.1493
3097	HIVEP2	0.1493
23105	FSTL4	0.1492
51163	DBR1	0.1479
6678	SPARC	0.1479
9655	SOCS5	0.1479
84709	MGARP	0.1475
123720	WHAMM	0.1466
55249	YY1AP1	0.1464
4217	MAP3K5	0.1458
7018	TF	0.1456
79109	MAPKAP1	0.1455
29978	UBQLN2	0.1452
79178	THTPA	0.1451
10395	DLC1	0.1448
84324	SARNP	0.1447
2796	GNRH1	0.1446
149076	ZNF362	0.1445
283596	SNHG10	0.1444
56256	SERTAD4	0.1442
84897	TBRG1	0.1441
25937	WWTR1	0.1440
5794	PTPRH	0.1439
272	AMPD3	0.1439
8837	CFLAR	0.1434
386617	KCTD8	0.1430
54102	CLIC6	0.1430
54487	DGCR8	0.1429
83544	DNAL1	0.1427
85440	DOCK7	0.1427
200407	CREG2	0.1425
23451	SF3B1	0.1425
79767	ELMO3	0.1424
9770	RASSF2	0.1421
27324	TOX3	0.1420
5604	MAP2K1	0.1417
81848	SPRY4	0.1414
10602	CDC42EP3	0.1413
4215	MAP3K3	0.1412
53938	PPIL3	0.1408
252995	FNDC5	0.1407
5775	PTPN4	0.1406
60685	ZFAND3	0.1405
6801	STRN	0.1405
5074	PAWR	0.1405
767	CA8	0.1403
25769	SLC24A2	0.1403

2171	FABP5	0.1399
3597	IL13RA1	0.1398
22977	AKR7A3	0.1397
3350	HTR1A	0.1395
29766	TMOD3	0.1392
84940	CORO6	0.1390
54793	KCTD9	0.1389
151009	LINC01106	0.1386
7025	NR2F1	0.1384
54205	CYCS	0.1380
84542	KIAA1841	0.1380
27254	CSDC2	0.1379
1893	ECM1	0.1377
5592	PRKG1	0.1374
5201	PFDN1	0.1372
7559	ZNF12	0.1371
1240	CMKLR1	0.1370
64968	MRPS6	0.1369
3382	ICA1	0.1365
23241	PACS2	0.1363
25825	BACE2	0.1363
5336	PLCG2	0.1360
5111	PCNA	0.1357
5100	PCDH8	0.1355
441212	RP9P	0.1354
25956	SEC31B	0.1351
10562	OLFM4	0.1350
3360	HTR4	0.1343
3164	NR4A1	0.1339
57530	CGN	0.1338
57165	GJC2	0.1337
27199	OXGR1	0.1335
10120	ACTR1B	0.1333
25789	TMEM59L	0.1332
54585	LZTFL1	0.1330
165257	C1QL2	0.1330
94234	FOXQ1	0.1328
10522	DEAF1	0.1326
3275	PRMT2	0.1325
23321	TRIM2	0.1325
23767	FLRT3	0.1323
6253	RTN2	0.1322
146760	RTN4RL1	0.1322
253827	MSRB3	0.1321
169981	SPIN3	0.1317
7994	KAT6A	0.1317
7866	IFRD2	0.1310
91647	ATPAF2	0.1309
54961	SSH3	0.1304
253152	EPHX4	0.1303
203068	TUBB	0.1303
80185	TTI2	0.1302
25841	ABTB2	0.1301
5860	QDPR	0.1300
55853	IDI2-AS1	0.1299

23492	CBX7	0.1298
79147	FKRP	0.1298
1149	CIDEA	0.1297
254531	LPCAT4	0.1296
79626	TNFAIP8L2	0.1295
284723	SLC25A34	0.1294
57541	ZNF398	0.1292
2805	GOT1	0.1285
3418	IDH2	0.1284
9493	KIF23	0.1281
7551	ZNF3	0.1280
23191	CYFIP1	0.1280
5098	PCDHGC3	0.1277
83959	SLC4A11	0.1276
54329	GPR85	0.1274
10627	MYL12A	0.1273
93550	ZFAND4	0.1271
6863	TAC1	0.1265
1386	ATF2	0.1264
5281	PIGF	0.1264
254427	PROSER2	0.1263
288	ANK3	0.1261
402117	VWC2L	0.1261
54913	RPP25	0.1260
48	ACO1	0.1256
54878	DPP8	0.1255
114041	B3GALT5-AS1	0.1254
7705	ZNF146	0.1253
353116	RILPL1	0.1252
9228	DLGAP2	0.1248
84946	LTV1	0.1245
374946	DRAXIN	0.1244
10569	SLU7	0.1243
80279	CDK5RAP3	0.1242
254170	FBXO33	0.1242
122416	ANKRD9	0.1241
81031	SLC2A10	0.1241
9790	BMS1	0.1239
64147	KIF9	0.1237
483	ATP1B3	0.1237
206358	SLC36A1	0.1232
55361	PI4K2A	0.1229
23274	CLEC16A	0.1227
1514	CTSL	0.1226
10238	DCAF7	0.1224
9480	ONECUT2	0.1221
1889	ECE1	0.1219
84978	FRMD5	0.1216
23359	FAM189A1	0.1212
4145	MATK	0.1210
10902	BRD8	0.1209
1606	DGKA	0.1209
89801	PPP1R3F	0.1208
4784	NFIX	0.1207
2952	GSTT1	0.1204

2254	FGF9	0.1204
5955	RCN2	0.1200
146177	VWA3A	0.1197
6850	SYK	0.1187
80778	ZNF34	0.1185
51308	REEP2	0.1185
10413	YAP1	0.1181
6599	SMARCC1	0.1179
256949	KANK3	0.1177
25957	PNISR	0.1175
1130	LYST	0.1175
6892	TAPBP	0.1173
8407	TAGLN2	0.1171
7223	TRPC4	0.1170
687	KLF9	0.1170
7112	TMPO	0.1170
898	CCNE1	0.1170
6426	SRSF1	0.1169
2766	GMPR	0.1167
84626	KRBA1	0.1162
51195	RAPGEFL1	0.1158
4357	MPST	0.1158
81	ACTN4	0.1154
9759	HDAC4	0.1153
4591	TRIM37	0.1153
123041	SLC24A4	0.1147
2294	FOXF1	0.1147
476	ATP1A1	0.1145
341359	SYT10	0.1141
55795	PCID2	0.1141
53919	SLCO1C1	0.1140
150209	AIFM3	0.1137
6641	SNTB1	0.1134
27445	PCLO	0.1134
8202	NCOA3	0.1133
4176	MCM7	0.1131
64101	LRRC4	0.1129
5891	MOK	0.1129
1850	DUSP8	0.1128
5579	PRKCB	0.1124
342926	ZNF677	0.1123
222553	SLC35F1	0.1121
6014	RIT2	0.1120
64123	ADGRL4	0.1118
54625	PARP14	0.1117
54674	LRRN3	0.1115
9024	BRSK2	0.1111
10092	ARPC5	0.1111
256586	LYSMD2	0.1109
7554	ZNF8	0.1107
5644	PRSS1	0.1107
150622	LINC01105	0.1100
11167	FSTL1	0.1098
54843	SYTL2	0.1094
5230	PGK1	0.1094

57415	C3orf14	0.1093
9452	ITM2A	0.1093
22976	PAXIP1	0.1091
83873	GPR61	0.1085
6548	SLC9A1	0.1081
11006	LILRB4	0.1081
145173	B3GLCT	0.1080
23189	KANK1	0.1075
23122	CLASP2	0.1072
29108	PYCARD	0.1072
1974	EIF4A2	0.1070
1316	KLF6	0.1069
55359	STYK1	0.1068
91703	ACY3	0.1066
994	CDC25B	0.1066
613212	CTXN3	0.1061
54982	CLN6	0.1061
220359	TIGD3	0.1057
11341	SCRG1	0.1055
24147	FJX1	0.1052
55909	BIN3	0.1050
10361	NPM2	0.1048
23263	MCF2L	0.1048
57185	NIPAL3	0.1047
9127	P2RX6	0.1045
7837	PXDN	0.1043
7087	ICAM5	0.1042
5357	PLS1	0.1039
1181	CLCN2	0.1032
3777	KCNK3	0.1023
221294	NT5DC1	0.1022
28978	TMEM14A	0.1022
6618	SNAPC2	0.1021
3937	LCP2	0.1019
163115	ZNF781	0.1019
28227	PPP2R3B	0.1017
54965	PIGX	0.1017
1462	VCAN	0.1016
125058	TBC1D16	0.1015
134266	GRPEL2	0.1010
2124	EVI2B	0.1008
4121	MAN1A1	0.1005
22928	SEPHS2	0.1003
117178	SSX2IP	0.1002
24141	LAMP5	0.1000
9592	IER2	0.1000
23596	OPN3	0.0998
23600	AMACR	0.0996
55698	RADIL	0.0993
5800	PTPRO	0.0988
6122	RPL3	0.0988
55212	BBS7	0.0986
93	ACVR2B	0.0985
11281	POU6F2	0.0984
57577	CCDC191	0.0982

8030	CCDC6	0.0982
167681	PRSS35	0.0982
2917	GRM7	0.0981
6326	SCN2A	0.0978
10445	MCRS1	0.0976
4336	MOBP	0.0972
5788	PTPRC	0.0970
6447	SCG5	0.0970
80310	PDGFD	0.0969
333926	PPM1J	0.0967
84620	ST6GAL2	0.0963
51009	DERL2	0.0962
285600	KIAA0825	0.0961
2913	GRM3	0.0961
641649	TMEM91	0.0958
2350	FOLR2	0.0956
2784	GNB3	0.0953
84276	NICN1	0.0950
398	ARHGDIG	0.0950
55711	FAR2	0.0950
55790	CSGALNACT1	0.0947
196968	DNM1P46	0.0946
445	ASS1	0.0946
4801	NFYB	0.0945
79990	PLEKHH3	0.0942
84173	ELMOD3	0.0942
160760	PPTC7	0.0941
85352	SHISAL1	0.0940
4783	NFIL3	0.0935
10308	ZNF267	0.0934
51172	NAGPA	0.0933
23224	SYNE2	0.0929
1107	CHD3	0.0929
55342	STRBP	0.0929
57689	LRRC4C	0.0928
8987	STBD1	0.0927
10439	OLFM1	0.0921
285761	DCBLD1	0.0919
112464	CAVIN3	0.0917
65009	NDRG4	0.0917
151742	PPM1L	0.0910
285126	DNAJC5G	0.0908
714	C1QC	0.0907
338811	FAM19A2	0.0906
64714	PDIA2	0.0904
113263	GLCCI1	0.0900
3136	HLA-H	0.0893
57482	KIAA1211	0.0890
55344	PLCXD1	0.0883
84125	LRRIQ1	0.0883
585	BBS4	0.0879
7045	TGFBI	0.0877
284339	TMEM145	0.0877
57103	TIGAR	0.0876
8581	LY6D	0.0871

51440	HPCAL4	0.0871
1123	CHN1	0.0870
5330	PLCB2	0.0869
23213	SULF1	0.0863
6330	SCN4B	0.0863
79960	JADE1	0.0862
84258	SYT3	0.0862
2170	FABP3	0.0860
84262	PSMG3	0.0858
140688	NOL4L	0.0854
160851	DGKH	0.0853
57709	SLC7A14	0.0849
9315	NREP	0.0847
54148	MRPL39	0.0847
27128	CYTH4	0.0846
397	ARHGDIB	0.0839
9495	AKAP5	0.0837
79966	SCD5	0.0835
54407	SLC38A2	0.0834
6696	SPP1	0.0833
5727	PTCH1	0.0832
81846	SBF2	0.0829
57554	LRRC7	0.0828
9037	SEMA5A	0.0823
10098	TSPAN5	0.0823
25900	IFFO1	0.0823
26056	RAB11FIP5	0.0822
11037	STON1	0.0822
126668	TDRD10	0.0815
1289	COL5A1	0.0815
55367	PIDD1	0.0814
7975	MAFK	0.0813
23137	SMC5	0.0813
4987	OPRL1	0.0813
79844	ZDHHC11	0.0809
7494	XBP1	0.0807
5139	PDE3A	0.0807
563	AZGP1	0.0807
256130	TMEM196	0.0806
261729	STEAP2	0.0806
195814	SDR16C5	0.0806
57679	ALS2	0.0805
10891	PPARGC1A	0.0804
84109	QRFPR	0.0803
8908	GYG2	0.0802
7678	ZNF124	0.0802
1812	DRD1	0.0801
4601	MXI1	0.0798
23221	RHOBTB2	0.0798
51061	TXNDC11	0.0797
83992	CTTNBP2	0.0793
84867	PTPN5	0.0790
51085	MLXIPL	0.0789
6907	TBL1X	0.0787
10631	POSTN	0.0782

26007	TKFC	0.0781
11043	MID2	0.0771
55824	PAG1	0.0770
6001	RGS10	0.0767
9353	SLIT2	0.0762
29953	TRHDE	0.0762
29118	DDX25	0.0760
6334	SCN8A	0.0759
8999	CDKL2	0.0758
4915	NTRK2	0.0758
51542	VPS54	0.0757
283234	CCDC88B	0.0755
9772	TMEM94	0.0754
162681	C18orf54	0.0752
3485	IGFBP2	0.0744
91300	R3HDM4	0.0744
9252	RPS6KA5	0.0744
5467	PPARD	0.0739
64949	MRPS26	0.0737
8767	RIPK2	0.0733
89894	TMEM116	0.0732
5116	PCNT	0.0730
1727	CYB5R3	0.0729
10254	STAM2	0.0728
55784	MCTP2	0.0728
2242	FES	0.0726
8877	SPHK1	0.0723
54509	RHOF	0.0720
3035	HARS	0.0720
54704	PDP1	0.0716
57134	MAN1C1	0.0713
131583	FAM43A	0.0713
7553	ZNF7	0.0712
1299	COL9A3	0.0711
10036	CHAF1A	0.0708
114800	CCDC85A	0.0707
27143	PALD1	0.0704
29919	RMC1	0.0703
90139	TSPAN18	0.0703
1152	CKB	0.0699
492	ATP2B3	0.0699
400720	ZNF772	0.0697
10005	ACOT8	0.0696
90187	EMILIN3	0.0696
126755	LRRC38	0.0695
3107	HLA-C	0.0695
55796	MBNL3	0.0693
221981	THSD7A	0.0689
285527	FRYL	0.0688
222962	SLC29A4	0.0687
51295	ECSIT	0.0687
66004	LYNX1	0.0687
404037	HAPLN4	0.0686
8013	NR4A3	0.0685
3843	IPO5	0.0685

2043	EPHA4	0.0685
1297	COL9A1	0.0684
6775	STAT4	0.0683
9760	TOX	0.0681
55139	ANKZF1	0.0676
8507	ENC1	0.0676
199	AIF1	0.0675
80852	GRIP2	0.0675
23072	HECW1	0.0670
84440	RAB11FIP4	0.0668
402381	SOHLH1	0.0667
23236	PLCB1	0.0667
10811	NOXA1	0.0666
7905	REEP5	0.0666
1762	DMWD	0.0664
10025	MED16	0.0658
55081	IFT57	0.0657
49854	ZBTB21	0.0657
6993	DYNLT1	0.0657
9447	AIM2	0.0655
29760	BLNK	0.0654
5152	PDE9A	0.0652
400892	BCRP2	0.0651
283638	CEP170B	0.0651
6320	CLEC11A	0.0651
5026	P2RX5	0.0650
80896	NPL	0.0649
60491	NIF3L1	0.0649
3119	HLA-DQB1	0.0649
6875	TAF4B	0.0649
6890	TAP1	0.0643
87	ACTN1	0.0642
9584	RBM39	0.0642
23479	ISCU	0.0641
4157	MC1R	0.0637
5918	RARRES1	0.0636
6455	SH3GL1	0.0636
754	PTTG1IP	0.0635
10388	SYCP2	0.0633
4833	NME4	0.0632
7450	VWF	0.0631
5447	POR	0.0629
388121	TNFAIP8L3	0.0626
4067	LYN	0.0625
3746	KCNC1	0.0620
653247	PRB2	0.0617
5805	PTS	0.0616
57019	CIAPIN1	0.0615
55196	KIAA1551	0.0615
342184	FMN1	0.0611
154197	PNLDC1	0.0607
59352	LGR6	0.0607
9924	PAN2	0.0606
259173	ALS2CL	0.0605
8817	FGF18	0.0600

5188	GATB	0.0600
59283	CACNG8	0.0597
26010	SPATS2L	0.0594
9709	HERPUD1	0.0593
4017	LOXL2	0.0591
4286	MITF	0.0589
151126	ZNF385B	0.0589
22921	MSRB2	0.0582
55970	GNG12	0.0571
10630	PDPN	0.0571
55968	NSFL1C	0.0568
10743	RAI1	0.0566
11186	RASSF1	0.0566
57125	PLXDC1	0.0562
8822	FGF17	0.0561
23398	PPWD1	0.0560
133522	PPARGC1B	0.0559
22866	CNKSR2	0.0558
6785	ELOVL4	0.0558
8975	USP13	0.0558
56124	PCDHB12	0.0555
343263	MYBPHL	0.0550
2534	FYN	0.0550
161779	PGBD4	0.0548
6095	RORA	0.0539
27075	TSPAN13	0.0537
79139	DERL1	0.0537
79145	CHCHD7	0.0536
71	ACTG1	0.0536
83451	ABHD11	0.0535
689	BTF3	0.0534
1476	CSTB	0.0532
578	BAK1	0.0532
93624	TADA2B	0.0528
57633	LRRN1	0.0527
115825	WDFY2	0.0527
223	ALDH9A1	0.0524
1622	DBI	0.0521
3145	HMBS	0.0521
57396	CLK4	0.0520
9543	IGDCC3	0.0520
347404	LANCL3	0.0520
79072	FASTKD3	0.0517
6894	TARBP1	0.0516
3790	KCNS3	0.0515
51149	MRNIP	0.0514
9658	ZNF516	0.0513
5378	PMS1	0.0512
116150	NUS1	0.0507
23433	RHOQ	0.0505
8087	FXR1	0.0503
4753	NELL2	0.0501
6275	S100A4	0.0500
348980	HCN1	0.0499
54496	PRMT7	0.0498

1813	DRD2	0.0498
22872	SEC31A	0.0494
222484	LNX2	0.0493
10966	RAB40B	0.0491
3265	HRAS	0.0491
134829	CLVS2	0.0490
112703	FAM71E1	0.0490
30815	ST6GALNAC6	0.0487
57619	SHROOM3	0.0486
23331	TTC28	0.0483
129790	C7orf13	0.0481
10100	TSPAN2	0.0481
3914	LAMB3	0.0476
408263	FNDC9	0.0475
84735	CNDP1	0.0475
1294	COL7A1	0.0473
10988	METAP2	0.0473
79677	SMC6	0.0472
51701	NLK	0.0471
157848	NKX6-3	0.0470
54209	TREM2	0.0470
54807	ZNF586	0.0468
5931	RBBP7	0.0462
80765	STARD5	0.0459
285905	INTS4P1	0.0456
144402	CPNE8	0.0456
121642	ALKBH2	0.0455
81544	GDPD5	0.0454
56288	PARD3	0.0454
91147	TMEM67	0.0452
7276	TTR	0.0448
10082	GPC6	0.0447
57645	POGK	0.0447
1266	CNN3	0.0446
728464	METTL24	0.0445
221935	SDK1	0.0443
200132	TCTEX1D1	0.0443
26230	TIAM2	0.0443
4199	ME1	0.0442
54206	ERRFI1	0.0441
6710	SPTB	0.0440
1996	ELAVL4	0.0440
2354	FOSB	0.0439
5214	PFKP	0.0438
58528	RRAGD	0.0433
4190	MDH1	0.0433
91607	SLFN11	0.0432
2182	ACSL4	0.0431
22901	ARSG	0.0429
9379	NRXN2	0.0429
8500	PPFIA1	0.0429
284001	CCDC57	0.0429
79993	ELOVL7	0.0425
93664	CADPS2	0.0424
4214	MAP3K1	0.0423

285016	ALKAL2	0.0423
9683	N4BP1	0.0422
55321	TMEM74B	0.0421
51291	GMIP	0.0420
2648	KAT2A	0.0420
55857	KIZ	0.0420
822	CAPG	0.0419
783	CACNB2	0.0418
84912	SLC35B4	0.0416
79929	MAP6D1	0.0415
55246	CCDC25	0.0415
54948	MRPL16	0.0412
10513	APPBP2	0.0412
7447	VSNL1	0.0410
257	ALX3	0.0406
727800	RNF208	0.0405
22797	TFEC	0.0405
26258	BLOC1S6	0.0404
170961	ANKRD24	0.0401
2900	GRIK4	0.0396
57685	CACHD1	0.0396
29968	PSAT1	0.0394
64759	TNS3	0.0393
8224	SYN3	0.0393
219844	HYLS1	0.0391
63939	FAM217B	0.0388
23081	KDM4C	0.0387
51301	GCNT4	0.0387
6198	RPS6KB1	0.0382
375061	FAM89A	0.0379
7321	UBE2D1	0.0378
56894	AGPAT3	0.0373
2155	F7	0.0370
3788	KCNS2	0.0370
57124	CD248	0.0370
1613	DAPK3	0.0370
55257	MRGBP	0.0368
25927	CNRIP1	0.0368
129804	FBLN7	0.0366
140809	SRXN1	0.0366
1936	EEF1D	0.0365
27242	TNFRSF21	0.0361
79102	RNF26	0.0357
3782	KCNN3	0.0355
1869	E2F1	0.0352
51720	UIMC1	0.0350
441204	LOC441204	0.0349
6613	SUMO2	0.0346
114769	CARD16	0.0343
54518	APBB1IP	0.0342
26118	WSB1	0.0342
127253	TYW3	0.0340
3632	INPP5A	0.0336
160857	CCDC122	0.0334
3479	IGF1	0.0333

339231	ARL16	0.0333
55534	MAML3	0.0332
84765	ZNF577	0.0330
2701	GJA4	0.0327
85442	KNDC1	0.0326
5205	ATP8B1	0.0326
283349	RASSF3	0.0319
27107	ZBTB11	0.0319
84947	SERAC1	0.0318
9662	CEP135	0.0317
5720	PSME1	0.0313
84885	ZDHHC12	0.0312
10224	ZNF443	0.0306
91133	L3MBTL4	0.0303
84928	TMEM209	0.0298
4677	NARS	0.0297
23325	WASHC4	0.0293
613	BCR	0.0292
2787	GNG5	0.0288
26032	SUSD5	0.0287
23593	HEBP2	0.0286
23544	SEZ6L	0.0286
147138	TMC8	0.0285
4240	MFGE8	0.0283
26146	TRAF3IP1	0.0282
79712	GTDC1	0.0280
548596	CKMT1A	0.0280
5538	PPT1	0.0274
5764	PTN	0.0274
6934	TCF7L2	0.0273
79846	CFAP69	0.0272
836	CASP3	0.0271
114971	PTPMT1	0.0270
3371	TNC	0.0269
51466	EVL	0.0269
220004	PPP1R32	0.0264
5583	PRKCH	0.0263
55669	MFN1	0.0259
5310	PKD1	0.0259
9270	ITGB1BP1	0.0257
9577	BABAM2	0.0254
10550	ARL6IP5	0.0245
6691	SPINK2	0.0243
117145	THEM4	0.0242
152302	CIDECP	0.0241
5801	PTPRR	0.0239
5903	RANBP2	0.0237
10240	MRPS31	0.0237
51059	FAM135B	0.0235
221806	VWDE	0.0232
84752	B3GNT9	0.0232
275	AMT	0.0230
64847	SPATA20	0.0229
4867	NPHP1	0.0228
27148	STK36	0.0228

1740	DLG2	0.0225
5586	PKN2	0.0224
25891	PAMR1	0.0224
9274	BCL7C	0.0224
3693	ITGB5	0.0221
51127	TRIM17	0.0221
956	ENTPD3	0.0217
57210	SLC45A4	0.0216
4355	MPP2	0.0214
80325	ABTB1	0.0214
153769	SH3RF2	0.0211
64423	INF2	0.0209
1730	DIAPH2	0.0208
113675	SDSL	0.0208
84513	PLPP5	0.0208
140767	NRSN1	0.0207
9774	BCLAF1	0.0206
56122	PCDHB14	0.0204
10023	FRAT1	0.0202
23676	SMPX	0.0202
134466	ZNF300P1	0.0202
202151	RANBP3L	0.0200
154141	MBOAT1	0.0200
10365	KLF2	0.0197
55671	PPP4R3A	0.0196
27347	STK39	0.0193
56945	MRPS22	0.0190
203111	ERICH5	0.0187
84868	HAVCR2	0.0186
2674	GFRA1	0.0182
27086	FOXP1	0.0177
134429	STARD4	0.0176
10095	ARPC1B	0.0175
338645	LUZP2	0.0175
222537	HS3ST5	0.0174
65057	ACD	0.0171
113026	PLCD3	0.0169
29844	TFPT	0.0169
3635	INPP5D	0.0167
84518	CNFN	0.0164
83938	LRMDA	0.0163
10863	ADAM28	0.0163
80028	FBXL18	0.0163
285498	RNF212	0.0163
4088	SMAD3	0.0161
388284	C16orf86	0.0155
150678	COPS9	0.0155
116842	LEAP2	0.0155
91392	ZNF502	0.0154
146050	ZSCAN29	0.0152
6988	TCTA	0.0150
4638	MYLK	0.0145
285220	EPHA6	0.0144
4124	MAN2A1	0.0143
599	BCL2L2	0.0142

5915	RARB	0.0138
473	RERE	0.0133
9249	DHRS3	0.0130
58189	WFDC1	0.0129
10870	HCST	0.0129
5465	PPARA	0.0128
5050	PAFAH1B3	0.0128
92312	MEX3A	0.0121
1258	CNGB1	0.0119
55966	AJAP1	0.0118
135293	PM20D2	0.0112
9060	PAPSS2	0.0111
770	CA11	0.0111
2551	GABPA	0.0110
6261	RYR1	0.0109
771	CA12	0.0109
50853	VILL	0.0106
158431	ZNF782	0.0106
115677	NOSTRIN	0.0103
654466	FGF7P3	0.0103
25791	NGEF	0.0102
89890	KBTBD6	0.0101
120	ADD3	0.0097
972	CD74	0.0096
55008	HERC6	0.0096
9891	NUAK1	0.0091
10124	ARL4A	0.0089
90324	CCDC97	0.0087
162394	SLFN5	0.0081
10514	MYBBP1A	0.0076
51571	FAM49B	0.0072
55808	ST6GALNAC1	0.0072
6091	ROBO1	0.0072
79742	CXorf36	0.0070
574036	SERTAD4-AS1	0.0070
7798	LUZP1	0.0069
241	ALOX5AP	0.0065
549	AUH	0.0065
6643	SNX2	0.0064
79794	C12orf49	0.0054
53354	PANK1	0.0054
90826	PRMT9	0.0053
84033	OBSCN	0.0051
8440	NCK2	0.0050
5781	PTPN11	0.0048
9710	KIAA0355	0.0045
85004	RERG	0.0045
10716	TBR1	0.0043
55054	ATG16L1	0.0040
3113	HLA-DPA1	0.0038
114	ADCY8	0.0037
56848	SPHK2	0.0034
129530	LYG1	0.0027
1838	DTNB	0.0026
283651	HMGN2P46	0.0025

55353	LAPTM4B	0.0024
3551	IKBKB	0.0024
6658	SOX3	0.0022
495	ATP4A	0.0021
4760	NEUROD1	0.0021
6534	SLC6A7	0.0021
2556	GABRA3	0.0018
2053	EPHX2	0.0018
65124	SOWAHC	0.0017
1325	CORT	0.0016
389421	LIN28B	0.0013
4729	NDUFV2	0.0013
283985	FADS6	0.0012
28970	C11orf54	0.0010
27294	DHDH	0.0006
23327	NEDD4L	0.0004
117583	PARD3B	-0.0003
135112	NCOA7	-0.0005
353500	BMP8A	-0.0008
84909	C9orf3	-0.0010
10600	USP16	-0.0013
7746	ZSCAN9	-0.0014
23514	SPIDR	-0.0017
3910	LAMA4	-0.0019
387640	SKIDA1	-0.0022
10307	APBB3	-0.0024
222389	BEND7	-0.0025
153579	BTNL9	-0.0026
57159	TRIM54	-0.0031
3936	LCP1	-0.0031
9856	KIAA0319	-0.0032
6814	STXBP3	-0.0033
23432	GPR161	-0.0034
54212	SNTG1	-0.0037
309	ANXA6	-0.0037
222008	VSTM2A	-0.0038
54969	HPF1	-0.0039
53822	FXYD7	-0.0041
1901	S1PR1	-0.0043
7464	CORO2A	-0.0043
439921	MXRA7	-0.0044
79661	NEIL1	-0.0045
5121	PCP4	-0.0046
25948	KBTBD2	-0.0052
131566	DCBLD2	-0.0054
2101	ESRRA	-0.0056
79039	DDX54	-0.0057
5793	PTPRG	-0.0058
11096	ADAMTS5	-0.0062
84872	ZC3H10	-0.0064
642475	MROH6	-0.0069
396	ARHGDIA	-0.0070
2016	EMX1	-0.0072
3663	IRF5	-0.0072
22841	RAB11FIP2	-0.0074

4122	MAN2A2	-0.0077
100113407	TMEM170B	-0.0078
6222	RPS18	-0.0078
4501	MT1X	-0.0079
1915	EEF1A1	-0.0080
55323	LARP6	-0.0082
7775	ZNF232	-0.0084
27430	MAT2B	-0.0086
64771	C6orf106	-0.0086
8195	MKKS	-0.0089
117248	GALNT15	-0.0091
23002	DAAM1	-0.0091
80774	LIMD2	-0.0091
28955	DEXI	-0.0092
3575	IL7R	-0.0098
83931	STK40	-0.0100
22898	DENND3	-0.0101
7139	TNNT2	-0.0105
23589	CARHSP1	-0.0105
23228	PLCL2	-0.0106
85461	TANC1	-0.0107
203328	SUSD3	-0.0110
144348	ZNF664	-0.0111
23362	PSD3	-0.0112
253559	CADM2	-0.0115
7347	UCHL3	-0.0115
375449	MAST4	-0.0116
54857	GDPD2	-0.0116
56999	ADAMTS9	-0.0116
10350	ABCA9	-0.0118
23141	ANKLE2	-0.0118
6903	TBCC	-0.0119
286097	MICU3	-0.0120
1763	DNA2	-0.0120
64417	TMEM267	-0.0122
283871	PGP	-0.0124
388962	BOLA3	-0.0126
775	CACNA1C	-0.0126
1128	CHRM1	-0.0133
53340	SPA17	-0.0134
29906	ST8SIA5	-0.0135
84062	DTNBP1	-0.0138
6141	RPL18	-0.0138
53826	FXYD6	-0.0140
55056	GABPB1-IT1	-0.0144
23421	ITGB3BP	-0.0145
5082	PDCL	-0.0145
442319	ZNF727	-0.0146
1620	BRINP1	-0.0149
902	CCNH	-0.0149
8991	SELENBP1	-0.0152
9717	SEC14L5	-0.0153
2878	GPX3	-0.0154
54914	FOCAD	-0.0155
54839	LRRC49	-0.0156

84939	MUM1	-0.0157
163255	ZNF540	-0.0157
197021	LCTL	-0.0159
2566	GABRG2	-0.0162
114880	OSBPL6	-0.0164
54981	NMRK1	-0.0168
84254	CAMKK1	-0.0169
84514	GHDC	-0.0169
53373	TPCN1	-0.0170
90957	DHX57	-0.0171
113444	SMIM12	-0.0172
11278	KLF12	-0.0175
1522	CTSZ	-0.0178
53346	TM6SF1	-0.0178
2946	GSTM2	-0.0180
121512	FGD4	-0.0181
9960	USP3	-0.0182
1070	CETN3	-0.0183
79075	DSCC1	-0.0184
54841	BIVM	-0.0186
10907	TXNL4A	-0.0191
5630	PRPH	-0.0196
26040	SETBP1	-0.0196
6742	SSBP1	-0.0197
6615	SNAI1	-0.0197
8505	PARG	-0.0197
8506	CNTNAP1	-0.0199
257000	TINCR	-0.0199
8395	PIP5K1B	-0.0201
54805	CNNM2	-0.0204
5144	PDE4D	-0.0205
130589	GALM	-0.0205
4332	MNDA	-0.0205
54812	AFTPH	-0.0207
10370	CITED2	-0.0213
22996	TTC39A	-0.0222
56906	THAP10	-0.0224
6734	SRPRA	-0.0227
23417	MLYCD	-0.0227
196394	AMN1	-0.0228
9218	VAPA	-0.0230
53829	P2RY13	-0.0230
6676	SPAG4	-0.0231
784	CACNB3	-0.0231
22865	SLITRK3	-0.0233
5122	PCSK1	-0.0235
25975	EGFL6	-0.0236
1031	CDKN2C	-0.0236
57476	GRAMD1B	-0.0237
153129	SLC38A9	-0.0237
84612	PARD6B	-0.0240
57827	C6orf47	-0.0243
81565	NDEL1	-0.0243
122953	JDP2	-0.0244
646113	LINC00643	-0.0245

2009	EML1	-0.0247
120224	TMEM45B	-0.0247
80760	ITIH5	-0.0249
132	ADK	-0.0255
9697	TRAM2	-0.0255
219333	USP12	-0.0257
93099	DMKN	-0.0257
51278	IER5	-0.0259
115	ADCY9	-0.0261
11164	NUDT5	-0.0263
284306	ZNF547	-0.0264
80206	FHOD3	-0.0266
116966	WDR17	-0.0267
1524	CX3CR1	-0.0267
64901	RANBP17	-0.0267
11273	ATXN2L	-0.0270
6285	S100B	-0.0271
3601	IL15RA	-0.0273
968	CD68	-0.0274
9918	NCAPD2	-0.0275
23046	KIF21B	-0.0278
9531	BAG3	-0.0279
80307	FER1L4	-0.0279
6541	SLC7A1	-0.0280
64319	FBRS	-0.0284
4747	NEFL	-0.0284
3455	IFNAR2	-0.0285
54103	GSAP	-0.0285
4036	LRP2	-0.0287
79962	DNAJC22	-0.0287
83606	GUCD1	-0.0288
1429	CRYZ	-0.0289
255043	TMEM86B	-0.0291
4168	MCF2	-0.0291
10874	NMU	-0.0293
144811	LACC1	-0.0294
374378	GALNT18	-0.0296
377677	CA13	-0.0297
254102	EHBP1L1	-0.0297
91543	RSAD2	-0.0298
201164	PLD6	-0.0298
3590	IL11RA	-0.0303
5141	PDE4A	-0.0303
153020	RASGEF1B	-0.0304
712	C1QA	-0.0305
23259	DDHD2	-0.0306
6272	SORT1	-0.0306
54437	SEMA5B	-0.0313
26507	CNNM1	-0.0316
1075	CTSC	-0.0316
5119	CHMP1A	-0.0317
64374	SIL1	-0.0320
84230	LRRC8C	-0.0320
57863	CADM3	-0.0320
83787	ARMC10	-0.0321

55860	ACTR10	-0.0321
51002	TPRKB	-0.0322
124976	SPNS2	-0.0326
9079	LDB2	-0.0326
25814	ATXN10	-0.0327
9949	AMMECR1	-0.0327
9063	PIAS2	-0.0328
8544	PIR	-0.0329
79720	VPS37B	-0.0330
2790	GNG10	-0.0330
79867	TCTN2	-0.0330
9781	RNF144A	-0.0332
1960	EGR3	-0.0336
54504	CPVL	-0.0340
100272147	CMC4	-0.0345
6018	RLF	-0.0345
80055	PGAP1	-0.0346
26509	MYOF	-0.0347
5745	PTH1R	-0.0348
163782	KANK4	-0.0348
51762	RAB8B	-0.0350
3633	INPP5B	-0.0353
10123	ARL4C	-0.0355
301	ANXA1	-0.0356
10715	CERS1	-0.0357
221424	LRRC73	-0.0363
404672	GTF2H5	-0.0363
10536	P3H3	-0.0364
2710	GK	-0.0364
8555	CDC14B	-0.0365
389073	C2orf80	-0.0367
10561	IFI44	-0.0368
64926	RASAL3	-0.0369
3705	ITPK1	-0.0372
54875	CNTLN	-0.0372
58492	ZNF77	-0.0375
9121	SLC16A5	-0.0378
2052	EPHX1	-0.0380
55297	CCDC91	-0.0383
11188	NISCH	-0.0383
55033	FKBP14	-0.0384
7097	TLR2	-0.0390
55779	CFAP44	-0.0390
286826	LIN9	-0.0391
79624	ARMT1	-0.0392
79791	FBXO31	-0.0392
284119	CAVIN1	-0.0395
7187	TRAF3	-0.0395
63920	ZBED8	-0.0397
9120	SLC16A6	-0.0397
1662	DDX10	-0.0398
4345	CD200	-0.0400
23341	DNAJC16	-0.0401
1845	DUSP3	-0.0403
89927	C16orf45	-0.0404

55100	WDR70	-0.0405
1050	CEBPA	-0.0405
4258	MGST2	-0.0407
10166	SLC25A15	-0.0409
253582	TMEM244	-0.0411
255275	MYADML2	-0.0412
2348	FOLR1	-0.0414
818	CAMK2G	-0.0416
1427	CRYGS	-0.0417
10758	TRAF3IP2	-0.0417
4233	MET	-0.0417
283420	CLEC9A	-0.0418
168090	C6orf118	-0.0419
2775	GNAO1	-0.0419
5066	PAM	-0.0420
84059	ADGRV1	-0.0420
92715	DPH7	-0.0422
219621	CABCOCO1	-0.0422
79957	PAQR6	-0.0422
2746	GLUD1	-0.0423
131474	CHCHD4	-0.0423
5099	PCDH7	-0.0430
57018	CCNL1	-0.0431
402665	IGLON5	-0.0433
23370	ARHGEF18	-0.0434
80177	MYCT1	-0.0437
124857	WFIKKN2	-0.0440
5184	PEPD	-0.0442
7739	ZNF185	-0.0443
57333	RCN3	-0.0445
117283	IP6K3	-0.0451
653483	AFDN-DT	-0.0451
2214	FCGR3A	-0.0452
8534	CHST1	-0.0452
201625	DNAH12	-0.0457
5786	PTPRA	-0.0462
8833	GMPS	-0.0463
6715	SRD5A1	-0.0464
79570	NKAIN1	-0.0464
84525	HOPX	-0.0465
26122	EPC2	-0.0466
5509	PPP1R3D	-0.0467
81704	DOCK8	-0.0467
1198	CLK3	-0.0468
81605	URM1	-0.0468
8853	ASAP2	-0.0469
8439	NSMAF	-0.0475
51661	FKBP7	-0.0476
51303	FKBP11	-0.0479
117166	WFIKKN1	-0.0482
56935	SMCO4	-0.0482
55374	TMCO6	-0.0482
51074	APIP	-0.0483
27067	STAU2	-0.0484
51330	TNFRSF12A	-0.0485

26073	POLDIP2	-0.0486
2895	GRID2	-0.0487
2872	MKNK2	-0.0487
81928	CABLES2	-0.0488
4692	NDN	-0.0494
116154	PHACTR3	-0.0495
50854	C6orf48	-0.0503
252983	STXBP4	-0.0503
10161	LPAR6	-0.0503
955	ENTPD6	-0.0505
124401	ANKS3	-0.0506
4498	MT1JP	-0.0506
10290	SPEG	-0.0507
55329	MNS1	-0.0507
222236	NAPEPLD	-0.0508
124923	SGK494	-0.0508
51043	ZBTB7B	-0.0509
963	CD53	-0.0510
10295	BCKDK	-0.0512
442523	DPY19L2P4	-0.0512
7547	ZIC3	-0.0513
51530	ZC3HC1	-0.0515
8553	BHLHE40	-0.0517
3822	KLRC2	-0.0517
9630	GNA14	-0.0522
1135	CHRNA2	-0.0523
114569	MAL2	-0.0529
8685	MARCO	-0.0531
201456	FBXO15	-0.0536
5493	PPL	-0.0537
55902	ACSS2	-0.0539
3108	HLA-DMA	-0.0540
114795	TMEM132B	-0.0540
9020	MAP3K14	-0.0541
390010	NKX1-2	-0.0543
3653	IPW	-0.0544
11072	DUSP14	-0.0545
3032	HADHB	-0.0545
3084	NRG1	-0.0547
57568	SIPA1L2	-0.0549
3196	TLX2	-0.0551
26996	GPR160	-0.0552
55667	DENND4C	-0.0556
4058	LTK	-0.0557
114818	KLHL29	-0.0557
57521	RPTOR	-0.0559
5445	PON2	-0.0561
1412	CRYBA2	-0.0561
222223	KIAA1324L	-0.0562
161436	EML5	-0.0564
115584	SLC5A11	-0.0569
22948	CCT5	-0.0570
55876	GSDMB	-0.0571
158584	FAAH2	-0.0571
6916	TBXAS1	-0.0572

1466	CSRP2	-0.0574
100507436	MICA	-0.0574
10485	C1orf61	-0.0578
148103	ZNF599	-0.0580
254295	PHYHD1	-0.0580
26232	FBXO2	-0.0581
23283	CSTF2T	-0.0582
10655	DMRT2	-0.0582
161835	FSIP1	-0.0584
26278	SACS	-0.0584
83690	CRISPLD1	-0.0585
3290	HSD11B1	-0.0587
8871	SYNJ2	-0.0589
7181	NR2C1	-0.0589
1073	CFL2	-0.0592
2322	FLT3	-0.0592
5871	MAP4K2	-0.0592
580	BARD1	-0.0594
112942	CFAP36	-0.0598
132864	CPEB2	-0.0601
55756	INTS9	-0.0601
4208	MEF2C	-0.0603
10673	TNFSF13B	-0.0603
644	BLVRA	-0.0603
29887	SNX10	-0.0605
253314	EIF4E1B	-0.0607
116115	ZNF526	-0.0607
10170	DHRS9	-0.0609
51735	RAPGEF6	-0.0610
80178	TEDC2	-0.0610
3730	ANOS1	-0.0611
80305	TRABD	-0.0614
6546	SLC8A1	-0.0615
9980	DOPEY2	-0.0619
3770	KCNJ14	-0.0620
4643	MYO1E	-0.0621
149563	SRARP	-0.0622
55815	TSNAXIP1	-0.0623
79064	TMEM223	-0.0624
26355	FAM162A	-0.0624
11151	CORO1A	-0.0627
81631	MAP1LC3B	-0.0631
9330	GTF3C3	-0.0633
4929	NR4A2	-0.0635
84524	ZC3H8	-0.0639
9814	SFI1	-0.0640
338328	GPIHBP1	-0.0641
55182	RNF220	-0.0642
57453	DSCAML1	-0.0643
220992	ZNF485	-0.0644
55515	ASIC4	-0.0644
9023	CH25H	-0.0646
129401	NUP35	-0.0647
1805	DPT	-0.0647
284273	ZADH2	-0.0648

3384	ICAM2	-0.0651
2026	ENO2	-0.0653
1946	EFNA5	-0.0653
1910	EDNRB	-0.0653
65989	DLK2	-0.0654
107	ADCY1	-0.0654
147381	CBLN2	-0.0654
55093	WDYHV1	-0.0654
64135	IFIH1	-0.0656
80762	NDFIP1	-0.0656
7940	LST1	-0.0658
202181	LOC202181	-0.0659
6159	RPL29	-0.0661
8470	SORBS2	-0.0662
9529	BAG5	-0.0662
2996	GYPE	-0.0663
84083	ZRANB3	-0.0663
91584	PLXNA4	-0.0664
5784	PTPN14	-0.0664
1301	COL11A1	-0.0669
84688	C9orf24	-0.0673
65095	KRI1	-0.0674
8905	AP1S2	-0.0675
1490	CTGF	-0.0678
9684	LRRC14	-0.0680
54665	RSBN1	-0.0681
389705	LOC389705	-0.0683
119587	CPXM2	-0.0684
51444	RNF138	-0.0684
29901	SAC3D1	-0.0685
94122	SYTL5	-0.0685
10745	PHTF1	-0.0686
4174	MCM5	-0.0689
57168	ASPHD2	-0.0690
56655	POLE4	-0.0690
4843	NOS2	-0.0690
5595	MAPK3	-0.0691
9355	LHX2	-0.0691
5950	RBP4	-0.0693
10026	PIGK	-0.0693
740	MRPL49	-0.0693
8448	DOC2A	-0.0695
9475	ROCK2	-0.0697
8912	CACNA1H	-0.0697
4005	LMO2	-0.0698
256355	RPS2P32	-0.0699
202559	KHDRBS2	-0.0700
60625	DHX35	-0.0703
219790	RTKN2	-0.0705
26586	CKAP2	-0.0706
23344	ESYT1	-0.0708
1908	EDN3	-0.0710
308	ANXA5	-0.0714
341346	SMCO2	-0.0714
23516	SLC39A14	-0.0716

220001	VWCE	-0.0716
4842	NOS1	-0.0717
10406	WFDC2	-0.0723
10371	SEMA3A	-0.0725
84250	SLF1	-0.0725
10518	CIB2	-0.0726
6448	SGSH	-0.0727
51616	TAF9B	-0.0730
9331	B4GALT6	-0.0737
2744	GLS	-0.0738
5662	PSD	-0.0738
388969	C2orf68	-0.0740
136647	MPLKIP	-0.0740
5155	PDGFB	-0.0741
164633	CABP7	-0.0743
90861	JPT2	-0.0745
55116	TMEM39B	-0.0748
6494	SIPA1	-0.0748
114571	SLC22A9	-0.0748
84632	AFAP1L2	-0.0750
6604	SMARCD3	-0.0751
1775	DNASE1L2	-0.0751
91523	PCED1B	-0.0752
80336	PABPC1L	-0.0752
138639	PTPDC1	-0.0756
92340	PRR29	-0.0759
154091	SLC2A12	-0.0759
10425	ARIH2	-0.0759
326624	RAB37	-0.0760
800	CALD1	-0.0761
11320	MGAT4A	-0.0764
7976	FZD3	-0.0765
151651	EFHB	-0.0768
84539	MCHR2	-0.0772
84945	ABHD13	-0.0772
7718	ZNF165	-0.0773
124936	CYB5D2	-0.0773
7274	TTPA	-0.0774
50940	PDE11A	-0.0774
57758	SCUBE2	-0.0777
57553	MICAL3	-0.0780
54799	MBTD1	-0.0783
6732	SRPK1	-0.0785
64848	YTHDC2	-0.0785
94233	OPN4	-0.0787
163183	SYNE4	-0.0788
150483	TEKT4	-0.0790
145447	ABHD12B	-0.0790
8839	WISP2	-0.0790
134218	DNAJC21	-0.0791
128077	LIX1L	-0.0796
88455	ANKRD13A	-0.0797
7088	TLE1	-0.0797
120425	JAML	-0.0798
8504	PEX3	-0.0800

4050	LTB	-0.0803
1059	CENPB	-0.0805
2289	FKBP5	-0.0806
7164	TPD52L1	-0.0806
126308	MOB3A	-0.0807
8942	KYNU	-0.0807
7098	TLR3	-0.0808
29082	CHMP4A	-0.0809
80342	TRAF3IP3	-0.0810
3122	HLA-DRA	-0.0810
51176	LEF1	-0.0810
57536	KIAA1328	-0.0812
116461	TSEN15	-0.0812
84804	MFSD9	-0.0812
66008	TRAK2	-0.0813
9348	NDST3	-0.0818
5734	PTGER4	-0.0818
54988	ACSM5	-0.0819
84435	ADGRA1	-0.0819
7079	TIMP4	-0.0819
9656	MDC1	-0.0821
55112	WDR60	-0.0821
1635	DCTD	-0.0823
283742	FAM98B	-0.0826
112495	GTF3C6	-0.0827
55244	SLC47A1	-0.0833
7923	HSD17B8	-0.0833
23768	FLRT2	-0.0834
9802	DAZAP2	-0.0835
3067	HDC	-0.0836
28959	TMEM176B	-0.0840
2286	FKBP2	-0.0842
28960	DCPS	-0.0847
59338	PLEKHA1	-0.0847
4147	MATN2	-0.0848
79875	THSD4	-0.0851
23654	PLXNB2	-0.0853
3745	KCNB1	-0.0854
145482	PTGR2	-0.0855
26020	LRP10	-0.0856
80333	KCNIP4	-0.0860
8934	RAB29	-0.0867
5175	PECAM1	-0.0868
8045	RASSF7	-0.0869
8417	STX7	-0.0870
283537	SLC46A3	-0.0870
54816	ZNF280D	-0.0870
57003	CCDC47	-0.0871
57337	SENP7	-0.0873
3570	IL6R	-0.0875
284434	NWD1	-0.0876
23765	IL17RA	-0.0877
10203	CALCRL	-0.0878
51765	STK26	-0.0878
402055	SRRD	-0.0881

9086	EIF1AY	-0.0883
103910	MYL12B	-0.0884
23212	RRS1	-0.0887
84465	MEGF11	-0.0889
205428	C3orf58	-0.0889
53358	SHC3	-0.0894
81035	COLEC12	-0.0895
3151	HMGN2	-0.0899
503542	SPRN	-0.0899
79887	PLBD1	-0.0900
1397	CRIP2	-0.0901
3187	HNRNPH1	-0.0904
65059	RAPH1	-0.0904
53616	ADAM22	-0.0907
177	AGER	-0.0907
55559	HAUS7	-0.0908
2176	FANCC	-0.0911
83638	C11orf68	-0.0912
84448	ABLIM2	-0.0914
56896	DPYSL5	-0.0917
10732	TCFL5	-0.0919
285172	FAM126B	-0.0923
5947	RBP1	-0.0924
5991	RFX3	-0.0925
9610	RIN1	-0.0926
3909	LAMA3	-0.0927
814	CAMK4	-0.0933
64940	STAG3L4	-0.0936
64208	POPDC3	-0.0937
3140	MR1	-0.0939
5935	RBM3	-0.0940
2224	FDPS	-0.0940
528	ATP6V1C1	-0.0940
65012	SLC26A10	-0.0941
159686	CFAP58	-0.0941
153	ADRB1	-0.0941
9194	SLC16A7	-0.0942
4548	MTR	-0.0942
192683	SCAMP5	-0.0947
6750	SST	-0.0948
650	BMP2	-0.0954
84034	EMILIN2	-0.0958
79891	ZNF671	-0.0961
79078	C1orf50	-0.0962
2823	GPM6A	-0.0962
52	ACP1	-0.0963
5542	PRB1	-0.0964
10003	NAALAD2	-0.0965
7433	VIPR1	-0.0966
55040	EPN3	-0.0966
22989	MYH15	-0.0966
3880	KRT19	-0.0968
4131	MAP1B	-0.0979
201514	ZNF584	-0.0980
10840	ALDH1L1	-0.0987

10633	RASL10A	-0.0990
56961	SHD	-0.0991
10335	MRVI1	-0.0995
1854	DUT	-0.0997
488	ATP2A2	-0.1001
23024	PDZRN3	-0.1005
817	CAMK2D	-0.1010
1131	CHRM3	-0.1013
79685	SAP30L	-0.1016
115353	LRRC42	-0.1021
6623	SNCG	-0.1021
55357	TBC1D2	-0.1027
340390	WDR97	-0.1030
6509	SLC1A4	-0.1031
5016	OVGP1	-0.1032
64599	GIGYF1	-0.1035
23460	ABCA6	-0.1038
9612	NCOR2	-0.1040
57190	SELENON	-0.1040
7532	YWHAG	-0.1041
79899	PRR5L	-0.1041
55532	SLC30A10	-0.1048
163081	ZNF567	-0.1048
79698	ZMAT4	-0.1048
253832	ZDHHC20	-0.1049
668	FOXL2	-0.1050
83539	CHST9	-0.1050
1632	ECI1	-0.1052
55922	NKRF	-0.1056
84221	SPATC1L	-0.1059
84079	ANKRD27	-0.1059
83648	FAM167A	-0.1060
5165	PDK3	-0.1061
4693	NDP	-0.1066
4500	MT1L	-0.1067
101	ADAM8	-0.1068
121793	TEX29	-0.1069
64080	RBKS	-0.1074
10797	MTHFD2	-0.1075
3779	KCNMB1	-0.1078
203286	ANKS6	-0.1078
11104	KATNA1	-0.1079
340419	RSPO2	-0.1079
1112	FOXN3	-0.1080
1630	DCC	-0.1082
51334	PRR16	-0.1083
25989	ULK3	-0.1083
1312	COMT	-0.1084
126626	GABPB2	-0.1087
2444	FRK	-0.1089
79057	PRRG3	-0.1091
149840	C20orf196	-0.1094
4774	NFIA	-0.1096
2274	FHL2	-0.1099
493861	EID3	-0.1100

28990	ASTE1	-0.1102
147015	DHRS13	-0.1107
56255	TMX4	-0.1108
10487	CAP1	-0.1109
51279	C1RL	-0.1111
23414	ZFPM2	-0.1113
84304	NUDT22	-0.1114
146433	IL34	-0.1114
2173	FABP7	-0.1115
440730	TRIM67	-0.1117
26061	HACL1	-0.1119
7402	UTRN	-0.1120
9472	AKAP6	-0.1120
91107	TRIM47	-0.1120
3667	IRS1	-0.1121
8898	MTMR2	-0.1122
128346	C1orf162	-0.1122
63970	TP53AIP1	-0.1123
51375	SNX7	-0.1126
83714	NRIP2	-0.1126
90134	KCNH7	-0.1126
112885	PHF21B	-0.1127
9943	OXSR1	-0.1127
55258	THNSL2	-0.1129
54551	MAGEL2	-0.1134
4802	NFYC	-0.1134
83445	GSG1	-0.1137
27163	NAAA	-0.1146
2110	ETFDH	-0.1146
3281	HSBP1	-0.1147
9945	GFPT2	-0.1147
79722	ANKRD55	-0.1149
200035	NUDT17	-0.1149
2550	GABBR1	-0.1151
1806	DPYD	-0.1152
84936	ZFYVE19	-0.1153
6470	SHMT1	-0.1154
25822	DNAJB5	-0.1154
7122	CLDN5	-0.1155
219670	ENKUR	-0.1155
641700	ECSCR	-0.1159
3131	HLF	-0.1159
283576	ZDHHC22	-0.1162
57404	CYP20A1	-0.1166
9056	SLC7A7	-0.1167
10981	RAB32	-0.1168
7469	NELFA	-0.1170
196403	DTX3	-0.1170
146223	CMTM4	-0.1174
3429	IFI27	-0.1178
124093	CCDC78	-0.1178
29886	SNX8	-0.1182
81576	CCDC130	-0.1182
51226	COPZ2	-0.1182
9070	ASH2L	-0.1182

56936	CCDC177	-0.1187
146556	C16orf89	-0.1187
10039	PARP3	-0.1188
175	AGA	-0.1192
64377	CHST8	-0.1192
3612	IMPA1	-0.1193
5621	PRNP	-0.1193
57728	WDR19	-0.1196
8497	PPFIA4	-0.1197
55379	LRRC59	-0.1199
199800	ADM5	-0.1200
79693	YRDC	-0.1202
84988	PPP1R16A	-0.1203
108	ADCY2	-0.1204
83439	TCF7L1	-0.1213
10248	POP7	-0.1213
163154	PRR22	-0.1213
64170	CARD9	-0.1214
654790	PCP4L1	-0.1216
64116	SLC39A8	-0.1217
3984	LIMK1	-0.1217
22800	RRAS2	-0.1220
84617	TUBB6	-0.1224
137872	ADHFE1	-0.1226
1501	CTNND2	-0.1229
56521	DNAJC12	-0.1229
10683	DLL3	-0.1229
348093	RBPMS2	-0.1233
582	BBS1	-0.1233
114879	OSBPL5	-0.1233
6450	SH3BGR	-0.1234
26145	IRF2BP1	-0.1236
6905	TBCE	-0.1237
11019	LIAS	-0.1238
114548	NLRP3	-0.1238
10325	RRAGB	-0.1238
6586	SLIT3	-0.1239
10724	MGEA5	-0.1240
55540	IL17RB	-0.1240
84922	FIZ1	-0.1244
145567	TTC7B	-0.1245
51454	GULP1	-0.1245
27072	VPS41	-0.1249
10011	SRA1	-0.1253
91851	CHRDL1	-0.1253
4489	MT1A	-0.1256
58512	DLGAP3	-0.1256
7205	TRIP6	-0.1257
55907	CMAS	-0.1257
5587	PRKD1	-0.1259
8263	F8A1	-0.1259
134548	SOWAHA	-0.1260
3964	LGALS8	-0.1264
353149	TBC1D26	-0.1266
5961	PRPH2	-0.1267

84812	PLCD4	-0.1267
6560	SLC12A4	-0.1269
9607	CARTPT	-0.1270
1230	CCR1	-0.1272
2681	GGTA1P	-0.1274
2200	FBN1	-0.1275
3689	ITGB2	-0.1276
9570	GOSR2	-0.1276
1728	NQO1	-0.1279
10901	DHRS4	-0.1284
643037	C11orf97	-0.1286
7101	NR2E1	-0.1291
54622	ARL15	-0.1292
23539	SLC16A8	-0.1292
1755	DMBT1	-0.1292
387496	RASL11A	-0.1293
2697	GJA1	-0.1293
148808	MFSD4A	-0.1294
55273	TMEM100	-0.1295
8277	TKTL1	-0.1295
4931	NVL	-0.1295
114789	SLC25A25	-0.1297
4354	MPP1	-0.1300
146705	TEPSIN	-0.1301
152940	C4orf45	-0.1301
64089	SNX16	-0.1301
79918	SETD6	-0.1302
2567	GABRG3	-0.1306
10206	TRIM13	-0.1307
201780	SLC10A4	-0.1308
2054	STX2	-0.1313
2044	EPHA5	-0.1314
80144	FRAS1	-0.1316
56180	MOSPD1	-0.1316
87178	PNPT1	-0.1319
81894	SLC25A28	-0.1319
9064	MAP3K6	-0.1319
3695	ITGB7	-0.1321
80853	KDM7A	-0.1322
140578	CHODL	-0.1324
64061	TSPYL2	-0.1324
56126	PCDHB10	-0.1328
55786	ZNF415	-0.1328
64927	TTC23	-0.1328
367	AR	-0.1329
90806	ANGEL2	-0.1330
26031	OSBPL3	-0.1332
11061	CNMD	-0.1334
90865	IL33	-0.1336
8736	MYOM1	-0.1336
117245	HRASLS5	-0.1336
10466	COG5	-0.1338
4792	NFKBIA	-0.1340
6197	RPS6KA3	-0.1340
22809	ATF5	-0.1340

134957	STXBP5	-0.1343
1757	SARDH	-0.1345
100129792	CCDC152	-0.1346
4636	MYL5	-0.1346
5468	PPARG	-0.1347
285464	CRIPAK	-0.1348
55920	RCC2	-0.1348
55315	SLC29A3	-0.1354
6646	SOAT1	-0.1354
91522	COL23A1	-0.1356
64855	FAM129B	-0.1357
5348	FXYD1	-0.1357
28231	SLCO4A1	-0.1359
401994	OR14I1	-0.1359
91409	CCDC74B	-0.1360
56479	KCNQ5	-0.1361
2670	GFAP	-0.1361
5480	PPIC	-0.1363
55079	FEZF2	-0.1364
7280	TUBB2A	-0.1364
2103	ESRRB	-0.1365
93663	ARHGAP18	-0.1366
57161	PELI2	-0.1368
2535	FZD2	-0.1370
7051	TGM1	-0.1370
204	AK2	-0.1370
8519	IFITM1	-0.1372
2644	GCHFR	-0.1376
9465	AKAP7	-0.1376
80781	COL18A1	-0.1376
7159	TP53BP2	-0.1379
10793	ZNF273	-0.1381
3419	IDH3A	-0.1383
3679	ITGA7	-0.1384
83541	FAM110A	-0.1386
29904	EEF2K	-0.1387
54769	DIRAS2	-0.1387
25829	TMEM184B	-0.1389
25874	MPC2	-0.1390
282996	RBM20	-0.1391
2730	GCLM	-0.1391
79090	TRAPPC6A	-0.1392
5606	MAP2K3	-0.1393
4495	MT1G	-0.1394
9118	INA	-0.1395
55835	CENPJ	-0.1396
6039	RNASE6	-0.1397
3183	HNRNPC	-0.1398
11010	GLIPR1	-0.1403
2262	GPC5	-0.1405
51650	MRPS33	-0.1405
5806	PTX3	-0.1411
5306	PITPNA	-0.1418
407738	FAM19A1	-0.1421
79805	VASH2	-0.1422

55268	ECHDC2	-0.1423
55320	MIS18BP1	-0.1423
132160	PPM1M	-0.1424
3931	LCAT	-0.1428
64067	NPAS3	-0.1429
116328	C8orf34	-0.1431
8913	CACNA1G	-0.1431
3613	IMPA2	-0.1436
9071	CLDN10	-0.1437
255426	RASGEF1C	-0.1437
58499	ZNF462	-0.1440
2843	GPR20	-0.1441
55312	RFK	-0.1441
388327	C17orf100	-0.1442
966	CD59	-0.1446
221154	MICU2	-0.1447
5021	OXTR	-0.1448
54112	GPR88	-0.1449
54716	SLC6A20	-0.1452
260293	CYP4X1	-0.1455
80301	PLEKHO2	-0.1455
200879	LIPH	-0.1455
432	ASGR1	-0.1457
2247	FGF2	-0.1458
140733	MACROD2	-0.1458
57205	ATP10D	-0.1459
891	CCNB1	-0.1461
1571	CYP2E1	-0.1465
1768	DNAH6	-0.1468
55160	ARHGEF10L	-0.1470
23409	SIRT4	-0.1470
139728	PNCK	-0.1471
9406	ZRANB2	-0.1472
26577	PCOLCE2	-0.1475
22954	TRIM32	-0.1475
56971	CEACAM19	-0.1477
63027	SLC22A23	-0.1477
23250	ATP11A	-0.1480
268	AMH	-0.1480
10220	GDF11	-0.1480
79600	TCTN1	-0.1486
8601	RGS20	-0.1489
94235	GNG8	-0.1489
387066	SNHG5	-0.1490
5366	PMAIP1	-0.1493
3747	KCNC2	-0.1494
409	ARRB2	-0.1494
2669	GEM	-0.1495
25893	TRIM58	-0.1495
387357	THEMIS	-0.1498
475	ATOX1	-0.1501
11259	FILIP1L	-0.1502
642658	SCX	-0.1504
865	CBFB	-0.1504
4892	NRAP	-0.1505

50484	RRM2B	-0.1507
57512	GPR158	-0.1511
83692	CD99L2	-0.1513
8774	NAPG	-0.1513
64895	PAPOLG	-0.1515
64760	FAM160B2	-0.1516
54932	EXD3	-0.1517
63827	BCAN	-0.1518
5452	POU2F2	-0.1520
10384	BTN3A3	-0.1521
57214	CEMIP	-0.1523
55840	EAF2	-0.1525
8718	TNFRSF25	-0.1526
355	FAS	-0.1526
9013	TAF1C	-0.1527
8174	MADCAM1	-0.1528
7114	TMSB4X	-0.1529
3315	HSPB1	-0.1529
348094	ANKDD1A	-0.1531
81575	APOLD1	-0.1532
8682	PEA15	-0.1535
84002	B3GNT5	-0.1539
5983	RFC3	-0.1540
11001	SLC27A2	-0.1542
163486	DENND1B	-0.1543
80326	WNT10A	-0.1545
7125	TNNC2	-0.1550
118430	MUCL1	-0.1550
23043	TNIK	-0.1551
29109	FHOD1	-0.1553
170261	ZCCHC12	-0.1554
79705	LRRK1	-0.1558
4026	LPP	-0.1560
54976	C20orf27	-0.1563
158038	LINGO2	-0.1563
2259	FGF14	-0.1563
5450	POU2AF1	-0.1564
3361	HTR5A	-0.1565
154	ADRB2	-0.1567
9095	TBX19	-0.1571
2888	GRB14	-0.1571
154807	VKORC1L1	-0.1575
1263	PLK3	-0.1577
56172	ANKH	-0.1578
85369	STRIP1	-0.1581
90161	HS6ST2	-0.1583
29062	WDR91	-0.1585
56851	EMC7	-0.1587
85478	CCDC65	-0.1587
79901	CYBRD1	-0.1587
3109	HLA-DMB	-0.1588
56963	RGMA	-0.1588
200186	CRTC2	-0.1591
2210	FCGR1B	-0.1593
91977	MYOZ3	-0.1598

143689	PIWIL4	-0.1599
390616	ANKRD34C	-0.1601
3925	STMN1	-0.1605
9852	EPM2AIP1	-0.1607
9104	RGN	-0.1608
55800	SCN3B	-0.1609
2620	GAS2	-0.1612
952	CD38	-0.1613
7037	TFRC	-0.1614
91683	SYT12	-0.1615
2897	GRIK1	-0.1618
115827	RAB3C	-0.1619
1523	CUX1	-0.1620
23166	STAB1	-0.1625
27229	TUBGCP4	-0.1629
54602	NDFIP2	-0.1630
284	ANGPT1	-0.1631
1849	DUSP7	-0.1632
10605	PAIP1	-0.1633
83546	RTBDN	-0.1634
284521	OR2L13	-0.1634
7726	TRIM26	-0.1634
23506	BICRAL	-0.1635
130827	TMEM182	-0.1636
240	ALOX5	-0.1643
148932	MOB3C	-0.1643
81577	GFOD2	-0.1644
23379	ICE1	-0.1644
92162	TMEM88	-0.1644
220296	HEPACAM	-0.1645
5771	PTPN2	-0.1646
55617	TASP1	-0.1650
84899	TMTC4	-0.1650
5980	REV3L	-0.1651
79930	DOK3	-0.1652
148014	TTC9B	-0.1655
30008	EFEMP2	-0.1655
25788	RAD54B	-0.1655
151393	RMDN2	-0.1656
57732	ZFYVE28	-0.1658
4616	GADD45B	-0.1658
124842	TMEM132E	-0.1660
389119	FAM212A	-0.1661
7083	TK1	-0.1662
79369	B3GNT4	-0.1664
6594	SMARCA1	-0.1666
124997	WDR81	-0.1667
114757	CYGB	-0.1670
9702	CEP57	-0.1670
64747	MFSD1	-0.1670
283284	IGSF22	-0.1673
27092	CACNG4	-0.1673
23408	SIRT5	-0.1674
255631	COL24A1	-0.1674
2114	ETS2	-0.1677

1272	CNTN1	-0.1678
8864	PER2	-0.1681
64288	ZSCAN31	-0.1684
285755	PPIL6	-0.1686
23607	CD2AP	-0.1687
55365	TMEM176A	-0.1687
869	CBLN1	-0.1688
79652	TMEM204	-0.1693
3995	FADS3	-0.1693
27010	TPK1	-0.1694
51676	ASB2	-0.1696
4013	VWA5A	-0.1696
10048	RANBP9	-0.1698
55187	VPS13D	-0.1698
5547	PRCP	-0.1698
119032	BORCS7	-0.1701
55680	RUFY2	-0.1701
9172	MYOM2	-0.1701
7096	TLR1	-0.1703
85477	SCIN	-0.1705
7771	ZNF112	-0.1705
9920	KBTBD11	-0.1706
1880	GPR183	-0.1710
60370	AVPI1	-0.1711
11187	PKP3	-0.1711
23255	MTCL1	-0.1713
11170	FAM107A	-0.1714
114781	BTBD9	-0.1716
5550	PREP	-0.1720
6134	RPL10	-0.1721
9937	DCLRE1A	-0.1724
51564	HDAC7	-0.1724
9854	C2CD2L	-0.1734
1136	CHRNA3	-0.1736
4238	MFAP3	-0.1737
8613	PLPP3	-0.1742
53942	CNTN5	-0.1742
2745	GLRX	-0.1743
9645	MICAL2	-0.1744
3706	ITPKA	-0.1744
57519	STARD9	-0.1745
860	RUNX2	-0.1746
1008	CDH10	-0.1747
57636	ARHGAP23	-0.1752
4023	LPL	-0.1758
84684	INSM2	-0.1758
58516	SINHCAF	-0.1762
2596	GAP43	-0.1769
9069	CLDN12	-0.1770
10615	SPAG5	-0.1773
348	APOE	-0.1773
414152	C10orf105	-0.1773
433	ASGR2	-0.1774
399947	C11orf87	-0.1774
3400	ID4	-0.1778

51559	NT5DC3	-0.1780
150967	LINC01963	-0.1783
27000	DNAJC2	-0.1785
51343	FZR1	-0.1787
147339	C18orf25	-0.1787
79085	SLC25A23	-0.1788
1949	EFNB3	-0.1793
80017	DGLUCY	-0.1794
83593	RASSF5	-0.1795
168002	DACT2	-0.1796
10040	TOM1L1	-0.1796
1643	DDB2	-0.1797
3671	ISLR	-0.1798
60626	RIC8A	-0.1798
283209	PGM2L1	-0.1803
3684	ITGAM	-0.1806
2565	GABRG1	-0.1807
5293	PIK3CD	-0.1809
151516	ASPRV1	-0.1811
8863	PER3	-0.1813
11138	TBC1D8	-0.1814
9644	SH3PXD2A	-0.1814
84937	ZNRF1	-0.1817
4647	MYO7A	-0.1819
118429	ANTXR2	-0.1823
23395	LARS2	-0.1827
80176	SPSB1	-0.1827
286140	RNF5P1	-0.1829
127602	DNAH14	-0.1829
219854	TMEM218	-0.1829
23620	NTSR2	-0.1829
10484	SEC23A	-0.1833
57060	PCBP4	-0.1835
100270746	LOC100270746	-0.1835
1363	CPE	-0.1837
7477	WNT7B	-0.1838
3005	H1F0	-0.1843
22941	SHANK2	-0.1843
388341	LRRC75A	-0.1846
3157	HMGCS1	-0.1847
55343	SLC35C1	-0.1848
494470	RNF165	-0.1848
100289230	LOC100289230	-0.1852
7766	ZNF223	-0.1852
9256	TSPOAP1	-0.1855
7781	SLC30A3	-0.1856
64172	OSGEPL1	-0.1861
9450	LY86	-0.1861
19	ABCA1	-0.1861
401494	HACD4	-0.1862
8425	LTBP4	-0.1865
406	ARNTL	-0.1868
7126	TNFAIP1	-0.1871
80271	ITPKC	-0.1872
11326	VSIG4	-0.1873

672	BRCA1	-0.1877
399744	LINC00999	-0.1879
7936	NELFE	-0.1880
285368	PRRT3	-0.1882
63901	FAM111A	-0.1883
203197	TMEM268	-0.1884
23609	MKRN2	-0.1885
2533	FYB1	-0.1885
84134	TOMM40L	-0.1886
29954	POMT2	-0.1887
51170	HSD17B11	-0.1888
10368	CACNG3	-0.1890
94097	SFXN5	-0.1891
3949	LDLR	-0.1892
57522	SRGAP1	-0.1893
26063	DECR2	-0.1893
4209	MEF2D	-0.1894
11093	ADAMTS13	-0.1899
286183	NKAIN3	-0.1899
54757	FAM20A	-0.1900
126695	KDF1	-0.1900
23504	RIMBP2	-0.1901
9338	TCEAL1	-0.1903
79027	ZNF655	-0.1905
167838	TXLNB	-0.1906
2947	GSTM3	-0.1906
5603	MAPK13	-0.1907
29035	C16orf72	-0.1907
23111	SPART	-0.1908
80237	ELL3	-0.1911
5176	SERPINF1	-0.1912
728448	PPIEL	-0.1915
2898	GRIK2	-0.1915
201191	SAMD14	-0.1916
64333	ARHGAP9	-0.1916
10063	COX17	-0.1918
136853	SSC4D	-0.1918
30846	EHD2	-0.1918
26050	SLITRK5	-0.1918
80210	ARMC9	-0.1919
79012	CAMKV	-0.1923
50649	ARHGEF4	-0.1930
5311	PKD2	-0.1931
6513	SLC2A1	-0.1932
53335	BCL11A	-0.1932
25834	MGAT4C	-0.1932
29969	MDFIC	-0.1934
200728	TMEM17	-0.1934
440097	DBX2	-0.1936
1807	DPYS	-0.1937
161	AP2A2	-0.1942
64854	USP46	-0.1943
118813	ZFYVE27	-0.1943
404217	CTXN1	-0.1945
3059	HCLS1	-0.1946

261734	NPHP4	-0.1947
152273	FGD5	-0.1947
9828	ARHGEF17	-0.1947
80212	CCDC92	-0.1949
5908	RAP1B	-0.1951
3547	IGSF1	-0.1951
56165	TDRD1	-0.1953
2820	GPD2	-0.1958
340719	NANOS1	-0.1959
389084	SNORC	-0.1961
25758	KIAA1549L	-0.1961
56616	DIABLO	-0.1963
259217	HSPA12A	-0.1965
2587	GALR1	-0.1965
85301	COL27A1	-0.1967
3872	KRT17	-0.1969
9665	MARF1	-0.1972
2295	FOXF2	-0.1976
3768	KCNJ12	-0.1977
84455	EFCAB7	-0.1978
80213	TM2D3	-0.1979
3082	HGF	-0.1982
57713	SFMBT2	-0.1983
2731	GLDC	-0.1983
51104	ABHD17B	-0.1983
9175	MAP3K13	-0.1984
4493	MT1E	-0.1987
373156	GSTK1	-0.1987
54800	KLHL24	-0.1989
7799	PRDM2	-0.1989
441666	LOC441666	-0.1990
90355	C5orf30	-0.1991
55509	BATF3	-0.1991
6284	S100A13	-0.1998
401498	TMEM215	-0.1998
10302	SNAPC5	-0.1999
3269	HRH1	-0.1999
64132	XYLT2	-0.2002
148979	GLIS1	-0.2002
1536	CYBB	-0.2004
4613	MYCN	-0.2004
221303	FAM162B	-0.2006
9976	CLEC2B	-0.2006
1300	COL10A1	-0.2008
6620	SNCB	-0.2009
1268	CNR1	-0.2009
10279	PRSS16	-0.2011
3175	ONECUT1	-0.2014
10555	AGPAT2	-0.2015
3889	KRT83	-0.2016
23363	OBSL1	-0.2016
118812	MORN4	-0.2017
55186	SLC25A36	-0.2021
6949	TCOF1	-0.2023
3781	KCNN2	-0.2025

9262	STK17B	-0.2025
642938	FAM196A	-0.2028
221927	BRAT1	-0.2030
7091	TLE4	-0.2034
4884	NPTX1	-0.2036
7852	CXCR4	-0.2037
127733	UBXN10	-0.2038
84223	IQCG	-0.2039
54910	SEMA4C	-0.2039
23467	NPTXR	-0.2039
112609	MRAP2	-0.2043
7862	BRPF1	-0.2043
3127	HLA-DRB5	-0.2044
6136	RPL12	-0.2045
3354	HTR1E	-0.2047
6866	TAC3	-0.2047
7291	TWIST1	-0.2053
81849	ST6GALNAC5	-0.2053
115817	DHRS1	-0.2057
7378	UPP1	-0.2057
83547	RILP	-0.2060
249	ALPL	-0.2061
197187	SNAI3-AS1	-0.2062
11251	PTGDR2	-0.2062
3755	KCNG1	-0.2063
9231	DLG5	-0.2066
165215	FAM171B	-0.2069
54361	WNT4	-0.2070
64412	GZF1	-0.2070
5570	PKIB	-0.2074
6419	SETMAR	-0.2076
9991	PTBP3	-0.2077
7732	RNF112	-0.2077
51161	C3orf18	-0.2078
5979	RET	-0.2084
10247	RIDA	-0.2085
8303	SNN	-0.2085
163702	IFNLR1	-0.2085
2932	GSK3B	-0.2087
2632	GBE1	-0.2088
9542	NRG2	-0.2088
57224	NHSL1	-0.2089
220164	DOK6	-0.2090
64407	RGS18	-0.2094
4254	KITLG	-0.2097
154043	CNKSR3	-0.2098
79785	RERGL	-0.2099
728215	FAM155A	-0.2100
166614	DCLK2	-0.2103
27079	RPUSD2	-0.2105
92689	FAM114A1	-0.2105
7482	WNT2B	-0.2106
8325	FZD8	-0.2106
5091	PC	-0.2106
8911	CACNA1I	-0.2107

1636	ACE	-0.2109
84759	PCGF1	-0.2110
133	ADM	-0.2115
8704	B4GALT2	-0.2115
2268	FGR	-0.2115
23471	TRAM1	-0.2116
4043	LRPAP1	-0.2118
349667	RTN4RL2	-0.2118
9209	LRRFIP2	-0.2119
5689	PSMB1	-0.2119
29095	ORMDL2	-0.2121
203859	ANO5	-0.2122
56243	KIAA1217	-0.2123
56133	PCDHB2	-0.2123
400120	SERTM1	-0.2125
55603	FAM46A	-0.2129
205860	TRIML2	-0.2131
51296	SLC15A3	-0.2131
51477	ISYNA1	-0.2131
51271	UBAP1	-0.2132
3912	LAMB1	-0.2133
29995	LMCD1	-0.2134
3111	HLA-DOA	-0.2134
10207	PATJ	-0.2137
146439	BICDL2	-0.2140
256435	ST6GALNAC3	-0.2141
57616	TSHZ3	-0.2143
10309	CCNO	-0.2143
9362	CPNE6	-0.2146
4293	MAP3K9	-0.2147
23673	STX12	-0.2147
5631	PRPS1	-0.2147
22875	ENPP4	-0.2147
55654	TMEM127	-0.2148
1	A1BG	-0.2148
4149	MAX	-0.2148
133418	EMB	-0.2148
2840	GPR17	-0.2152
57452	GALNT16	-0.2152
493856	CISD2	-0.2153
57605	PITPNM2	-0.2154
25934	NIPSNAP3A	-0.2155
9550	ATP6V1G1	-0.2157
6578	SLCO2A1	-0.2159
220323	OAF	-0.2161
2202	EFEMP1	-0.2161
8516	ITGA8	-0.2163
128344	PIFO	-0.2167
79161	TMEM243	-0.2168
10611	PDLIM5	-0.2172
57761	TRIB3	-0.2172
114132	SIGLEC11	-0.2172
128553	TSHZ2	-0.2172
79815	NIPAL2	-0.2181
64093	SMOC1	-0.2181

80059	LRRTM4	-0.2183
80184	CEP290	-0.2185
217	ALDH2	-0.2192
9189	ZBED1	-0.2192
25854	FAM149A	-0.2195
81033	KCNH6	-0.2195
56944	OLFML3	-0.2196
51393	TRPV2	-0.2199
8452	CUL3	-0.2206
753	LDLRAD4	-0.2207
121053	C12orf45	-0.2211
5987	TRIM27	-0.2213
56937	PMEPA1	-0.2214
29078	NDUFAF4	-0.2215
9712	USP6NL	-0.2216
1006	CDH8	-0.2216
302	ANXA2	-0.2217
1374	CPT1A	-0.2218
57586	SYT13	-0.2220
93081	TEX30	-0.2221
4257	MGST1	-0.2222
6332	SCN7A	-0.2223
64764	CREB3L2	-0.2223
10795	ZNF268	-0.2224
9589	WTAP	-0.2224
171024	SYNPO2	-0.2225
56270	WDR45B	-0.2226
57128	LYRM4	-0.2227
138716	RPP25L	-0.2228
3028	HSD17B10	-0.2231
23336	SYNM	-0.2232
83699	SH3BGRL2	-0.2234
148252	DIRAS1	-0.2234
92241	RCSD1	-0.2236
131375	LYZL4	-0.2237
701	BUB1B	-0.2238
23625	FAM89B	-0.2238
23034	SAMD4A	-0.2240
81620	CDT1	-0.2240
83733	SLC25A18	-0.2241
375057	STUM	-0.2247
26011	TENM4	-0.2250
55805	LRP2BP	-0.2251
150365	MEI1	-0.2252
129049	SGSM1	-0.2254
4072	EPCAM	-0.2255
8086	AAAS	-0.2255
92610	TIFA	-0.2256
9832	JAKMIP2	-0.2256
5600	MAPK11	-0.2257
57623	ZFAT	-0.2258
5649	RELN	-0.2259
10449	ACAA2	-0.2262
143098	MPP7	-0.2262
90550	MCU	-0.2263

642852	LOC642852	-0.2267
81624	DIAPH3	-0.2268
54516	MTRF1L	-0.2268
144404	TMEM120B	-0.2269
9411	ARHGAP29	-0.2269
57526	PCDH19	-0.2271
8829	NRP1	-0.2271
140576	S100A16	-0.2272
6752	SSTR2	-0.2272
10519	CIB1	-0.2273
23456	ABCB10	-0.2274
602	BCL3	-0.2275
84699	CREB3L3	-0.2275
22846	VASH1	-0.2276
7105	TSPAN6	-0.2281
642	BLMH	-0.2284
90525	SHF	-0.2286
55707	NECAP2	-0.2289
9925	ZBTB5	-0.2289
5646	PRSS3	-0.2289
132671	SPATA18	-0.2292
220	ALDH1A3	-0.2293
56888	KCMF1	-0.2298
150864	FAM117B	-0.2298
8996	NOL3	-0.2299
89876	MAATS1	-0.2300
199720	GGN	-0.2303
79955	PDZD7	-0.2304
26009	ZZZ3	-0.2306
283316	CD163L1	-0.2314
2956	MSH6	-0.2314
8986	RPS6KA4	-0.2318
65249	ZSWIM4	-0.2319
9715	FAM131B	-0.2321
60493	FASTKD5	-0.2324
134265	AFAP1L1	-0.2326
3776	KCNK2	-0.2328
55208	DCUN1D2	-0.2328
55101	DMAC2	-0.2332
144363	ETFRF1	-0.2333
7692	ZNF133	-0.2335
389941	C1QL3	-0.2336
27153	ZNF777	-0.2336
64411	ARAP3	-0.2337
6697	SPR	-0.2341
23657	SLC7A11	-0.2342
23475	QPRT	-0.2343
51110	LACTB2	-0.2344
26160	IFT172	-0.2345
793	CALB1	-0.2347
33	ACADL	-0.2347
8650	NUMB	-0.2348
27040	LAT	-0.2351
63916	ELMO2	-0.2352
51299	NRN1	-0.2352

55068	ENOX1	-0.2353
8323	FZD6	-0.2355
79017	GGCT	-0.2356
80148	PQLC1	-0.2361
23219	FBXO28	-0.2362
2922	GRP	-0.2364
80179	MYO19	-0.2365
10459	MAD2L2	-0.2366
9512	PMPCB	-0.2367
54463	RETREG1	-0.2368
9628	RGS6	-0.2368
94160	ABCC12	-0.2368
196047	EMX2OS	-0.2370
4603	MYBL1	-0.2374
51285	RASL12	-0.2375
64850	ETNPPL	-0.2375
9404	LPXN	-0.2375
165	AEBP1	-0.2376
1029	CDKN2A	-0.2377
51083	GAL	-0.2380
92106	OXNAD1	-0.2381
59342	SCPEP1	-0.2383
6585	SLIT1	-0.2384
646424	SPINK8	-0.2386
22883	CLSTN1	-0.2388
10620	ARID3B	-0.2394
83746	L3MBTL2	-0.2394
5080	PAX6	-0.2397
171425	CLYBL	-0.2399
84878	ZBTB45	-0.2401
4820	NKTR	-0.2401
90668	CARMIL3	-0.2402
56998	CTNNBIP1	-0.2403
171019	ADAMTS19	-0.2403
51390	AIG1	-0.2404
51105	PHF20L1	-0.2404
4599	MX1	-0.2404
113622	ADPRHL1	-0.2405
23704	KCNE4	-0.2409
150290	DUSP18	-0.2411
57480	PLEKHG1	-0.2419
119391	GSTO2	-0.2422
9055	PRC1	-0.2422
6540	SLC6A13	-0.2423
64794	DDX31	-0.2423
7849	PAX8	-0.2423
51138	COPS4	-0.2424
400745	SH2D5	-0.2427
1421	CRYGD	-0.2427
147495	APCDD1	-0.2428
5321	PLA2G4A	-0.2432
151056	PLB1	-0.2437
3176	HNMT	-0.2437
8717	TRADD	-0.2439
10585	POMT1	-0.2440

9914	ATP2C2	-0.2440
415116	PIM3	-0.2443
54894	RNF43	-0.2444
3396	MRPL58	-0.2445
8630	HSD17B6	-0.2445
221914	GPC2	-0.2447
460	ASTN1	-0.2449
2937	GSS	-0.2453
79991	STN1	-0.2454
92370	PXYLP1	-0.2456
26576	SRPK3	-0.2458
22874	PLEKHA6	-0.2459
8001	GLRA3	-0.2463
4241	MELTF	-0.2463
1302	COL11A2	-0.2463
26052	DNM3	-0.2465
1912	PHC2	-0.2466
7067	THRA	-0.2466
90634	N4BP2L1	-0.2472
51693	TRAPPC2L	-0.2472
55027	HEATR3	-0.2473
55254	TMEM39A	-0.2475
128486	FITM2	-0.2475
5874	RAB27B	-0.2476
10252	SPRY1	-0.2476
64236	PDLIM2	-0.2476
23163	GGA3	-0.2477
84811	BUD13	-0.2477
1002	CDH4	-0.2479
205327	C2orf69	-0.2481
7743	ZNF189	-0.2481
403341	ZBTB34	-0.2485
23026	MYO16	-0.2491
8936	WASF1	-0.2492
55885	LMO3	-0.2493
129080	EMID1	-0.2494
80216	ALPK1	-0.2495
1491	CTH	-0.2497
6282	S100A11	-0.2499
1007	CDH9	-0.2499
54660	PCDHB18P	-0.2501
138311	FAM69B	-0.2503
57449	PLEKHG5	-0.2506
29117	BRD7	-0.2506
79156	PLEKHF1	-0.2507
59350	RXFP1	-0.2509
388581	C1QTNF12	-0.2513
4828	NMB	-0.2513
90673	PPP1R3E	-0.2515
10535	RNASEH2A	-0.2515
3939	LDHA	-0.2516
1608	DGKG	-0.2516
256536	TCERG1L	-0.2517
23269	MGA	-0.2517
4852	NPY	-0.2518

9847	C2CD5	-0.2518
207107	SFTA1P	-0.2520
55006	TRMT61B	-0.2520
132332	TMEM155	-0.2523
653121	ZBTB8A	-0.2524
25941	TPGS2	-0.2528
23705	CADM1	-0.2528
2786	GNG4	-0.2530
26033	ATRNL1	-0.2530
403	ARL3	-0.2533
9826	ARHGEF11	-0.2533
84080	ENKD1	-0.2533
64410	KLHL25	-0.2535
1104	RCC1	-0.2538
55277	FGGY	-0.2540
29126	CD274	-0.2540
256643	BCLAF3	-0.2541
4090	SMAD5	-0.2541
2222	FDFT1	-0.2543
79632	FAM184A	-0.2543
5596	MAPK4	-0.2544
6573	SLC19A1	-0.2544
203522	INTS6L	-0.2545
84698	CAPS2	-0.2545
1488	CTBP2	-0.2547
3299	HSF4	-0.2548
577	ADGRB3	-0.2549
285362	SUMF1	-0.2555
5195	PEX14	-0.2556
54556	ING3	-0.2562
5580	PRKCD	-0.2562
4259	MGST3	-0.2563
395	ARHGAP6	-0.2563
54762	GRAMD1C	-0.2563
5396	PRRX1	-0.2569
151887	CCDC80	-0.2573
80146	UXS1	-0.2577
51751	HIGD1B	-0.2578
5920	RARRES3	-0.2583
121227	LRIG3	-0.2583
84858	ZNF503	-0.2584
51046	ST8SIA3	-0.2587
113146	AHNAK2	-0.2589
3303	HSPA1A	-0.2590
7295	TXN	-0.2592
81034	SLC25A32	-0.2592
10560	SLC19A2	-0.2593
79713	IGFLR1	-0.2594
64776	C11orf1	-0.2601
1607	DGKB	-0.2604
23288	IQCE	-0.2612
54851	ANKRD49	-0.2615
10783	NEK6	-0.2616
1602	DACH1	-0.2620
7170	TPM3	-0.2623

2332	FMR1	-0.2626
23089	PEG10	-0.2627
5031	P2RY6	-0.2628
2014	EMP3	-0.2629
192669	AGO3	-0.2632
7162	TPBG	-0.2633
126520	PLK5	-0.2634
28992	MACROD1	-0.2637
393	ARHGAP4	-0.2638
9473	THEMIS2	-0.2640
340481	ZDHHC21	-0.2647
6423	SFRP2	-0.2651
136	ADORA2B	-0.2653
10974	ADIRF	-0.2653
2852	GPER1	-0.2656
90293	KLHL13	-0.2657
8317	CDC7	-0.2659
7462	LAT2	-0.2661
887	CCKBR	-0.2661
8324	FZD7	-0.2663
51339	DACT1	-0.2668
83986	FAM234A	-0.2673
100129583	FAM47E	-0.2673
81669	CCNL2	-0.2673
6610	SMPD2	-0.2674
5740	PTGIS	-0.2674
8715	NOL4	-0.2675
59307	SIGIRR	-0.2675
56955	MEPE	-0.2677
4858	NOVA2	-0.2677
51705	EMCN	-0.2680
3674	ITGA2B	-0.2681
2914	GRM4	-0.2681
80816	ASXL3	-0.2683
84152	PPP1R1B	-0.2685
170850	KCNG3	-0.2686
5803	PTPRZ1	-0.2686
10549	PRDX4	-0.2693
84189	SLITRK6	-0.2694
10950	BTG3	-0.2695
373863	DND1	-0.2695
54558	SPATA6	-0.2698
221074	SLC39A12	-0.2699
5172	SLC26A4	-0.2700
404550	C16orf74	-0.2702
1004	CDH6	-0.2703
128977	C22orf39	-0.2703
3566	IL4R	-0.2706
8805	TRIM24	-0.2709
148641	SLC35F3	-0.2709
54922	RASIP1	-0.2714
10125	RASGRP1	-0.2716
388815	MIR99AHG	-0.2719
65217	PCDH15	-0.2719
8418	CMAHP	-0.2719

64859	NABP1	-0.2722
5208	PFKFB2	-0.2723
7106	TSPAN4	-0.2727
9537	TP53I11	-0.2728
8424	BBOX1	-0.2731
51715	RAB23	-0.2733
9254	CACNA2D2	-0.2735
27253	PCDH17	-0.2736
84814	PLPP7	-0.2736
6860	SYT4	-0.2736
7062	TCHH	-0.2744
10212	DDX39A	-0.2745
55113	XKR8	-0.2746
4935	GPR143	-0.2746
26166	RGS22	-0.2747
163732	CITED4	-0.2748
758	MPPED1	-0.2749
8569	MKNK1	-0.2749
10669	CGREF1	-0.2751
55733	HHAT	-0.2753
51764	GNG13	-0.2753
59277	NTN4	-0.2760
147906	DACT3	-0.2762
664	BNIP3	-0.2767
203523	ZNF449	-0.2767
6247	RS1	-0.2767
91695	RRP7BP	-0.2771
51606	ATP6V1H	-0.2772
5744	PTHLH	-0.2775
22821	RASA3	-0.2777
55846	ITFG2	-0.2779
348013	TMEM255B	-0.2781
5893	RAD52	-0.2782
9267	CYTH1	-0.2785
5436	POLR2G	-0.2788
1734	DIO2	-0.2791
116238	TLCD1	-0.2794
94121	SYTL4	-0.2794
1729	DIAPH1	-0.2794
1690	COCH	-0.2796
79817	MOB3B	-0.2800
10752	CHL1	-0.2801
6234	RPS28	-0.2803
8925	HERC1	-0.2803
55432	YOD1	-0.2803
84706	GPT2	-0.2805
51665	ASB1	-0.2806
3385	ICAM3	-0.2808
2824	GPM6B	-0.2809
3049	HBQ1	-0.2811
122769	LRR1	-0.2811
23580	CDC42EP4	-0.2811
30001	ERO1A	-0.2815
677	ZFP36L1	-0.2816
90102	PHLDB2	-0.2816

9213	XPR1	-0.2817
9858	PPP1R26	-0.2818
11122	PTPRT	-0.2818
84866	TMEM25	-0.2819
127002	ATXN7L2	-0.2819
79149	ZSCAN5A	-0.2819
9168	TMSB10	-0.2819
2002	ELK1	-0.2821
84695	LOXL3	-0.2823
57795	BRINP2	-0.2826
320	APBA1	-0.2827
3231	HOXD1	-0.2829
64137	ABCG4	-0.2831
10478	SLC25A17	-0.2833
85455	DISP2	-0.2835
1303	COL12A1	-0.2835
92482	BBIP1	-0.2838
54550	NECAB2	-0.2839
8828	NRP2	-0.2839
54587	MXRA8	-0.2839
56474	CTPS2	-0.2841
57465	TBC1D24	-0.2842
64081	PBLD	-0.2843
114787	GPRIN1	-0.2846
5276	SERPINI2	-0.2846
54492	NEURL1B	-0.2847
117157	SH2D1B	-0.2849
23635	SSBP2	-0.2851
60492	CCDC90B	-0.2854
2558	GABRA5	-0.2858
3321	IGSF3	-0.2861
6574	SLC20A1	-0.2867
167410	LIX1	-0.2870
4781	NFIB	-0.2871
23333	DPY19L1	-0.2873
338657	CCDC84	-0.2873
6563	SLC14A1	-0.2874
27285	TEKT2	-0.2874
170679	PSORS1C1	-0.2877
3760	KCNJ3	-0.2877
6612	SUMO3	-0.2881
78990	OTUB2	-0.2881
118427	OLFM3	-0.2882
1001	CDH3	-0.2887
4144	MAT2A	-0.2887
92126	DSEL	-0.2889
10069	RWDD2B	-0.2890
967	CD63	-0.2892
6397	SEC14L1	-0.2896
285382	C3orf70	-0.2897
79781	IQCA1	-0.2899
79144	PPDPF	-0.2900
79022	TMEM106C	-0.2900
10202	DHRS2	-0.2901
4141	MARS	-0.2901

653319	KIAA0895L	-0.2901
84222	TMEM191A	-0.2901
91156	IGFN1	-0.2902
23216	TBC1D1	-0.2903
6650	CAPN15	-0.2904
8557	TCAP	-0.2904
10534	SSSCA1	-0.2907
123920	CMTM3	-0.2908
4281	MID1	-0.2909
23151	GRAMD4	-0.2909
90835	CCDC189	-0.2915
9731	CEP104	-0.2917
51364	ZMYND10	-0.2918
285596	FAM153A	-0.2918
5360	PLTP	-0.2920
89846	FGD3	-0.2922
11130	ZWINT	-0.2923
2982	GUCY1A1	-0.2924
8447	DOC2B	-0.2924
9077	DIRAS3	-0.2924
284716	RIMKLA	-0.2925
8857	FCGBP	-0.2926
28232	SLCO3A1	-0.2930
8646	CHRD	-0.2931
219539	YPEL4	-0.2934
960	CD44	-0.2937
124056	NOXO1	-0.2941
6236	RRAD	-0.2943
79070	KDELC1	-0.2947
55220	KLHDC8A	-0.2949
1951	CELSR3	-0.2953
349136	WDR86	-0.2953
3687	ITGAX	-0.2956
64755	C16orf58	-0.2958
23461	ABCA5	-0.2959
11346	SYNPO	-0.2969
1428	CRYM	-0.2971
8777	MPDZ	-0.2972
79442	LRRC2	-0.2974
2532	ACKR1	-0.2978
10293	TRAIP	-0.2978
130814	PQLC3	-0.2980
6567	SLC16A2	-0.2981
84319	CMSS1	-0.2981
51304	ZDHHC3	-0.2988
25797	QPCT	-0.2990
1832	DSP	-0.2991
166752	FREM3	-0.2993
4494	MT1F	-0.2994
23209	MLC1	-0.2995
2768	GNA12	-0.3005
3773	KCNJ16	-0.3007
55803	ADAP2	-0.3009
4889	NPY5R	-0.3010
90423	ATP6V1E2	-0.3010

2006	ELN	-0.3012
53342	IL17D	-0.3015
129450	TYW5	-0.3015
23143	LRCH1	-0.3015
57608	JCAD	-0.3016
55466	DNAJA4	-0.3018
258010	SVIP	-0.3020
3398	ID2	-0.3022
124540	MSI2	-0.3022
2012	EMP1	-0.3023
83982	IFI27L2	-0.3025
7423	VEGFB	-0.3026
10993	SDS	-0.3027
5826	ABCD4	-0.3030
6553	SLC9A5	-0.3032
70	ACTC1	-0.3039
339559	ZFP69	-0.3040
29115	SAP30BP	-0.3040
9721	GPRIN2	-0.3041
7466	WFS1	-0.3041
51143	DYNC1LI1	-0.3042
23429	RYBP	-0.3049
25809	TTLL1	-0.3051
6118	RPA2	-0.3058
79447	PAGR1	-0.3059
5792	PTPRF	-0.3059
283078	MKX	-0.3061
55062	WIPI1	-0.3061
151790	WDR49	-0.3067
2239	GPC4	-0.3068
25925	ZNF521	-0.3075
5019	OXCT1	-0.3075
5796	PTPRK	-0.3082
9938	ARHGAP25	-0.3084
389	RHOC	-0.3085
84808	PERM1	-0.3086
345079	SOWAHB	-0.3087
8973	CHRNA6	-0.3089
341	APOC1	-0.3090
81606	LBH	-0.3098
57194	ATP10A	-0.3102
2842	GPR19	-0.3103
23517	MTREX	-0.3103
58504	ARHGAP22	-0.3105
115426	UHRF2	-0.3106
116028	RMI2	-0.3107
9289	ADGRG1	-0.3109
7772	ZNF229	-0.3110
79874	RABEP2	-0.3113
4008	LMO7	-0.3115
54360	CYTL1	-0.3115
2894	GRID1	-0.3117
11231	SEC63	-0.3119
8659	ALDH4A1	-0.3119
2690	GHR	-0.3123

283174	MIR4697HG	-0.3124
29958	DMGDH	-0.3129
2064	ERBB2	-0.3129
55222	LRRC20	-0.3134
3141	HLCS	-0.3135
81602	CDADC1	-0.3142
10656	KHDRBS3	-0.3144
57576	KIF17	-0.3145
222166	MTURN	-0.3148
596	BCL2	-0.3148
79026	AHNAK	-0.3151
5872	RAB13	-0.3152
654502	IQCJ	-0.3152
8459	TPST2	-0.3155
158798	AKAP14	-0.3158
219902	TMEM136	-0.3160
2172	FABP6	-0.3162
5198	PFAS	-0.3164
27237	ARHGEF16	-0.3166
4502	MT2A	-0.3167
27154	BRPF3	-0.3171
942	CD86	-0.3172
187	APLNR	-0.3172
112616	CMTM7	-0.3176
140710	SOGA1	-0.3177
5067	CNTN3	-0.3178
91947	ARRDC4	-0.3179
8927	BSN	-0.3179
4499	MT1M	-0.3180
157574	FBXO16	-0.3182
366	AQP9	-0.3182
3014	H2AFX	-0.3183
10208	USPL1	-0.3187
6533	SLC6A6	-0.3190
51527	GSKIP	-0.3192
79443	FYCO1	-0.3198
93323	HAUS8	-0.3200
9609	RAB36	-0.3200
2560	GABRB1	-0.3201
83786	FRMD8	-0.3203
8673	VAMP8	-0.3204
8935	SKAP2	-0.3205
80024	SLC8B1	-0.3205
2537	IFI6	-0.3207
55714	TENM3	-0.3210
57670	KIAA1549	-0.3213
55659	ZNF416	-0.3215
4604	MYBPC1	-0.3215
23086	EXPH5	-0.3220
93587	TRMT10A	-0.3220
55015	PRPF39	-0.3224
51642	MRPL48	-0.3224
134121	C5orf49	-0.3224
6347	CCL2	-0.3226
55596	ZCCHC8	-0.3228

260434	PYDC1	-0.3229
57122	NUP107	-0.3239
4794	NFKBIE	-0.3240
4771	NF2	-0.3241
8642	DCHS1	-0.3245
7076	TIMP1	-0.3247
51168	MYO15A	-0.3248
90050	FAM181A	-0.3248
11067	DEPP1	-0.3250
55190	NUDT11	-0.3252
54954	FAM120C	-0.3253
79760	GEMIN7	-0.3255
10178	TENM1	-0.3256
3978	LIG1	-0.3256
6786	STIM1	-0.3257
57533	TBC1D14	-0.3259
92399	MRRF	-0.3267
4062	LY6H	-0.3268
9508	ADAMTS3	-0.3268
56829	ZC3HAV1	-0.3269
6277	S100A6	-0.3269
51706	CYB5R1	-0.3271
1827	RCAN1	-0.3274
151531	UPP2	-0.3274
54627	MAP10	-0.3275
57101	ANO2	-0.3278
257019	FRMD3	-0.3282
55090	MED9	-0.3282
23245	ASTN2	-0.3283
83874	TBC1D10A	-0.3283
945	CD33	-0.3284
26872	STEAP1	-0.3292
5582	PRKCG	-0.3297
3074	HEXB	-0.3297
7071	KLF10	-0.3297
149111	CNIH3	-0.3298
23608	MKRN1	-0.3300
133686	NADK2	-0.3301
22808	MRAS	-0.3301
200634	KRTCAP3	-0.3302
57084	SLC17A6	-0.3304
23474	ETHE1	-0.3306
55897	MESP1	-0.3309
5988	RFPL1	-0.3312
6017	RLBP1	-0.3315
199223	TTC21A	-0.3318
60561	RINT1	-0.3320
9734	HDAC9	-0.3321
11180	WDR6	-0.3322
641	BLM	-0.3322
135138	PACRG	-0.3326
7060	THBS4	-0.3332
100133941	CD24	-0.3333
4609	MYC	-0.3333
388886	LRRC75B	-0.3333

200942	KLHDC8B	-0.3338
7769	ZNF226	-0.3343
51668	HSPB11	-0.3343
57406	ABHD6	-0.3344
163175	LGI4	-0.3345
166647	ADGRA3	-0.3350
79958	DENND1C	-0.3350
254887	ZDHHC23	-0.3351
161931	ADAD2	-0.3355
51115	RMDN1	-0.3356
6622	SNCA	-0.3357
4923	NTSR1	-0.3358
146845	CFAP52	-0.3359
80221	ACSF2	-0.3359
7005	TEAD3	-0.3364
26471	NUPR1	-0.3364
93953	GCNA	-0.3365
219348	PLAC9	-0.3368
3363	HTR7	-0.3369
84305	PYM1	-0.3370
6920	TCEA3	-0.3374
3316	HSPB2	-0.3375
10144	FAM13A	-0.3377
10038	PARP2	-0.3379
23406	COTL1	-0.3380
114786	XKR4	-0.3380
4478	MSN	-0.3380
4968	OGG1	-0.3380
11189	CELF3	-0.3384
57110	HRASLS	-0.3384
63876	PKNOX2	-0.3387
140885	SIRPA	-0.3387
2512	FTL	-0.3389
150	ADRA2A	-0.3389
5446	PON3	-0.3392
27077	B9D1	-0.3395
4602	MYB	-0.3397
257236	CCDC96	-0.3398
26074	CFAP61	-0.3398
8706	B3GALNT1	-0.3399
23061	TBC1D9B	-0.3401
7056	THBD	-0.3401
441631	TSPAN11	-0.3401
23484	LEPROTL1	-0.3403
23428	SLC7A8	-0.3410
23759	PPIL2	-0.3412
158471	PRUNE2	-0.3414
1291	COL6A1	-0.3418
8671	SLC4A4	-0.3418
786	CACNG1	-0.3419
23518	R3HDM1	-0.3421
55884	WSB2	-0.3437
4705	NDUFA10	-0.3438
63974	NEUROD6	-0.3440
10090	UST	-0.3440

23671	TMEFF2	-0.3440
497190	CLEC18B	-0.3443
84439	HHIPL1	-0.3445
129303	TMEM150A	-0.3445
197335	WDR90	-0.3446
2905	GRIN2C	-0.3452
55002	TMCO3	-0.3452
7188	TRAF5	-0.3456
6457	SH3GL3	-0.3461
58494	JAM2	-0.3462
5954	RCN1	-0.3465
6472	SHMT2	-0.3472
147968	CAPN12	-0.3477
55103	RALGPS2	-0.3477
90850	ZNF598	-0.3477
7409	VAV1	-0.3478
9758	FRMPD4	-0.3482
6307	MSMO1	-0.3483
9874	TLK1	-0.3484
84623	KIRREL3	-0.3485
5326	PLAGL2	-0.3487
2027	ENO3	-0.3493
2990	GUSB	-0.3496
5324	PLAG1	-0.3498
286075	ZNF707	-0.3499
3691	ITGB4	-0.3499
9693	RAPGEF2	-0.3502
8310	ACOX3	-0.3505
4014	LOR	-0.3506
10235	RASGRP2	-0.3508
57099	AVEN	-0.3512
8406	SRPX	-0.3513
54972	TMEM132A	-0.3513
93986	FOXP2	-0.3514
1381	CRABP1	-0.3515
160622	GRASP	-0.3527
10785	WDR4	-0.3527
30818	KCNIP3	-0.3528
3553	IL1B	-0.3528
168391	GALNTL5	-0.3531
2595	GANC	-0.3533
10739	RFPL2	-0.3538
284427	SLC25A41	-0.3541
9616	RNF7	-0.3541
64219	PJA1	-0.3548
57153	SLC44A2	-0.3549
11279	KLF8	-0.3549
55364	IMPACT	-0.3550
401262	CRIP3	-0.3552
118491	CFAP70	-0.3552
4855	NOTCH4	-0.3554
389812	LCN15	-0.3559
146754	DNAH2	-0.3559
60468	BACH2	-0.3564
57139	RGL3	-0.3572

1952	CELSR2	-0.3572
54566	EPB41L4B	-0.3572
148022	TICAM1	-0.3573
10690	FUT9	-0.3578
55615	PRR5	-0.3578
5156	PDGFRA	-0.3583
207063	DHRSX	-0.3584
64150	DIO3OS	-0.3585
885	CCK	-0.3587
221496	LEMD2	-0.3588
57546	PDP2	-0.3589
51133	KCTD3	-0.3592
2307	FOXS1	-0.3593
642273	FAM110C	-0.3596
161357	MDGA2	-0.3598
127933	UHMK1	-0.3601
2800	GOLGA1	-0.3601
79660	PPP1R3B	-0.3605
121260	SLC15A4	-0.3611
8309	ACOX2	-0.3612
1948	EFNB2	-0.3613
414919	C8orf82	-0.3613
157697	ERICH1	-0.3622
11255	HRH3	-0.3629
7352	UCP3	-0.3630
124045	SPATA33	-0.3630
10219	KLRG1	-0.3632
64919	BCL11B	-0.3633
6249	CLIP1	-0.3634
28985	MCTS1	-0.3635
387775	SLC22A10	-0.3639
23493	HEY2	-0.3641
10200	MPHOSPH6	-0.3642
54039	PCBP3	-0.3643
1069	CETN2	-0.3644
374875	HSD11B1L	-0.3644
5721	PSME2	-0.3647
5092	PCBD1	-0.3650
22919	MAPRE1	-0.3651
1051	CEBPB	-0.3651
8437	RASAL1	-0.3652
339448	C1orf174	-0.3655
5002	SLC22A18	-0.3659
29799	YPEL1	-0.3664
22986	SORCS3	-0.3665
7534	YWHAZ	-0.3666
93109	TMEM44	-0.3667
54847	SIDT1	-0.3667
8574	AKR7A2	-0.3669
22885	ABLIM3	-0.3670
990	CDC6	-0.3674
84769	MPV17L2	-0.3676
151354	FAM84A	-0.3687
84456	L3MBTL3	-0.3695
10446	LRRN2	-0.3698

6793	STK10	-0.3704
4751	NEK2	-0.3705
23403	FBXO46	-0.3706
83989	FAM172A	-0.3710
57172	CAMK1G	-0.3711
146	ADRA1D	-0.3712
10057	ABCC5	-0.3718
164832	LONRF2	-0.3723
65078	RTN4R	-0.3726
4857	NOVA1	-0.3726
57419	SLC24A3	-0.3727
5971	RELB	-0.3727
129607	CMPK2	-0.3729
54677	CROT	-0.3729
10776	ARPP19	-0.3731
80311	KLHL15	-0.3733
79651	RHBDF2	-0.3736
254778	C8orf46	-0.3739
55122	AKIRIN2	-0.3741
80270	HSD3B7	-0.3742
430	ASCL2	-0.3745
3156	HMGCR	-0.3747
10613	ERLIN1	-0.3753
23564	DDAH2	-0.3754
51611	DPH5	-0.3759
1307	COL16A1	-0.3760
55013	MCUB	-0.3760
1545	CYP1B1	-0.3761
2199	FBLN2	-0.3761
728392	LOC728392	-0.3766
93622	LOC93622	-0.3769
10105	PPIF	-0.3769
64766	S100PBP	-0.3770
5294	PIK3CG	-0.3772
11211	FZD10	-0.3772
10493	VAT1	-0.3773
115399	LRRC56	-0.3774
2040	STOM	-0.3774
127254	ERICH3	-0.3777
27295	PDLIM3	-0.3777
23133	PHF8	-0.3780
57699	CPNE5	-0.3781
6588	SLN	-0.3786
6764	ST5	-0.3788
57575	PCDH10	-0.3792
307	ANXA4	-0.3796
81621	KAZALD1	-0.3798
8742	TNFSF12	-0.3800
79890	RIN3	-0.3803
284217	LAMA1	-0.3806
9340	GLP2R	-0.3807
1856	DVL2	-0.3809
1718	DHCR24	-0.3811
339524	LINC01140	-0.3813
9244	CRLF1	-0.3813

8862	APLN	-0.3815
9882	TBC1D4	-0.3828
10590	SCGN	-0.3830
4625	MYH7	-0.3837
113	ADCY7	-0.3839
56623	INPP5E	-0.3841
11031	RAB31	-0.3842
9051	PSTPIP1	-0.3842
84791	LINC00467	-0.3848
7993	UBXN8	-0.3854
3985	LIMK2	-0.3854
27258	LSM3	-0.3855
54058	C21orf58	-0.3855
79961	DENND2D	-0.3857
58157	NGB	-0.3857
50486	G0S2	-0.3859
143282	FGFBP3	-0.3860
50509	COL5A3	-0.3866
728568	C12orf73	-0.3869
6251	RSU1	-0.3869
57801	HES4	-0.3870
2191	FAP	-0.3872
6883	TAF12	-0.3874
124925	SEZ6	-0.3876
124454	EARS2	-0.3876
4128	MAOA	-0.3878
147	ADRA1B	-0.3880
83871	RAB34	-0.3881
84696	ABHD1	-0.3882
2139	EYA2	-0.3887
155382	VPS37D	-0.3890
9474	ATG5	-0.3894
345557	PLCXD3	-0.3894
1290	COL5A2	-0.3895
678	ZFP36L2	-0.3896
57232	ZNF630	-0.3897
23576	DDAH1	-0.3898
81706	PPP1R14C	-0.3906
129642	MBOAT2	-0.3906
221421	RSPH9	-0.3913
10242	KCNMB2	-0.3914
590	BCHE	-0.3918
10228	STX6	-0.3920
255743	NPNT	-0.3923
10952	SEC61B	-0.3924
2890	GRIA1	-0.3928
7200	TRH	-0.3936
400793	C1orf226	-0.3936
285780	LY86-AS1	-0.3940
318	NUDT2	-0.3945
80003	PCNX2	-0.3945
10850	CCL27	-0.3954
8644	AKR1C3	-0.3954
100129460	DPY19L1P1	-0.3958
25759	SHC2	-0.3959

4988	OPRM1	-0.3962
55092	TMEM51	-0.3966
8527	DGKD	-0.3966
2184	FAH	-0.3968
57348	TTYH1	-0.3974
5272	SERPINB9	-0.3977
2908	NR3C1	-0.3980
8874	ARHGEF7	-0.3982
441108	C5orf56	-0.3985
6016	RIT1	-0.3987
9940	DLEC1	-0.3992
89795	NAV3	-0.3992
5650	KLK7	-0.3993
201627	DENND6A	-0.3993
81888	HYI	-0.3993
25871	NEPRO	-0.3993
6543	SLC8A2	-0.3994
2949	GSTM5	-0.3997
8787	RGS9	-0.3998
40	ASIC2	-0.4000
126374	WTIP	-0.4007
57216	VANGL2	-0.4009
23062	GGA2	-0.4009
5409	PNMT	-0.4010
55930	MYO5C	-0.4017
10154	PLXNC1	-0.4017
55170	PRMT6	-0.4019
7163	TPD52	-0.4022
8204	NRIP1	-0.4024
116496	FAM129A	-0.4027
136051	ZNF786	-0.4028
51725	FBXO40	-0.4028
2161	F12	-0.4028
84691	FAM71F1	-0.4030
5164	PDK2	-0.4034
26528	DAZAP1	-0.4035
727936	GXYLT2	-0.4035
5106	PCK2	-0.4036
10169	SERF2	-0.4039
57178	ZMIZ1	-0.4040
925	CD8A	-0.4043
550643	NBDY	-0.4048
124152	IQCK	-0.4048
284184	NDUFAF8	-0.4049
116832	RPL39L	-0.4052
4779	NFE2L1	-0.4052
400410	ST20	-0.4058
57683	ZDBF2	-0.4059
83660	TLN2	-0.4061
90025	UBE3D	-0.4064
59348	ZNF350	-0.4064
23022	PALLD	-0.4070
57179	KIAA1191	-0.4070
254863	TMEM256	-0.4070
79589	RNF128	-0.4074

5138	PDE2A	-0.4075
163933	FAM43B	-0.4078
4685	NCAM2	-0.4083
343450	KCNT2	-0.4087
10943	MSL3	-0.4088
9211	LGI1	-0.4089
2735	GLI1	-0.4101
23129	PLXND1	-0.4102
3956	LGALS1	-0.4102
375323	LHFPL4	-0.4105
83442	SH3BGRL3	-0.4107
51222	ZNF219	-0.4108
358	AQP1	-0.4109
283	ANG	-0.4112
112476	PRRT2	-0.4113
23170	TTLL12	-0.4115
130271	PLEKHH2	-0.4115
57495	NWD2	-0.4118
282969	FUOM	-0.4125
65975	STK33	-0.4126
6489	ST8SIA1	-0.4126
7533	YWHAH	-0.4136
4256	MGP	-0.4140
7092	TLL1	-0.4140
149473	CCDC24	-0.4142
27341	RRP7A	-0.4142
7301	TYRO3	-0.4142
4595	MUTYH	-0.4144
6281	S100A10	-0.4146
114801	TMEM200A	-0.4148
57496	MKL2	-0.4160
4745	NELL1	-0.4165
94120	SYTL3	-0.4166
9722	NOS1AP	-0.4173
116372	LYPD1	-0.4173
6660	SOX5	-0.4173
9939	RBM8A	-0.4173
9903	KLHL21	-0.4176
387758	FIBIN	-0.4177
23743	BHMT2	-0.4177
9536	PTGES	-0.4177
64881	PCDH20	-0.4179
8273	SLC10A3	-0.4181
57620	STIM2	-0.4183
1184	CLCN5	-0.4186
122402	TDRD9	-0.4193
9201	DCLK1	-0.4196
54756	IL17RD	-0.4200
4046	LSP1	-0.4201
55890	GPRC5C	-0.4204
9951	HS3ST4	-0.4205
1305	COL13A1	-0.4209
51760	SYT17	-0.4210
55686	MREG	-0.4212
7480	WNT10B	-0.4214

27151	CPAMD8	-0.4218
9232	PTTG1	-0.4218
93487	MAPK1IP1L	-0.4225
1314	COPA	-0.4227
9681	DEPDC5	-0.4230
8997	KALRN	-0.4241
1735	DIO3	-0.4241
401647	GOLGA7B	-0.4250
58489	ABHD17C	-0.4250
5774	PTPN3	-0.4252
1809	DPYSL3	-0.4253
9317	PTER	-0.4253
284415	VSTM1	-0.4255
79822	ARHGAP28	-0.4260
55120	FANCL	-0.4263
140458	ASB5	-0.4267
81552	VOPP1	-0.4273
762	CA4	-0.4276
128414	NKAIN4	-0.4279
84303	CHCHD6	-0.4280
116986	AGAP2	-0.4280
7342	UBP1	-0.4283
84187	TMEM164	-0.4283
389792	IER5L	-0.4291
641654	HEPN1	-0.4294
57705	WDFY4	-0.4296
114987	WDR31	-0.4300
9766	SUSD6	-0.4301
90523	MLIP	-0.4305
54431	DNAJC10	-0.4310
51232	CRIM1	-0.4313
85364	ZCCHC3	-0.4315
2115	ETV1	-0.4320
7038	TG	-0.4321
3595	IL12RB2	-0.4322
51429	SNX9	-0.4326
55591	VEZT	-0.4333
27132	CPNE7	-0.4337
266727	MDGA1	-0.4337
66000	TMEM108	-0.4338
126272	EID2B	-0.4338
57626	KLHL1	-0.4339
116135	LRRC3B	-0.4339
26472	PPP1R14B	-0.4340
219736	STOX1	-0.4340
51362	CDC40	-0.4340
25818	KLK5	-0.4347
10577	NPC2	-0.4347
39	ACAT2	-0.4350
49855	SCAPER	-0.4358
284207	METRNL	-0.4359
923	CD6	-0.4361
126969	SLC44A3	-0.4362
11017	SNRNP27	-0.4368
5836	PYGL	-0.4370

50617	ATP6V0A4	-0.4371
1032	CDKN2D	-0.4373
130497	OSR1	-0.4377
10865	ARID5A	-0.4379
90529	STPG1	-0.4379
4363	ABCC1	-0.4381
7138	TNNT1	-0.4381
55207	ARL8B	-0.4383
11178	LZTS1	-0.4384
83987	CCDC8	-0.4384
85416	ZIC5	-0.4386
415117	STX19	-0.4398
85019	TMEM241	-0.4399
2281	FKBP1B	-0.4399
132204	SYNPR	-0.4401
4482	MSRA	-0.4401
1036	CDO1	-0.4411
2742	GLRA2	-0.4411
3587	IL10RA	-0.4417
79183	TTPAL	-0.4418
503538	A1BG-AS1	-0.4428
54749	EPDR1	-0.4429
5212	VIT	-0.4433
9873	FCHSD2	-0.4434
11275	KLHL2	-0.4439
23119	HIC2	-0.4440
55612	FERMT1	-0.4443
9540	TP53I3	-0.4444
113130	CDCA5	-0.4453
113452	TMEM54	-0.4456
65982	ZSCAN18	-0.4456
84215	ZNF541	-0.4461
128611	ZNF831	-0.4462
3556	IL1RAP	-0.4466
51155	JPT1	-0.4467
55282	LRRC36	-0.4467
6328	SCN3A	-0.4469
9823	ARMCX2	-0.4471
1063	CENPF	-0.4471
30061	SLC40A1	-0.4475
55140	ELP3	-0.4476
25996	REXO2	-0.4477
55856	ACOT13	-0.4478
5733	PTGER3	-0.4483
6608	SMO	-0.4490
11159	RABL2A	-0.4490
246213	SLC17A8	-0.4491
9110	MTMR4	-0.4495
79813	EHMT1	-0.4500
55937	APOM	-0.4501
80324	PUS1	-0.4503
92335	STRADA	-0.4506
8460	TPST1	-0.4507
124359	CDYL2	-0.4518
151195	CCNYL1	-0.4521

1978	EIF4EBP1	-0.4530
58475	MS4A7	-0.4531
23087	TRIM35	-0.4531
201229	LYRM9	-0.4537
9535	GMFG	-0.4537
347744	C6orf52	-0.4538
51191	HERC5	-0.4540
10458	BAIAP2	-0.4541
149175	MANEAL	-0.4541
5831	PYCR1	-0.4545
340348	TSPAN33	-0.4549
57631	LRCH2	-0.4549
1009	CDH11	-0.4552
388730	TMEM81	-0.4552
84217	ZMYND12	-0.4557
9993	DGCR2	-0.4561
89958	SAPCD2	-0.4567
11163	NUDT4	-0.4568
57718	PPP4R4	-0.4570
115207	KCTD12	-0.4570
23780	APOL2	-0.4575
23217	ZFR2	-0.4576
503693	LOH12CR2	-0.4580
56934	CA10	-0.4581
54850	FBXL12	-0.4582
222865	TMEM130	-0.4586
1796	DOK1	-0.4587
3643	INSR	-0.4589
81562	LMAN2L	-0.4590
22849	CPEB3	-0.4603
4983	OPHN1	-0.4610
144406	WDR66	-0.4611
56127	PCDHB9	-0.4613
6659	SOX4	-0.4616
152	ADRA2C	-0.4619
9467	SH3BP5	-0.4620
11021	RAB35	-0.4620
79870	BAALC	-0.4621
594855	CPLX3	-0.4625
7867	MAPKAPK3	-0.4651
138428	PTRH1	-0.4660
4772	NFATC1	-0.4664
6094	ROM1	-0.4664
93210	PGAP3	-0.4665
11040	PIM2	-0.4669
147650	SPACA6	-0.4676
7701	ZNF142	-0.4680
55794	DDX28	-0.4681
727	C5	-0.4682
83468	GLT8D2	-0.4684
79814	AGMAT	-0.4685
844	CASQ1	-0.4686
64770	CCDC14	-0.4688
669	BPGM	-0.4688
282973	JAKMIP3	-0.4697

28986	MAGEH1	-0.4703
164781	DAW1	-0.4706
652968	CASTOR1	-0.4716
65263	PYCR3	-0.4716
1176	AP3S1	-0.4717
66005	CHID1	-0.4718
85443	DCLK3	-0.4728
400073	C12orf76	-0.4729
256281	NUDT14	-0.4743
84168	ANTXR1	-0.4744
2650	GCNT1	-0.4748
54836	BSPRY	-0.4753
389075	RESP18	-0.4758
57664	PLEKHA4	-0.4759
56884	FSTL5	-0.4759
84255	SLC37A3	-0.4763
4986	OPRK1	-0.4769
139221	MUM1L1	-0.4772
80020	FOXRED2	-0.4774
51134	CEP83	-0.4776
8988	HSPB3	-0.4778
8631	SKAP1	-0.4784
3881	KRT31	-0.4787
6751	SSTR1	-0.4789
145226	RDH12	-0.4793
373861	HILS1	-0.4794
347733	TUBB2B	-0.4795
386618	KCTD4	-0.4804
196051	PLPP4	-0.4806
7475	WNT6	-0.4806
100093630	SNHG8	-0.4807
7137	TNNI3	-0.4807
92558	BICDL1	-0.4826
128434	VSTM2L	-0.4828
80323	CCDC68	-0.4832
79650	USB1	-0.4832
9399	STOML1	-0.4834
190	NR0B1	-0.4837
55086	CXorf57	-0.4837
7737	RNF113A	-0.4840
4886	NPY1R	-0.4841
6422	SFRP1	-0.4846
79042	TSEN34	-0.4855
23643	LY96	-0.4860
85446	ZFHX2	-0.4861
84063	KIRREL2	-0.4864
54809	SAMD9	-0.4865
54536	EXOC6	-0.4868
23037	PDZD2	-0.4877
6713	SQLE	-0.4883
9532	BAG2	-0.4883
4878	NPPA	-0.4901
26002	MOXD1	-0.4911
624	BDKRB2	-0.4913
1675	CFD	-0.4914

388585	HES5	-0.4916
83636	C19orf12	-0.4919
79772	MCTP1	-0.4932
285489	DOK7	-0.4933
4097	MAFG	-0.4935
10923	SUB1	-0.4941
11066	SNRNP35	-0.4944
9312	KCNB2	-0.4951
441024	MTHFD2L	-0.4954
197257	LDHD	-0.4954
286336	FAM78A	-0.4958
9580	SOX13	-0.4958
23401	FRAT2	-0.4961
84536	LINC01547	-0.4964
9332	CD163	-0.4968
3784	KCNQ1	-0.4969
192668	CYS1	-0.4972
59285	CACNG6	-0.4974
6444	SGCD	-0.4975
1174	AP1S1	-0.4979
6335	SCN9A	-0.4993
4239	MFAP4	-0.4995
56954	NIT2	-0.4998
79000	AUNIP	-0.4999
127845	GOLT1A	-0.5002
146664	MGAT5B	-0.5009
1592	CYP26A1	-0.5012
64131	XYLT1	-0.5015
10842	PPP1R17	-0.5015
5819	NECTIN2	-0.5015
26127	FGFR1OP2	-0.5018
8938	BAIAP3	-0.5021
389538	CCZ1P-OR7E3	-0.5023
8193	DPF1	-0.5027
83879	CDCA7	-0.5027
1717	DHCR7	-0.5030
253190	SERHL2	-0.5033
8445	DYRK2	-0.5035
223117	SEMA3D	-0.5041
84790	TUBA1C	-0.5045
23314	SATB2	-0.5049
10346	TRIM22	-0.5049
196527	ANO6	-0.5050
139411	PTCHD1	-0.5061
644353	ZCCHC18	-0.5065
7262	PHLDA2	-0.5067
10160	FARP1	-0.5074
23180	RFTN1	-0.5074
23367	LARP1	-0.5084
53838	C11orf24	-0.5090
646627	LYPD8	-0.5091
388722	C1orf53	-0.5092
26508	HEYL	-0.5095
84935	MEDAG	-0.5098
79047	KCTD15	-0.5106

26037	SIPA1L1	-0.5114
80114	BICC1	-0.5116
85013	TMEM128	-0.5117
100128731	OST4	-0.5119
6503	SLA	-0.5127
79623	GALNT14	-0.5133
9215	LARGE1	-0.5141
389058	SP5	-0.5143
57596	BEGAIN	-0.5168
83930	STARD3NL	-0.5168
3764	KCNJ8	-0.5169
54549	SDK2	-0.5172
92196	DAPL1	-0.5182
5125	PCSK5	-0.5198
8764	TNFRSF14	-0.5198
10148	EBI3	-0.5203
132112	RTP1	-0.5209
79877	DCAKD	-0.5211
55715	DOK4	-0.5220
23630	KCNE5	-0.5222
64420	SUSD1	-0.5224
64792	IFT22	-0.5225
9794	MAML1	-0.5229
1767	DNAH5	-0.5229
2662	GDF10	-0.5236
126823	KLHDC9	-0.5246
59345	GNB4	-0.5262
5795	PTPRJ	-0.5263
55616	ASAP3	-0.5267
83643	CCDC3	-0.5271
285381	DPH3	-0.5272
117154	DACH2	-0.5274
8792	TNFRSF11A	-0.5274
56475	RPRM	-0.5274
129852	C2orf73	-0.5282
8321	FZD1	-0.5286
57088	PLSCR4	-0.5286
51655	RASD1	-0.5294
7306	TYRP1	-0.5295
3033	HADH	-0.5295
4837	NNMT	-0.5304
2764	GMFB	-0.5304
2911	GRM1	-0.5320
55916	NXT2	-0.5322
10894	LYVE1	-0.5333
122786	FRMD6	-0.5337
9394	HS6ST1	-0.5353
55228	PNMA8A	-0.5355
6545	SLC7A4	-0.5362
58472	SQOR	-0.5363
221491	SMIM29	-0.5364
53405	CLIC5	-0.5365
57326	PBXIP1	-0.5372
2737	GLI3	-0.5378
9140	ATG12	-0.5378

284129	SLC26A11	-0.5388
196383	RILPL2	-0.5393
9415	FADS2	-0.5395
23531	MMD	-0.5395
9615	GDA	-0.5413
285989	ZNF789	-0.5415
27345	KCNMB4	-0.5416
152189	CMTM8	-0.5416
5865	RAB3B	-0.5418
574029	DUSP5P1	-0.5418
4192	MDK	-0.5422
26059	ERC2	-0.5435
9111	NMI	-0.5438
926	CD8B	-0.5439
79148	MMP28	-0.5446
26086	GPSM1	-0.5450
51309	ARMCX1	-0.5453
8739	HRK	-0.5459
445577	C9orf129	-0.5463
58495	OVOL2	-0.5467
5341	PLEK	-0.5472
283143	LINC00900	-0.5477
399948	COLCA1	-0.5483
56731	SLC2A4RG	-0.5483
7802	DNALI1	-0.5503
64778	FNDC3B	-0.5504
27147	DENND2A	-0.5509
143279	HECTD2	-0.5514
57007	ACKR3	-0.5514
56034	PDGFC	-0.5515
2830	GPR6	-0.5521
155185	AMZ1	-0.5526
2882	GPX7	-0.5527
79746	ECHDC3	-0.5541
11257	TP53TG1	-0.5543
162073	ITPRIPL2	-0.5543
10417	SPON2	-0.5547
22929	SEPHS1	-0.5548
51015	ISOC1	-0.5548
5502	PPP1R1A	-0.5550
6640	SNTA1	-0.5570
343637	RSPO4	-0.5579
51094	ADIPOR1	-0.5580
644139	PIRT	-0.5585
65997	RASL11B	-0.5591
23094	SIPA1L3	-0.5592
85439	STON2	-0.5595
5292	PIM1	-0.5597
10186	LHFPL6	-0.5611
23007	PLCH1	-0.5611
6239	RREB1	-0.5616
7026	NR2F2	-0.5616
84870	RSPO3	-0.5616
85236	HIST1H2BK	-0.5616
1871	E2F3	-0.5619

90488	TMEM263	-0.5621
92597	MOB1B	-0.5623
4885	NPTX2	-0.5626
317762	CCDC85C	-0.5635
57630	SH3RF1	-0.5636
51123	ZNF706	-0.5636
26519	TIMM10	-0.5638
5569	PKIA	-0.5641
5136	PDE1A	-0.5652
9653	HS2ST1	-0.5654
3358	HTR2C	-0.5658
9308	CD83	-0.5663
84332	DYDC2	-0.5666
782	CACNB1	-0.5673
57188	ADAMTSL3	-0.5676
2030	SLC29A1	-0.5694
57484	RNF150	-0.5706
58525	WIZ	-0.5709
3738	KCNA3	-0.5711
147699	PPM1N	-0.5712
150147	UMODL1-AS1	-0.5713
27145	FILIP1	-0.5737
84552	PARD6G	-0.5745
94134	ARHGAP12	-0.5750
139285	AMER1	-0.5771
63933	MCUR1	-0.5773
56652	TWNK	-0.5775
154215	NKAIN2	-0.5784
7227	TRPS1	-0.5785
55742	PARVA	-0.5785
84628	NTNG2	-0.5789
5173	PDYN	-0.5791
3598	IL13RA2	-0.5796
8444	DYRK3	-0.5796
147372	CCBE1	-0.5809
9750	RIPOR2	-0.5824
8573	CASK	-0.5830
1043	CD52	-0.5830
113277	TMEM106A	-0.5836
219285	SAMD9L	-0.5844
644189	LOC644189	-0.5861
79751	SLC25A22	-0.5868
441027	TMEM150C	-0.5871
114884	OSBPL10	-0.5873
2918	GRM8	-0.5885
57504	MTA3	-0.5889
51659	GINS2	-0.5890
862	RUNX1T1	-0.5892
51087	YBX2	-0.5896
80339	PNPLA3	-0.5899
5880	RAC2	-0.5907
8322	FZD4	-0.5914
3741	KCNA5	-0.5919
8111	GPR68	-0.5934
79656	BEND5	-0.5946

6441	SFTPD	-0.5959
51246	SHISA5	-0.5959
25924	MYRIP	-0.5972
4948	OCA2	-0.5979
158293	FAM120AOS	-0.5980
64388	GREM2	-0.5999
2280	FKBP1A	-0.6011
362	AQP5	-0.6016
1163	CKS1B	-0.6019
25852	ARMC8	-0.6024
51538	ZCCHC17	-0.6027
63982	ANO3	-0.6033
81578	COL21A1	-0.6036
84314	TMEM107	-0.6051
10505	SEMA4F	-0.6057
1464	CSPG4	-0.6058
57687	VAT1L	-0.6063
4016	LOXL1	-0.6083
10021	HCN4	-0.6099
5475	PPEF1	-0.6125
80309	SPHKAP	-0.6144
399726	CASC10	-0.6147
56950	SMYD2	-0.6165
83759	RBM4B	-0.6184
51063	CALHM2	-0.6186
83857	TMTC1	-0.6188
9636	ISG15	-0.6196
2963	GTF2F2	-0.6216
64841	GNPNAT1	-0.6220
55634	KRBOX4	-0.6224
4086	SMAD1	-0.6236
6770	STAR	-0.6239
57146	TMEM159	-0.6246
6575	SLC20A2	-0.6248
126792	B3GALT6	-0.6258
29798	C2orf27A	-0.6263
2564	GABRE	-0.6279
219527	LRRC55	-0.6283
116443	GRIN3A	-0.6294
23360	FNBP4	-0.6316
1875	E2F5	-0.6320
100170841	EPOP	-0.6381
284611	FAM102B	-0.6385
441168	CALHM6	-0.6391
23462	HEY1	-0.6396
10046	MAMLD1	-0.6398
2331	FMOD	-0.6411
4856	NOV	-0.6414
3268	AGFG2	-0.6426
55530	SVOP	-0.6433
79645	EFCAB1	-0.6470
605	BCL7A	-0.6473
5912	RAP2B	-0.6490
286133	SCARA5	-0.6499
401720	FIGNL2	-0.6514

6778	STAT6	-0.6519
51363	CHST15	-0.6521
84830	ADTRP	-0.6531
23503	ZFYVE26	-0.6542
84289	ING5	-0.6551
645191	LINGO3	-0.6556
347902	AMIGO2	-0.6557
55521	TRIM36	-0.6560
81619	TSPAN14	-0.6568
7127	TNFAIP2	-0.6570
27112	FAM155B	-0.6580
54874	FNBP1L	-0.6584
55734	ZFP64	-0.6598
80714	PBX4	-0.6618
285704	RGMB	-0.6634
170685	NUDT10	-0.6639
100288911	LOC100288911	-0.6658
85409	NKD2	-0.6670
284485	RIIAD1	-0.6679
22881	ANKRD6	-0.6699
7010	TEK	-0.6702
330	BIRC3	-0.6719
126789	PUSL1	-0.6731
56477	CCL28	-0.6740
6804	STX1A	-0.6747
54733	SLC35F2	-0.6766
6664	SOX11	-0.6787
91663	MYADM	-0.6829
56603	CYP26B1	-0.6857
378884	NHLRC1	-0.6892
25907	TMEM158	-0.6902
1116	CHI3L1	-0.6910
196500	PIANP	-0.6916
4718	NDUFC2	-0.6947
23251	KIAA1024	-0.6949
4082	MARCKS	-0.6961
57722	IGDCC4	-0.6974
84265	POLR3GL	-0.6986
5361	PLXNA1	-0.7006
10457	GPNMB	-0.7009
54221	SNTG2	-0.7042
127003	C1orf194	-0.7081
339390	CLEC4G	-0.7095
220202	ATOH7	-0.7140
131616	TMEM42	-0.7158
79132	DHX58	-0.7160
631	BFSP1	-0.7172
80115	BAIAP2L2	-0.7173
1137	CHRNA4	-0.7220
285601	GPR150	-0.7223
4004	LMO1	-0.7267
9033	PKD2L1	-0.7276
81558	FAM117A	-0.7325
56171	DNAH7	-0.7430
1390	CREM	-0.7470

80818	ZNF436	-0.7499
3957	LGALS2	-0.7510
55225	RAVER2	-0.7550
493	ATP2B4	-0.7565
10965	ACOT2	-0.7591
51031	GLOD4	-0.7653
4129	MAOB	-0.7738
114990	VASN	-0.7774
715	C1R	-0.7796
5655	KLK10	-0.7861
55959	SULF2	-0.8465
55022	PID1	-0.8484
716	C1S	-0.8689
27044	SND1	-0.8719

Abbreviations: PLS, partial least squares.
