# Supplementary material for: Functional hierarchy of the angular gyrus and its underlying genetic architecture
Source: Hum Brain Mapp. 2023 Feb 28;44(7):2815–28. doi: 10.1002/hbm.26247 (PMC10089092; doi:10.1002/hbm.26247)
Supplement: Supplementary file 3 — FILE S2. Enrichment results of the PLS+ genes [file HBM-44-2815-s001.pdf]

Enrichment results of the PLS+ genes

Category	ID	Name	*P* value	*q* value (FDR-BH correction)
GO: Molecular Function	GO:0008092	cytoskeletal protein binding	1.09E-05	1.16E-02
GO: Molecular Function	GO:0019905	syntaxin binding	4.09E-05	1.70E-02
GO: Molecular Function	GO:0044325	ion channel binding	7.46E-05	1.70E-02
GO: Molecular Function	GO:0030674	protein-macromolecule adaptor activity	8.97E-05	1.70E-02
GO: Molecular Function	GO:0044877	protein-containing complex binding	9.72E-05	1.70E-02
GO: Molecular Function	GO:0022835	transmitter-gated channel activity	1.11E-04	1.70E-02
GO: Molecular Function	GO:0022824	transmitter-gated ion channel activity	1.11E-04	1.70E-02
GO: Molecular Function	GO:0005516	calmodulin binding	1.86E-04	2.22E-02
GO: Molecular Function	GO:0016301	kinase activity	2.35E-04	2.22E-02
GO: Molecular Function	GO:1904315	transmitter-gated ion channel activity involved in regulation of postsynaptic membrane potential	2.53E-04	2.22E-02
GO: Molecular Function	GO:0000149	SNARE binding	2.62E-04	2.22E-02
GO: Molecular Function	GO:0046332	SMAD binding	2.67E-04	2.22E-02
GO: Molecular Function	GO:0016772	transferase activity, transferring phosphorus-containing groups	2.70E-04	2.22E-02
GO: Molecular Function	GO:0060090	molecular adaptor activity	3.14E-04	2.40E-02
GO: Molecular Function	GO:0099529	neurotransmitter receptor activity involved in regulation of postsynaptic membrane potential	3.60E-04	2.46E-02
GO: Molecular Function	GO:0022836	gated channel activity	4.30E-04	2.46E-02
GO: Molecular Function	GO:0016773	phosphotransferase activity, alcohol group as acceptor	4.32E-04	2.46E-02
GO: Molecular Function	GO:0017075	syntaxin-1 binding	4.35E-04	2.46E-02
GO: Molecular Function	GO:0019900	kinase binding	4.37E-04	2.46E-02
GO: Molecular Function	GO:0004672	protein kinase activity	5.02E-04	2.51E-02
GO: Molecular Function	GO:0005200	structural constituent of cytoskeleton	5.10E-04	2.51E-02
GO: Molecular Function	GO:0005230	extracellular ligand-gated ion channel activity	5.17E-04	2.51E-02
GO: Molecular Function	GO:0005237	inhibitory extracellular ligand-gated ion channel activity	6.43E-04	2.97E-02
GO: Molecular Function	GO:0019901	protein kinase binding	7.24E-04	2.97E-02
GO: Molecular Function	GO:0034596	phosphatidylinositol phosphate 4-phosphatase activity	7.35E-04	2.97E-02
GO: Molecular Function	GO:0015631	tubulin binding	7.42E-04	2.97E-02
GO: Molecular Function	GO:0008081	phosphoric diester hydrolase activity	7.51E-04	2.97E-02
GO: Molecular Function	GO:0003779	actin binding	8.67E-04	3.31E-02
GO: Molecular Function	GO:0015276	ligand-gated ion channel activity	9.68E-04	3.48E-02
GO: Molecular Function	GO:0004890	GABA-A receptor activity	1.01E-03	3.48E-02
GO: Molecular Function	GO:0098960	postsynaptic neurotransmitter receptor activity	1.01E-03	3.48E-02
GO: Molecular Function	GO:0017076	purine nucleotide binding	1.04E-03	3.48E-02
GO: Molecular Function	GO:0022834	ligand-gated channel activity	1.14E-03	3.70E-02
GO: Molecular Function	GO:0032555	purine ribonucleotide binding	1.20E-03	3.79E-02
GO: Molecular Function	GO:0099095	ligand-gated anion channel activity	1.50E-03	4.53E-02
GO: Molecular Function	GO:0032553	ribonucleotide binding	1.53E-03	4.53E-02
GO: Molecular Function	GO:0008427	calcium-dependent protein kinase inhibitor activity	1.75E-03	4.57E-02
GO: Molecular Function	GO:0003976	UDP-N-acetylglucosamine-lysosomal-enzyme N-acetylglucosaminephosphotransferase activity	1.75E-03	4.57E-02
GO: Molecular Function	GO:1905056	P-type calcium transporter activity involved in regulation of presynaptic cytosolic calcium ion concentration	1.75E-03	4.57E-02
GO: Molecular Function	GO:0009383	rRNA (cytosine-C5-)-methyltransferase activity	1.75E-03	4.57E-02
GO: Molecular Function	GO:0016917	GABA receptor activity	1.80E-03	4.57E-02
GO: Molecular Function	GO:0008093	cytoskeletal anchor activity	1.80E-03	4.57E-02
GO: Biological Process	GO:0099537	trans-synaptic signaling	7.44E-13	2.67E-09
GO: Biological Process	GO:0098916	anterograde trans-synaptic signaling	1.60E-12	2.67E-09
GO: Biological Process	GO:0007268	chemical synaptic transmission	1.60E-12	2.67E-09
GO: Biological Process	GO:0099536	synaptic signaling	1.85E-12	2.67E-09
GO: Biological Process	GO:0048666	neuron development	2.12E-10	2.44E-07
GO: Biological Process	GO:0030182	neuron differentiation	7.13E-10	6.86E-07
GO: Biological Process	GO:0031175	neuron projection development	8.49E-10	7.00E-07
GO: Biological Process	GO:0043269	regulation of ion transport	1.84E-09	1.28E-06
GO: Biological Process	GO:0048699	generation of neurons	2.00E-09	1.28E-06
GO: Biological Process	GO:0022008	neurogenesis	2.62E-09	1.44E-06
GO: Biological Process	GO:0023061	signal release	2.75E-09	1.44E-06
GO: Biological Process	GO:0050804	modulation of chemical synaptic transmission	3.89E-09	1.82E-06
GO: Biological Process	GO:0099177	regulation of trans-synaptic signaling	4.09E-09	1.82E-06
GO: Biological Process	GO:0007267	cell-cell signaling	5.56E-09	2.29E-06
GO: Biological Process	GO:0048812	neuron projection morphogenesis	1.29E-08	4.97E-06
GO: Biological Process	GO:0016079	synaptic vesicle exocytosis	1.41E-08	5.08E-06
GO: Biological Process	GO:0060627	regulation of vesicle-mediated transport	2.24E-08	7.35E-06
GO: Biological Process	GO:0120039	plasma membrane bounded cell projection morphogenesis	2.29E-08	7.35E-06
GO: Biological Process	GO:0030030	cell projection organization	2.44E-08	7.40E-06
GO: Biological Process	GO:0048858	cell projection morphogenesis	2.77E-08	7.98E-06
GO: Biological Process	GO:0120036	plasma membrane bounded cell projection organization	4.25E-08	1.17E-05
GO: Biological Process	GO:0032990	cell part morphogenesis	5.55E-08	1.46E-05
GO: Biological Process	GO:0042391	regulation of membrane potential	8.40E-08	2.11E-05
GO: Biological Process	GO:0048667	cell morphogenesis involved in neuron differentiation	9.92E-08	2.39E-05
GO: Biological Process	GO:0032989	cellular component morphogenesis	1.12E-07	2.58E-05
GO: Biological Process	GO:0006836	neurotransmitter transport	1.25E-07	2.77E-05
GO: Biological Process	GO:0099643	signal release from synapse	2.03E-07	4.19E-05
GO: Biological Process	GO:0007269	neurotransmitter secretion	2.03E-07	4.19E-05
GO: Biological Process	GO:0032880	regulation of protein localization	3.62E-07	7.21E-05
GO: Biological Process	GO:0140352	export from cell	3.90E-07	7.31E-05
GO: Biological Process	GO:0099003	vesicle-mediated transport in synapse	3.93E-07	7.31E-05
GO: Biological Process	GO:0000902	cell morphogenesis	4.25E-07	7.66E-05
GO: Biological Process	GO:0044057	regulation of system process	6.13E-07	1.07E-04
GO: Biological Process	GO:0000904	cell morphogenesis involved in differentiation	7.89E-07	1.34E-04
GO: Biological Process	GO:0046903	secretion	8.76E-07	1.44E-04
GO: Biological Process	GO:0001505	regulation of neurotransmitter levels	9.22E-07	1.48E-04
GO: Biological Process	GO:0043270	positive regulation of ion transport	9.81E-07	1.53E-04
GO: Biological Process	GO:0032940	secretion by cell	1.04E-06	1.58E-04
GO: Biological Process	GO:0060341	regulation of cellular localization	1.09E-06	1.62E-04
GO: Biological Process	GO:0051050	positive regulation of transport	1.14E-06	1.65E-04
GO: Biological Process	GO:0016082	synaptic vesicle priming	1.53E-06	2.16E-04
GO: Biological Process	GO:0061564	axon development	1.86E-06	2.55E-04
GO: Biological Process	GO:0010975	regulation of neuron projection development	2.63E-06	3.51E-04
GO: Biological Process	GO:0051240	positive regulation of multicellular organismal process	2.68E-06	3.51E-04
GO: Biological Process	GO:0048167	regulation of synaptic plasticity	2.93E-06	3.75E-04
GO: Biological Process	GO:1903827	regulation of cellular protein localization	3.09E-06	3.87E-04
GO: Biological Process	GO:0034330	cell junction organization	3.38E-06	4.15E-04
GO: Biological Process	GO:1990778	protein localization to cell periphery	3.46E-06	4.17E-04
GO: Biological Process	GO:0099504	synaptic vesicle cycle	3.97E-06	4.68E-04
GO: Biological Process	GO:0031630	regulation of synaptic vesicle fusion to presynaptic active zone membrane	4.51E-06	5.21E-04
GO: Biological Process	GO:0048791	calcium ion-regulated exocytosis of neurotransmitter	6.03E-06	6.82E-04
GO: Biological Process	GO:0051094	positive regulation of developmental process	1.01E-05	1.12E-03
GO: Biological Process	GO:1903530	regulation of secretion by cell	1.32E-05	1.44E-03
GO: Biological Process	GO:0031629	synaptic vesicle fusion to presynaptic active zone membrane	1.41E-05	1.49E-03
GO: Biological Process	GO:0050808	synapse organization	1.42E-05	1.49E-03
GO: Biological Process	GO:0007409	axonogenesis	1.54E-05	1.56E-03
GO: Biological Process	GO:0046928	regulation of neurotransmitter secretion	1.57E-05	1.56E-03
GO: Biological Process	GO:0051046	regulation of secretion	1.57E-05	1.56E-03
GO: Biological Process	GO:0014070	response to organic cyclic compound	1.92E-05	1.88E-03
GO: Biological Process	GO:0034765	regulation of ion transmembrane transport	2.39E-05	2.29E-03
GO: Biological Process	GO:0071417	cellular response to organonitrogen compound	2.49E-05	2.35E-03

GO: Biological Process	GO:0034762	regulation of transmembrane transport	2.69E-05	2.51E-03
GO: Biological Process	GO:0071495	cellular response to endogenous stimulus	2.80E-05	2.57E-03
GO: Biological Process	GO:0071407	cellular response to organic cyclic compound	2.87E-05	2.59E-03
GO: Biological Process	GO:0006198	cAMP catabolic process	3.26E-05	2.89E-03
GO: Biological Process	GO:0120035	regulation of plasma membrane bounded cell projection organization	3.41E-05	2.98E-03
GO: Biological Process	GO:0034220	ion transmembrane transport	3.96E-05	3.41E-03
GO: Biological Process	GO:0007264	small GTPase mediated signal transduction	4.07E-05	3.46E-03
GO: Biological Process	GO:0044070	regulation of anion transport	4.42E-05	3.70E-03
GO: Biological Process	GO:0007417	central nervous system development	4.65E-05	3.84E-03
GO: Biological Process	GO:0006812	cation transport	5.32E-05	4.28E-03
GO: Biological Process	GO:1901699	cellular response to nitrogen compound	5.42E-05	4.28E-03
GO: Biological Process	GO:0010243	response to organonitrogen compound	5.46E-05	4.28E-03
GO: Biological Process	GO:0031344	regulation of cell projection organization	5.49E-05	4.28E-03
GO: Biological Process	GO:0051588	regulation of neurotransmitter transport	5.88E-05	4.53E-03
GO: Biological Process	GO:0022603	regulation of anatomical structure morphogenesis	7.18E-05	5.45E-03
GO: Biological Process	GO:0022604	regulation of cell morphogenesis	7.38E-05	5.53E-03
GO: Biological Process	GO:0042981	regulation of apoptotic process	7.64E-05	5.65E-03
GO: Biological Process	GO:0043067	regulation of programmed cell death	8.49E-05	6.20E-03
GO: Biological Process	GO:0051099	positive regulation of binding	8.64E-05	6.23E-03
GO: Biological Process	GO:1901698	response to nitrogen compound	8.86E-05	6.31E-03
GO: Biological Process	GO:1903859	regulation of dendrite extension	9.72E-05	6.84E-03
GO: Biological Process	GO:0017157	regulation of exocytosis	1.06E-04	7.34E-03
GO: Biological Process	GO:0055085	transmembrane transport	1.12E-04	7.70E-03
GO: Biological Process	GO:0072659	protein localization to plasma membrane	1.17E-04	7.93E-03
GO: Biological Process	GO:0046879	hormone secretion	1.19E-04	7.93E-03
GO: Biological Process	GO:0060078	regulation of postsynaptic membrane potential	1.20E-04	7.93E-03
GO: Biological Process	GO:0008277	regulation of G protein-coupled receptor signaling pathway	1.35E-04	8.83E-03
GO: Biological Process	GO:2000026	regulation of multicellular organismal development	1.36E-04	8.83E-03
GO: Biological Process	GO:0090174	organelle membrane fusion	1.46E-04	9.34E-03
GO: Biological Process	GO:0048284	organelle fusion	1.50E-04	9.46E-03
GO: Biological Process	GO:0009719	response to endogenous stimulus	1.57E-04	9.46E-03
GO: Biological Process	GO:0007010	cytoskeleton organization	1.57E-04	9.46E-03
GO: Biological Process	GO:0006887	exocytosis	1.57E-04	9.46E-03
GO: Biological Process	GO:0140029	exocytic process	1.57E-04	9.46E-03
GO: Biological Process	GO:0099500	vesicle fusion to plasma membrane	1.57E-04	9.46E-03
GO: Biological Process	GO:0009914	hormone transport	1.64E-04	9.76E-03
GO: Biological Process	GO:0046058	cAMP metabolic process	1.92E-04	1.13E-02
GO: Biological Process	GO:0050806	positive regulation of synaptic transmission	2.00E-04	1.16E-02
GO: Biological Process	GO:0051962	positive regulation of nervous system development	2.17E-04	1.25E-02
GO: Biological Process	GO:0043069	negative regulation of programmed cell death	2.21E-04	1.26E-02
GO: Biological Process	GO:0106027	neuron projection organization	2.22E-04	1.26E-02
GO: Biological Process	GO:0009214	cyclic nucleotide catabolic process	2.36E-04	1.32E-02
GO: Biological Process	GO:0098660	inorganic ion transmembrane transport	2.38E-04	1.32E-02
GO: Biological Process	GO:0072657	protein localization to membrane	2.51E-04	1.37E-02
GO: Biological Process	GO:0006906	vesicle fusion	2.53E-04	1.37E-02
GO: Biological Process	GO:0043066	negative regulation of apoptotic process	2.54E-04	1.37E-02
GO: Biological Process	GO:0050775	positive regulation of dendrite morphogenesis	2.65E-04	1.42E-02
GO: Biological Process	GO:2000300	regulation of synaptic vesicle exocytosis	2.81E-04	1.49E-02
GO: Biological Process	GO:0030073	insulin secretion	2.91E-04	1.52E-02
GO: Biological Process	GO:1903793	positive regulation of anion transport	2.93E-04	1.52E-02
GO: Biological Process	GO:0008016	regulation of heart contraction	3.32E-04	1.71E-02
GO: Biological Process	GO:0045055	regulated exocytosis	3.42E-04	1.75E-02
GO: Biological Process	GO:0060322	head development	3.47E-04	1.76E-02
GO: Biological Process	GO:1903829	positive regulation of cellular protein localization	3.80E-04	1.91E-02
GO: Biological Process	GO:1901700	response to oxygen-containing compound	3.88E-04	1.93E-02
GO: Biological Process	GO:0060548	negative regulation of cell death	3.91E-04	1.93E-02
GO: Biological Process	GO:0034341	response to interferon-gamma	4.05E-04	1.97E-02
GO: Biological Process	GO:0007420	brain development	4.08E-04	1.97E-02
GO: Biological Process	GO:0098815	modulation of excitatory postsynaptic potential	4.11E-04	1.97E-02
GO: Biological Process	GO:1904862	inhibitory synapse assembly	4.14E-04	1.97E-02
GO: Biological Process	GO:0010941	regulation of cell death	4.19E-04	1.98E-02
GO: Biological Process	GO:0009187	cyclic nucleotide metabolic process	4.40E-04	2.05E-02
GO: Biological Process	GO:0023056	positive regulation of signaling	4.43E-04	2.05E-02
GO: Biological Process	GO:0007411	axon guidance	4.44E-04	2.05E-02
GO: Biological Process	GO:0051098	regulation of binding	4.48E-04	2.05E-02
GO: Biological Process	GO:0034097	response to cytokine	4.51E-04	2.05E-02
GO: Biological Process	GO:1903305	regulation of regulated secretory pathway	4.56E-04	2.06E-02
GO: Biological Process	GO:0097485	neuron projection guidance	4.61E-04	2.06E-02
GO: Biological Process	GO:0051960	regulation of nervous system development	4.80E-04	2.13E-02
GO: Biological Process	GO:1903861	positive regulation of dendrite extension	4.89E-04	2.15E-02
GO: Biological Process	GO:0097484	dendrite extension	5.00E-04	2.18E-02
GO: Biological Process	GO:0071346	cellular response to interferon-gamma	5.05E-04	2.19E-02
GO: Biological Process	GO:0045595	regulation of cell differentiation	5.16E-04	2.22E-02
GO: Biological Process	GO:0071345	cellular response to cytokine stimulus	5.20E-04	2.22E-02
GO: Biological Process	GO:0035914	skeletal muscle cell differentiation	5.25E-04	2.23E-02
GO: Biological Process	GO:1903778	protein localization to vacuolar membrane	5.48E-04	2.31E-02
GO: Biological Process	GO:0032092	positive regulation of protein binding	5.63E-04	2.35E-02
GO: Biological Process	GO:0031338	regulation of vesicle fusion	5.75E-04	2.39E-02
GO: Biological Process	GO:0003012	muscle system process	5.82E-04	2.40E-02
GO: Biological Process	GO:0061025	membrane fusion	5.85E-04	2.40E-02
GO: Biological Process	GO:0061762	CAMKK-AMPK signaling cascade	6.33E-04	2.57E-02
GO: Biological Process	GO:0008015	blood circulation	6.72E-04	2.71E-02
GO: Biological Process	GO:0010647	positive regulation of cell communication	6.90E-04	2.77E-02
GO: Biological Process	GO:0032412	regulation of ion transmembrane transporter activity	6.97E-04	2.77E-02
GO: Biological Process	GO:0010977	negative regulation of neuron projection development	7.27E-04	2.88E-02
GO: Biological Process	GO:0007214	gamma-aminobutyric acid signaling pathway	7.83E-04	3.07E-02
GO: Biological Process	GO:0030100	regulation of endocytosis	8.00E-04	3.10E-02
GO: Biological Process	GO:0061337	cardiac conduction	8.00E-04	3.10E-02
GO: Biological Process	GO:0099637	neurotransmitter receptor transport	8.18E-04	3.15E-02
GO: Biological Process	GO:0045597	positive regulation of cell differentiation	8.35E-04	3.19E-02
GO: Biological Process	GO:0031346	positive regulation of cell projection organization	8.45E-04	3.21E-02
GO: Biological Process	GO:0035418	protein localization to synapse	8.56E-04	3.23E-02
GO: Biological Process	GO:0016050	vesicle organization	8.76E-04	3.28E-02
GO: Biological Process	GO:1901701	cellular response to oxygen-containing compound	8.81E-04	3.28E-02
GO: Biological Process	GO:0060284	regulation of cell development	9.02E-04	3.34E-02
GO: Biological Process	GO:1990138	neuron projection extension	9.36E-04	3.44E-02
GO: Biological Process	GO:0007030	Golgi organization	9.41E-04	3.44E-02
GO: Biological Process	GO:0098662	inorganic cation transmembrane transport	1.01E-03	3.65E-02
GO: Biological Process	GO:0051130	positive regulation of cellular component organization	1.01E-03	3.65E-02
GO: Biological Process	GO:0022898	regulation of transmembrane transporter activity	1.04E-03	3.71E-02
GO: Biological Process	GO:0006813	potassium ion transport	1.06E-03	3.76E-02
GO: Biological Process	GO:0030072	peptide hormone secretion	1.07E-03	3.80E-02
GO: Biological Process	GO:0031329	regulation of cellular catabolic process	1.09E-03	3.84E-02
GO: Biological Process	GO:0006687	glycosphingolipid metabolic process	1.16E-03	4.07E-02
GO: Biological Process	GO:0060291	long-term synaptic potentiation	1.19E-03	4.12E-02
GO: Biological Process	GO:0010720	positive regulation of cell development	1.20E-03	4.13E-02
GO: Biological Process	GO:0048015	phosphatidylinositol-mediated signaling	1.22E-03	4.17E-02

GO: Biological Process	GO:2001257	regulation of cation channel activity	1.22E-03	4.17E-02
GO: Biological Process	GO:0048814	regulation of dendrite morphogenesis	1.23E-03	4.19E-02
GO: Biological Process	GO:0051412	response to corticosterone	1.24E-03	4.19E-02
GO: Biological Process	GO:0032870	cellular response to hormone stimulus	1.25E-03	4.20E-02
GO: Biological Process	GO:0017156	calcium-ion regulated exocytosis	1.27E-03	4.20E-02
GO: Biological Process	GO:0050769	positive regulation of neurogenesis	1.27E-03	4.20E-02
GO: Biological Process	GO:0060047	heart contraction	1.27E-03	4.20E-02
GO: Biological Process	GO:0014706	striated muscle tissue development	1.29E-03	4.23E-02
GO: Biological Process	GO:0099004	calmodulin dependent kinase signaling pathway	1.31E-03	4.27E-02
GO: Biological Process	GO:0048813	dendrite morphogenesis	1.36E-03	4.42E-02
GO: Biological Process	GO:0048168	regulation of neuronal synaptic plasticity	1.37E-03	4.43E-02
GO: Biological Process	GO:1904062	regulation of cation transmembrane transport	1.38E-03	4.43E-02
GO: Biological Process	GO:0048017	inositol lipid-mediated signaling	1.39E-03	4.43E-02
GO: Biological Process	GO:0007229	integrin-mediated signaling pathway	1.43E-03	4.53E-02
GO: Biological Process	GO:0098655	cation transmembrane transport	1.46E-03	4.60E-02
GO: Biological Process	GO:1903522	regulation of blood circulation	1.48E-03	4.64E-02
GO: Biological Process	GO:0072665	protein localization to vacuole	1.49E-03	4.65E-02
GO: Biological Process	GO:1902414	protein localization to cell junction	1.50E-03	4.66E-02
GO: Biological Process	GO:0061061	muscle structure development	1.57E-03	4.84E-02
GO: Biological Process	GO:0033693	neurofilament bundle assembly	1.58E-03	4.85E-02
GO: Biological Process	GO:0070201	regulation of establishment of protein localization	1.59E-03	4.86E-02
GO: Cellular Component	GO:0043005	neuron projection	1.11E-17	8.31E-15
GO: Cellular Component	GO:0045202	synapse	5.24E-16	1.96E-13
GO: Cellular Component	GO:0098794	postsynapse	1.60E-13	3.99E-11
GO: Cellular Component	GO:0030425	dendrite	2.80E-12	4.01E-10
GO: Cellular Component	GO:0097447	dendritic tree	2.80E-12	4.01E-10
GO: Cellular Component	GO:0036477	somatodendritic compartment	3.21E-12	4.01E-10
GO: Cellular Component	GO:0030424	axon	7.12E-12	7.62E-10
GO: Cellular Component	GO:0044297	cell body	6.16E-11	5.77E-09
GO: Cellular Component	GO:0043025	neuronal cell body	9.78E-11	8.14E-09
GO: Cellular Component	GO:0099572	postsynaptic specialization	5.54E-10	4.15E-08
GO: Cellular Component	GO:0097060	synaptic membrane	8.25E-10	5.62E-08
GO: Cellular Component	GO:0098984	neuron to neuron synapse	5.25E-09	3.28E-07
GO: Cellular Component	GO:0014069	postsynaptic density	1.46E-08	7.80E-07
GO: Cellular Component	GO:0032279	asymmetric synapse	1.46E-08	7.80E-07
GO: Cellular Component	GO:0098793	presynapse	2.35E-08	1.18E-06
GO: Cellular Component	GO:0098982	GABA-ergic synapse	3.62E-08	1.70E-06
GO: Cellular Component	GO:0032589	neuron projection membrane	1.15E-07	5.06E-06
GO: Cellular Component	GO:0098978	glutamatergic synapse	1.68E-07	6.99E-06
GO: Cellular Component	GO:0045211	postsynaptic membrane	3.66E-07	1.44E-05
GO: Cellular Component	GO:0043204	perikaryon	1.88E-06	7.04E-05
GO: Cellular Component	GO:0099634	postsynaptic specialization membrane	2.86E-06	1.02E-04
GO: Cellular Component	GO:1902495	transmembrane transporter complex	6.20E-06	2.11E-04
GO: Cellular Component	GO:0034702	ion channel complex	6.61E-06	2.15E-04
GO: Cellular Component	GO:0150034	distal axon	7.89E-06	2.46E-04
GO: Cellular Component	GO:1990351	transporter complex	1.25E-05	3.73E-04
GO: Cellular Component	GO:0032809	neuronal cell body membrane	2.02E-05	5.83E-04
GO: Cellular Component	GO:0099699	integral component of synaptic membrane	2.32E-05	6.43E-04
GO: Cellular Component	GO:0044304	main axon	2.57E-05	6.87E-04
GO: Cellular Component	GO:0044298	cell body membrane	3.45E-05	8.91E-04
GO: Cellular Component	GO:0099060	integral component of postsynaptic specialization membrane	3.77E-05	9.40E-04
GO: Cellular Component	GO:0044305	calyx of Held	4.71E-05	1.13E-03
GO: Cellular Component	GO:0034703	cation channel complex	4.98E-05	1.13E-03
GO: Cellular Component	GO:0008021	synaptic vesicle	4.99E-05	1.13E-03
GO: Cellular Component	GO:0030133	transport vesicle	5.17E-05	1.14E-03
GO: Cellular Component	GO:0098948	intrinsic component of postsynaptic specialization membrane	6.27E-05	1.34E-03
GO: Cellular Component	GO:0099240	intrinsic component of synaptic membrane	6.44E-05	1.34E-03
GO: Cellular Component	GO:0098796	membrane protein complex	7.26E-05	1.47E-03
GO: Cellular Component	GO:0030673	axolemma	1.01E-04	2.00E-03
GO: Cellular Component	GO:0070382	exocytic vesicle	1.27E-04	2.44E-03
GO: Cellular Component	GO:0032590	dendrite membrane	1.51E-04	2.83E-03
GO: Cellular Component	GO:0030658	transport vesicle membrane	1.59E-04	2.90E-03
GO: Cellular Component	GO:0099055	integral component of postsynaptic membrane	1.75E-04	3.11E-03
GO: Cellular Component	GO:0031256	leading edge membrane	2.05E-04	3.58E-03
GO: Cellular Component	GO:0098936	intrinsic component of postsynaptic membrane	2.91E-04	4.88E-03
GO: Cellular Component	GO:0005815	microtubule organizing center	2.97E-04	4.88E-03
GO: Cellular Component	GO:0031252	cell leading edge	3.00E-04	4.88E-03
GO: Cellular Component	GO:0005794	Golgi apparatus	3.39E-04	5.31E-03
GO: Cellular Component	GO:0098839	postsynaptic density membrane	3.40E-04	5.31E-03
GO: Cellular Component	GO:0048471	perinuclear region of cytoplasm	4.06E-04	6.20E-03
GO: Cellular Component	GO:0070161	anchoring junction	4.24E-04	6.35E-03
GO: Cellular Component	GO:0042734	presynaptic membrane	4.38E-04	6.43E-03
GO: Cellular Component	GO:0031594	neuromuscular junction	4.77E-04	6.87E-03
GO: Cellular Component	GO:0097440	apical dendrite	5.96E-04	8.43E-03
GO: Cellular Component	GO:0043197	dendritic spine	6.21E-04	8.61E-03
GO: Cellular Component	GO:0005769	early endosome	6.52E-04	8.88E-03
GO: Cellular Component	GO:0019897	extrinsic component of plasma membrane	6.93E-04	9.27E-03
GO: Cellular Component	GO:0098797	plasma membrane protein complex	7.06E-04	9.28E-03
GO: Cellular Component	GO:0044309	neuron spine	7.56E-04	9.77E-03
GO: Cellular Component	GO:0030285	integral component of synaptic vesicle membrane	8.37E-04	1.06E-02
GO: Cellular Component	GO:1902711	GABA-A receptor complex	8.59E-04	1.07E-02
GO: Cellular Component	GO:0098688	parallel fiber to Purkinje cell synapse	9.37E-04	1.15E-02
GO: Cellular Component	GO:0031201	SNARE complex	1.04E-03	1.26E-02
GO: Cellular Component	GO:0044306	neuron projection terminus	1.10E-03	1.31E-02
GO: Cellular Component	GO:0005768	endosome	1.23E-03	1.44E-02
GO: Cellular Component	GO:1902710	GABA receptor complex	1.28E-03	1.47E-02
GO: Cellular Component	GO:0043679	axon terminus	1.31E-03	1.49E-02
GO: Cellular Component	GO:0099501	exocytic vesicle membrane	1.35E-03	1.49E-02
GO: Cellular Component	GO:0030672	synaptic vesicle membrane	1.35E-03	1.49E-02
GO: Cellular Component	GO:0099738	cell cortex region	1.42E-03	1.54E-02
GO: Cellular Component	GO:0005814	centriole	1.44E-03	1.54E-02
GO: Cellular Component	GO:0099569	presynaptic cytoskeleton	1.81E-03	1.91E-02
GO: Cellular Component	GO:0097478	leaflet of membrane bilayer	2.35E-03	2.40E-02
GO: Cellular Component	GO:0098552	side of membrane	2.35E-03	2.40E-02
GO: Cellular Component	GO:0048786	presynaptic active zone	2.37E-03	2.40E-02
GO: Cellular Component	GO:0005925	focal adhesion	2.44E-03	2.44E-02
GO: Cellular Component	GO:0000242	pericentriolar material	2.51E-03	2.47E-02
GO: Cellular Component	GO:0005802	trans-Golgi network	2.54E-03	2.47E-02
GO: Cellular Component	GO:0034705	potassium channel complex	2.68E-03	2.58E-02
GO: Cellular Component	GO:0045335	phagocytic vesicle	2.79E-03	2.65E-02
GO: Cellular Component	GO:0060076	excitatory synapse	2.84E-03	2.66E-02
GO: Cellular Component	GO:0030017	sarcomere	3.04E-03	2.81E-02
GO: Cellular Component	GO:0099160	postsynaptic intermediate filament cytoskeleton	3.16E-03	2.87E-02
GO: Cellular Component	GO:0019898	extrinsic component of membrane	3.24E-03	2.87E-02
GO: Cellular Component	GO:0030055	cell-substrate junction	3.27E-03	2.87E-02
GO: Cellular Component	GO:0098562	cytoplasmic side of membrane	3.27E-03	2.87E-02
GO: Cellular Component	GO:0009898	cytoplasmic side of plasma membrane	3.30E-03	2.87E-02

GO: Cellular Component	GO:0015630	microtubule cytoskeleton	3.40E-03	2.93E-02
GO: Cellular Component	GO:0030426	growth cone	3.52E-03	3.00E-02
GO: Cellular Component	GO:0005813	centrosome	3.94E-03	3.29E-02
GO: Cellular Component	GO:0045121	membrane raft	3.96E-03	3.29E-02
GO: Cellular Component	GO:0098857	membrane microdomain	4.06E-03	3.34E-02
GO: Cellular Component	GO:0005938	cell cortex	4.31E-03	3.51E-02
GO: Cellular Component	GO:0030427	site of polarized growth	4.51E-03	3.64E-02
GO: Cellular Component	GO:0098563	intrinsic component of synaptic vesicle membrane	4.70E-03	3.74E-02
GO: Cellular Component	GO:0008076	voltage-gated potassium channel complex	5.10E-03	4.01E-02
GO: Cellular Component	GO:0097418	neurofibrillary tangle	5.19E-03	4.01E-02
GO: Cellular Component	GO:0098826	endoplasmic reticulum tubular network membrane	5.19E-03	4.01E-02
GO: Cellular Component	GO:0099056	integral component of presynaptic membrane	5.87E-03	4.48E-02
GO: Cellular Component	GO:0030016	myofibril	6.11E-03	4.62E-02
GO: Cellular Component	GO:0034707	chloride channel complex	6.59E-03	4.93E-02
Pathway	1268763	Neuronal System	1.61E-08	3.99E-05
Pathway	1269145	Neurotoxicity of clostridium toxins	2.87E-06	3.57E-03
Pathway	952859	Oxytocin signaling pathway	1.34E-05	1.11E-02
Pathway	P05734	Synaptic vesicle trafficking	2.29E-05	1.37E-02
Pathway	552665	Morphine addiction	2.75E-05	1.37E-02
Pathway	698773	Circadian entrainment	4.56E-05	1.44E-02
Pathway	M39432	TGF-beta Signaling Pathway	5.18E-05	1.44E-02
Pathway	1269505	MAPK1 (ERK2) activation	5.50E-05	1.44E-02
Pathway	M39511	Serotonin and anxiety	6.04E-05	1.44E-02
Pathway	1268766	Transmission across Chemical Synapses	7.11E-05	1.44E-02
Pathway	M39854	mBDNF and proBDNF regulation of GABA neurotransmission	7.58E-05	1.44E-02
Pathway	138040	IFN-gamma pathway	7.58E-05	1.44E-02
Pathway	M39475	Myometrial Relaxation and Contraction Pathways	7.94E-05	1.44E-02
Pathway	M237	Signaling events mediated by VEGFR1 and VEGFR2	8.11E-05	1.44E-02
Pathway	908257	Adrenergic signaling in cardiomyocytes	1.18E-04	1.90E-02
Pathway	137932	IL6-mediated signaling events	1.22E-04	1.90E-02
Pathway	1269144	Uptake and actions of bacterial toxins	1.39E-04	2.04E-02
Pathway	M39818	IL-18 signaling pathway	1.65E-04	2.21E-02
Pathway	1269151	Toxicity of botulinum toxin type D (BoNT/D)	1.77E-04	2.21E-02
Pathway	1269152	Toxicity of botulinum toxin type F (BoNT/F)	1.77E-04	2.21E-02
Pathway	PW:0000542	adenosine monophosphate-activated protein kinase (AMPK) signaling	2.03E-04	2.40E-02
Pathway	M183	IL6-mediated signaling events	2.18E-04	2.47E-02
Pathway	1017634	cAMP signaling pathway	2.58E-04	2.76E-02
Pathway	799177	Estrogen signaling pathway	2.66E-04	2.76E-02
Pathway	M39562	Oncostatin M Signaling Pathway	3.38E-04	3.36E-02
Pathway	M39822	Synaptic signaling pathways associated with autism spectrum disorder	3.67E-04	3.44E-02
Pathway	83085	Long-term potentiation	3.75E-04	3.44E-02
Pathway	M39334	EGF/EGFR Signaling Pathway	4.01E-04	3.44E-02
Pathway	547607	Amphetamine addiction	4.15E-04	3.44E-02
Pathway	M39684	Human Thyroid Stimulating Hormone (TSH) signaling pathway	4.15E-04	3.44E-02
Pathway	M3115	Long-term potentiation	5.06E-04	4.06E-02
Pathway	1269952	Ligand-gated ion channel transport	5.28E-04	4.07E-02
Pathway	1268786	Neurotransmitter Receptor Binding And Downstream Transmission In The Postsynaptic Cell	5.40E-04	4.07E-02
Pathway	M161	IFN-gamma pathway	5.95E-04	4.35E-02
Pathway	469199	Dopaminergic synapse	6.76E-04	4.67E-02
Pathway	1269759	Amplification of signal from the kinetochores	7.20E-04	4.67E-02
Pathway	1269760	Amplification of signal from unattached kinetochores via a MAD2 inhibitory signal	7.20E-04	4.67E-02
Pathway	M39329	Calcium Regulation in the Cardiac Cell	7.28E-04	4.67E-02
Pathway	217716	Cholinergic synapse	7.75E-04	4.67E-02
Pathway	83065	Axon guidance	7.87E-04	4.67E-02
Pathway	1268805	CREB phosphorylation through the activation of CaMKII	8.77E-04	4.67E-02
Pathway	M111	Roles of b-arrestin-dependent Recruitment of Src Kinases in GPCR Signaling	8.77E-04	4.67E-02
Pathway	M39656	IL-6 signaling pathway	8.83E-04	4.67E-02
Pathway	213818	Glutamatergic synapse	8.89E-04	4.67E-02
Pathway	M39616	PDGFR-beta pathway	8.93E-04	4.67E-02
Pathway	1268801	CREB phosphorylation through the activation of Ras	8.93E-04	4.67E-02
Pathway	138044	Trk receptor signaling mediated by the MAPK pathway	8.93E-04	4.67E-02
Pathway	1427850	Interactions of neurexins and neuroligins at synapses	9.02E-04	4.67E-02
Pathway	M22063	Stat3 Signaling Pathway	9.35E-04	4.75E-02
Disease	C0036341	Schizophrenia	1.48E-06	9.62E-03
Disease	C0014548	Epilepsy, Generalized	4.97E-06	1.30E-02
Disease	C0014544	Epilepsy	6.02E-06	1.30E-02
Disease	C0007758	Cerebellar Ataxia	9.69E-06	1.57E-02
Disease	C0013384	Dyskinetic syndrome	2.89E-05	3.58E-02
Disease	C0004134	Ataxia	3.31E-05	3.58E-02
Disease	C0024636	Malocclusion	8.00E-05	4.81E-02
Disease	C1862941	Amyotrophic Lateral Sclerosis, Sporadic	8.87E-05	4.81E-02
Disease	C4551993	Amyotrophic Lateral Sclerosis, Familial	8.87E-05	4.81E-02
Disease	C0086237	Epilepsy, Cryptogenic	8.89E-05	4.81E-02
Disease	C0751111	Awakening Epilepsy	8.89E-05	4.81E-02
Disease	C0236018	Aura	8.89E-05	4.81E-02

Abbreviations: PLS, partial least squares; FDR-BH, the Benjamini and Hochberg method for false discovery rate; GO, gene ontology.

Enrichment results of the PLS- genes

Category	ID	Name	*P* value	*q* value (FDR-BH correction)
GO: Molecular Function	GO:0005216	ion channel activity	3.25E-06	3.77E-03
GO: Molecular Function	GO:0022836	gated channel activity	6.84E-06	3.77E-03
GO: Molecular Function	GO:0005261	cation channel activity	9.13E-06	3.77E-03
GO: Molecular Function	GO:0015267	channel activity	2.16E-05	5.57E-03
GO: Molecular Function	GO:0022803	passive transmembrane transporter activity	2.25E-05	5.57E-03
GO: Molecular Function	GO:0016247	channel regulator activity	2.39E-04	4.26E-02
GO: Molecular Function	GO:0022839	ion gated channel activity	2.41E-04	4.26E-02
GO: Biological Process	GO:0007267	cell-cell signaling	2.98E-07	1.87E-03
GO: Biological Process	GO:0001822	kidney development	5.55E-06	1.63E-02
GO: Biological Process	GO:0072001	renal system development	1.14E-05	1.63E-02
GO: Biological Process	GO:0070839	metal ion export	1.30E-05	1.63E-02
GO: Biological Process	GO:0099537	trans-synaptic signaling	1.53E-05	1.63E-02
GO: Biological Process	GO:0003018	vascular process in circulatory system	1.56E-05	1.63E-02
GO: Biological Process	GO:0030048	actin filament-based movement	2.81E-05	2.06E-02
GO: Biological Process	GO:0099536	synaptic signaling	2.84E-05	2.06E-02
GO: Biological Process	GO:0060343	trabecula formation	3.91E-05	2.06E-02
GO: Biological Process	GO:0098916	anterograde trans-synaptic signaling	4.37E-05	2.06E-02
GO: Biological Process	GO:0007268	chemical synaptic transmission	4.37E-05	2.06E-02
GO: Biological Process	GO:0001655	urogenital system development	4.49E-05	2.06E-02
GO: Biological Process	GO:0030029	actin filament-based process	4.60E-05	2.06E-02
GO: Biological Process	GO:1904062	regulation of cation transmembrane transport	4.70E-05	2.06E-02
GO: Biological Process	GO:0010811	positive regulation of cell-substrate adhesion	5.03E-05	2.06E-02
GO: Biological Process	GO:0019336	phenol-containing compound catabolic process	5.29E-05	2.06E-02
GO: Biological Process	GO:0019229	regulation of vasoconstriction	5.58E-05	2.06E-02
GO: Biological Process	GO:0097623	potassium ion export across plasma membrane	6.25E-05	2.18E-02
GO: Biological Process	GO:0031589	cell-substrate adhesion	7.16E-05	2.36E-02
GO: Biological Process	GO:0140115	export across plasma membrane	8.19E-05	2.57E-02
GO: Biological Process	GO:0070252	actin-mediated cell contraction	8.61E-05	2.57E-02
GO: Biological Process	GO:0060372	regulation of atrial cardiac muscle cell membrane repolarization	9.34E-05	2.66E-02
GO: Biological Process	GO:0044057	regulation of system process	1.20E-04	3.16E-02
GO: Biological Process	GO:0030201	heparan sulfate proteoglycan metabolic process	1.21E-04	3.16E-02
GO: Biological Process	GO:0034762	regulation of transmembrane transport	1.29E-04	3.23E-02
GO: Biological Process	GO:1901700	response to oxygen-containing compound	1.51E-04	3.64E-02
GO: Biological Process	GO:0003205	cardiac chamber development	1.59E-04	3.67E-02
GO: Biological Process	GO:0010647	positive regulation of cell communication	1.64E-04	3.67E-02
GO: Biological Process	GO:0023056	positive regulation of signaling	1.77E-04	3.82E-02
GO: Biological Process	GO:0098660	inorganic ion transmembrane transport	2.07E-04	4.28E-02
GO: Biological Process	GO:0023061	signal release	2.20E-04	4.28E-02
GO: Biological Process	GO:0034765	regulation of ion transmembrane transport	2.25E-04	4.28E-02
GO: Biological Process	GO:0048585	negative regulation of response to stimulus	2.25E-04	4.28E-02
GO: Biological Process	GO:0034446	substrate adhesion-dependent cell spreading	2.33E-04	4.30E-02
GO: Biological Process	GO:0032409	regulation of transporter activity	2.45E-04	4.39E-02
GO: Biological Process	GO:1901701	cellular response to oxygen-containing compound	2.55E-04	4.44E-02
GO: Biological Process	GO:0022898	regulation of transmembrane transporter activity	2.83E-04	4.79E-02
GO: Biological Process	GO:1903522	regulation of blood circulation	2.90E-04	4.79E-02
GO: Biological Process	GO:0090162	establishment of epithelial cell polarity	3.07E-04	4.94E-02
GO: Cellular Component	GO:0045202	synapse	1.85E-07	1.41E-04
GO: Cellular Component	GO:0031226	intrinsic component of plasma membrane	1.51E-06	5.79E-04
GO: Cellular Component	GO:0098793	presynapse	1.50E-05	3.22E-03
GO: Cellular Component	GO:0005887	integral component of plasma membrane	1.69E-05	3.22E-03
GO: Cellular Component	GO:0034702	ion channel complex	2.61E-05	3.98E-03
GO: Cellular Component	GO:1990351	transporter complex	7.19E-05	8.04E-03
GO: Cellular Component	GO:0008076	voltage-gated potassium channel complex	7.37E-05	8.04E-03
GO: Cellular Component	GO:1902495	transmembrane transporter complex	8.63E-05	8.24E-03
GO: Cellular Component	GO:0034703	cation channel complex	1.19E-04	9.02E-03
GO: Cellular Component	GO:0030659	cytoplasmic vesicle membrane	1.29E-04	9.02E-03
GO: Cellular Component	GO:0012506	vesicle membrane	1.30E-04	9.02E-03
GO: Cellular Component	GO:0034705	potassium channel complex	2.48E-04	1.58E-02
GO: Cellular Component	GO:0043005	neuron projection	2.71E-04	1.59E-02
GO: Cellular Component	GO:0097060	synaptic membrane	4.53E-04	2.38E-02
GO: Cellular Component	GO:0070382	exocytic vesicle	4.67E-04	2.38E-02
GO: Cellular Component	GO:0042383	sarcolemma	6.14E-04	2.93E-02
GO: Cellular Component	GO:0016528	sarcoplasm	1.14E-03	4.40E-02
GO: Cellular Component	GO:0090741	pigment granule membrane	1.15E-03	4.40E-02
GO: Cellular Component	GO:0045009	chitosome	1.15E-03	4.40E-02
GO: Cellular Component	GO:0033162	melanosome membrane	1.15E-03	4.40E-02
GO: Cellular Component	GO:0042734	presynaptic membrane	1.28E-03	4.50E-02
GO: Cellular Component	GO:0031252	cell leading edge	1.30E-03	4.50E-02
Disease	C0023893	Liver Cirrhosis, Experimental	8.39E-06	3.76E-02
Disease	C1135196	Heart Failure, Diastolic	1.04E-05	3.76E-02

Abbreviations: PLS, partial least squares; FDR-BH, the Benjamini and Hochberg method for false discovery rate; GO, gene ontology.
