# Supplementary material for: Functional hierarchy of the angular gyrus and its underlying genetic architecture
Source: Hum Brain Mapp. 2023 Feb 28;44(7):2815–28. doi: 10.1002/hbm.26247 (PMC10089092; doi:10.1002/hbm.26247)
Supplement: Supplementary file 4 — FILE S3. Tissue‐specific expression for the PLS+ and PLS− genes [file HBM-44-2815-s002.pdf]

Tissue-specific expression for the PLS+ and PLS- genes

Tissues	PLS+ genes	PLS- genes
Adipose Tissue	1.000	0.010
Adrenal Gland	0.052	0.007
Blood	1.000	0.182
Blood Vessel	2.750×10^{-4}	1.191×10^{-8}
Brain	1.863×10^{-14}	1.191×10^{-8}
Breast	1.000	0.134
Colon	1.000	0.240
Esophagus	1.000	0.260
Fallopian Tube	0.057	5.089×10^{-5}
Heart	0.057	0.005
Kidney	1.000	0.283
Liver	1.000	0.383
Lung	1.000	0.260
Muscle	0.002	0.664
Nerve	0.030	0.014
Ovary	0.541	5.283×10^{-7}
Pancreas	1.000	0.185
Pituitary	0.002	5.449×10^{-8}
Prostate	1.000	0.344
Skin	1.000	0.219
Stomach	1.000	0.219
Testis	1.000	0.842
Thyroid	1.000	0.014
Uterus	0.609	3.945×10^{-6}
Vagina	1.000	0.219

The values in the tables are the Fisher's exact *P* values corrected by the FDR-BH method.

Abbreviations: AG, angular gyrus; PLS, partial least squares; FDR-BH, the Benjamini and Hochberg method for false discovery rate.

Cell type-specific expression for the PLS+ and PLS- genes		
Cell types	PLS+ genes	PLS- genes
Oligodendrocyte progenitor cell	0.979	0.087
Immune cell	0.771	7.576×10^{-4}
Neuron_Pnoc+	0.079	0.063
Neuron_Ntsr+	0.979	7.576×10^{-4}
Neuron_Glt25d2	0.079	7.576×10^{-4}
Astrocytes	0.996	0.383
Neuron_Cort+	0.495	0.064
Myelinating oligodendrocyte	0.771	0.517
The values in the tables are the Fisher's exact *P* values corrected by the FDR-BH method.		
Abbreviations: AG, angular gyrus; PLS, partial least squares; FDR-BH, the Benjamini and Hochberg method for false discovery rate.		

Temporal-specific expression for the PLS+ and PLS- genes		
Developmental stages	PLS+ genes	PLS- genes
Cortex.Early.Fetal	1.000	0.994
Cortex.Early.Mid.Fetal	0.698	7.658×10^{-4}
Cortex.Late.Mid.Fetal	0.940	1.090×10^{-5}
Cortex.Late.Fetal	0.585	3.331×10^{-5}
Cortex.Neonatal.Early.Infancy	0.010	3.376×10^{-7}
Cortex.Late.Infancy	0.698	0.412
Cortex.Early.Childhood	0.185	0.207
Cortex.Middle.Late.Childhood	0.001	0.319
Cortex.Adolescence	1.184×10^{-9}	0.061
Cortex.Young.Adulthood	9.552×10^{-9}	0.030
The values in the tables are the Fisher's exact *P* values corrected by the FDR-BH method.		
Abbreviations: AG, angular gyrus; PLS, partial least squares; FDR-BH, the Benjamini and Hochberg method for false discovery rate.		
